# Perceptions of U.S. and Canadian maple syrup producers toward climate change, its impacts, and potential adaptation measures

**DOI:** 10.1371/journal.pone.0215511

**Published:** 2019-04-25

**Authors:** Simon Legault, Daniel Houle, Antoine Plouffe, Aitor Ameztegui, Diane Kuehn, Lisa Chase, Anne Blondlot, Timothy D. Perkins

**Affiliations:** 1 Ouranos, Consortium on Regional Climatology and Adaptation to Climate Change, Montréal, Québec, Canada; 2 Direction de la recherche forestière (DRF), Ministère des Forêts, de la Faune et des Parcs (MFFP), Québec, Québec, Canada; 3 Department of Agriculture and Forest Engineering (EAGROF), University of Lleida, Lleida, Spain; 4 Forest Sciences Center of Catalonia (CTFC), Solsona, Spain; 5 State University of New York College of Environmental Science and Forestry, Syracuse, New York, United States of America; 6 Vermont Tourism Research Center, University of Vermont Extension, Brattleboro, Vermont, United States of America; 7 Proctor Maple Research Center, Department of Plant Biology, College of Agriculture & Life Sciences, University of Vermont, Burlington, Vermont, United States of America; Woods Hole Oceanographic Institution, UNITED STATES

## Abstract

The production of maple syrup is an important cultural and economic activity directly related to the climate of northeastern North America. As a result, there are signs that climate change could have negative impacts on maple syrup production in the next decades, particularly for regions located at the southern margins of the sugar maple (*Acer saccharum* Marsh.) range. The purpose of this survey study is to present the beliefs and opinions of maple syrup producers of Canada (N = 241) and the U.S. (N = 113) on climate change in general, its impacts on sugar maple health and maple syrup production, and potential adaptation measures. Using conditional inference classification trees, we examined how the socio-economic profile of respondents and the geographic location and size of respondents’ sugar bushes shaped the responses of survey participants. While a majority (75%) of respondents are confident that the average temperature on Earth is increasing, less than half (46%) believe that climate change will have negative impacts on maple syrup yield in the next 30 years. Political view was a significant predictor of these results, with respondents at the right right and center-right of the political spectrum being less likely to believe in climate change and less likely to anticipate negative effects of climate change on maple syrup production. In addition, 77% of the participants indicated an interest in adopting adaptation strategies if those could increase maple syrup production. This interest was greater for respondents using vacuum tubing for sap collection than other collection methods. However, for many respondents (particularly in Canada), lack of information was identified as a constraint limiting adaptation to climate change.

## Introduction

Maple syrup is a unique and important non-timber forest product in northeastern North America. Its production, probably originating with the indigenous population, continues to be a cultural tradition and a source of income for many family-based businesses in Canada and the United States [1,2]. Maple syrup is produced by the concentration of sap of maple trees by boiling (now often preceded by reverse osmosis), and thus annual yield depends on the volume of sap harvested and its sweetness [3].

The sap volume harvested each season is determined by the harvesting methods utilized [4], and most importantly, by climate. Indeed, the strongest and best-understood factor affecting sap flow is temperature: water uptake from the soil and sap exudation, which involves alternating negative and positive pressure in the trunk and branches of the tree, is favored by cold nights (˂ 0 °C) followed by thaws during days (~ 3–7 °C) [5]. Depending on regional climate, these freeze-thaw cycles occur between February and April, but the beginning, end, and duration of the sap flow period can vary widely from year to year [6]. The tapping season generally ends with bud break and the development of microorganisms in taps when temperatures become warm (˃ 10 °C) for several days [7].

**Fig 1:**
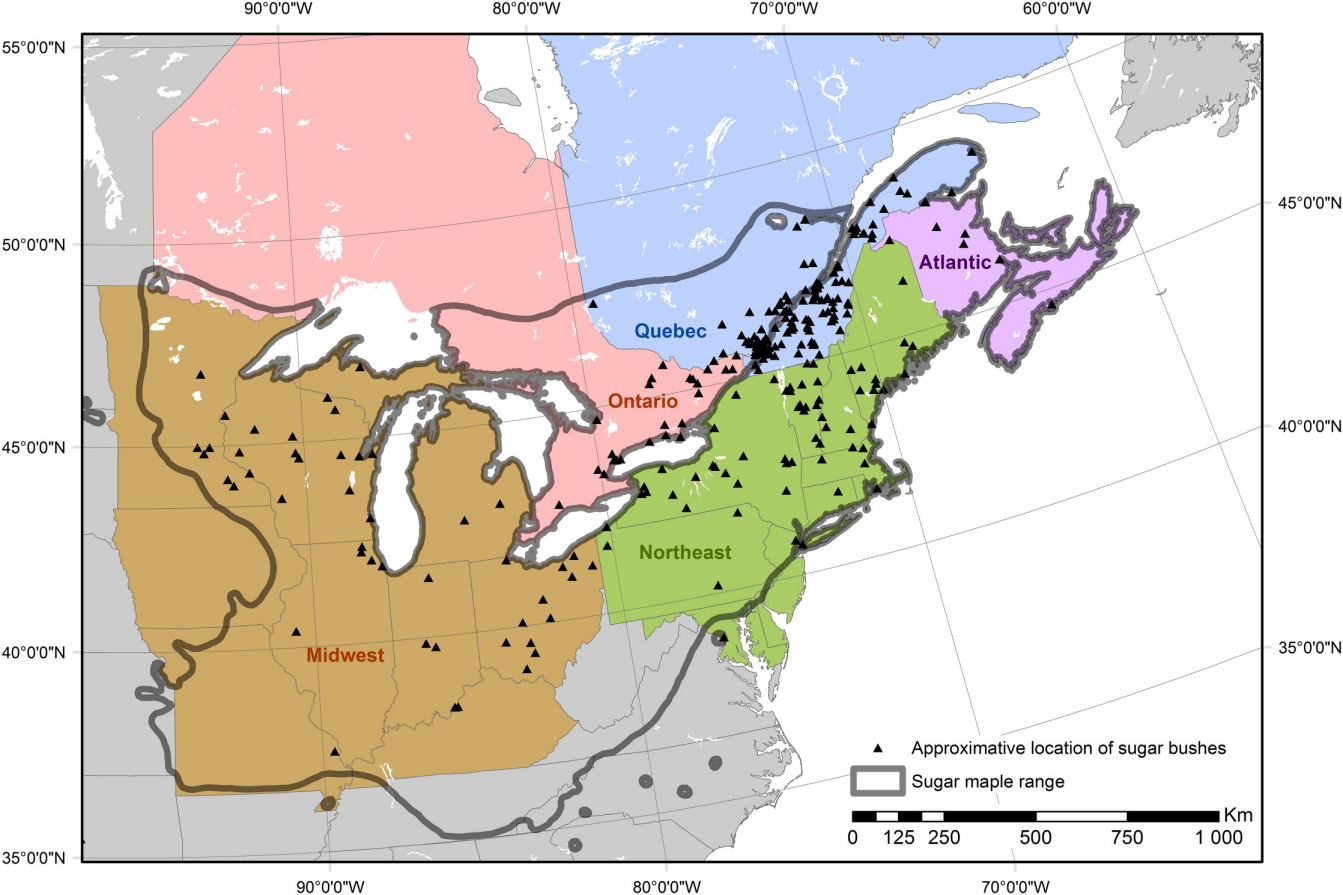
Study area. Sugar maple range (adapted from [18,19]), maple syrup regions as defined by Murphy et al. [20], and approximate location of sugar bushes whose owners participated in the survey presented in this document. The locations are based on the postal code provided by the respondents and do not necessarily represent the exact location of their sugar bush.

Sap sweetness (i.e., sugar content) is influenced by tree health and the maple species tapped. Healthy trees with large crowns and high growth rates tend to provide sweeter sap [8]. Sap from the sugar maple (*Acer saccharum* Marsh.), whose range covers southeastern Canada and the northeastern United States (Fig 1), is preferred for maple syrup production because its sugar concentration is superior to other species such as the red maple (*Acer rubrum* L.) [9,10]. Warm summer temperatures (which stimulate maple growth) and cold winter temperatures increase sap sugar concentrations in the following sap flow season [11].

Several phenomena can also affect the health of sugar maple trees and potentially reduce sap sweetness and syrup yield. These include droughts [12,13], pest outbreaks [14,15], soil fertility [16], acid depositions and air pollution, forest management, and stand maturation [17].

Because of the close link between climate and maple syrup production [11,21], climate change is expected to affect the maple syrup industry. Anticipated changes include increased summer and winter temperatures, a decrease in snow cover duration, and a time shift in the freeze-thaw period [22]. Inverse relationships between syrup production and average temperatures from January to April in Northeastern U.S. states have already been detected [23]. The projections of Skinner et al. [24] indicate that warming winter temperatures will result in a shorter sap flow period if traditional tapping schedules are maintained. The results of Duchesne et al. [25] are somewhat similar, but indicate that decreases in maple syrup yields could be avoided in Quebec if producers shift the tapping period 12 days earlier by 2050 and 19 days earlier by 2100. Finally, the study of Guilbert et al. [26] projects a decrease of 7–11 days suitable for maple syrup production by 2040–2069 and 2070–2099 in the Lake Champlain Basin, and that suitable days will occur earlier in the season.

With regard to all these potential threats, concerns have been raised about the future of maple syrup production in both Canada and the U.S. [20,27–31]. The survey of Mozumber et al. (N = 102) [30] suggests that a majority of North American producers believe that the quantity and quality of maple sap is decreasing because of climate change, and about one-third missed the first sap flow of the season in recent years. In the province of Quebec, the survey of Rondeau [27] revealed that 66% of respondents (N = 63) where highly or moderately worried about the profitability of production in the face of climate change. In Ontario, a small survey (N = 33) completed in 2009 showed that 50% of the respondents already observed the effects of climate change on their production [28]. In the U.S., a recent survey (N = 264) revealed that more than half of the maple syrup producers from New York and Vermont are apprehensive about climate change [31]. Contrarily, another recent survey (N = 353), focusing on maple syrup producers from Minnesota, Wisconsin and Michigan, reported that only a small percentage of respondents expressed concerns about climate change impacts on their operations [32].

Unfortunately, previous surveys documenting maple syrup producers’ perceptions towards climate change all used very different survey questions and methodologies, and used either small sample sizes [20,27,28], or a sampling pool from a limited geographic area [31–33]. In addition, generalizations using previous surveys would underrepresent the views of Quebec’s producers, who are responsible for approximately 70% of the world maple syrup production [34]. Altogether, these factors prevent the establishment of a clear global picture for the maple syrup industry. Furthermore, because the impacts of climate change on maple syrup production are expected to vary geographically, adaptation measures to climate change could potentially be implemented differently according to regional specificities. Thus, understanding the variations in producers’ perceptions across a climate gradient is crucial.

In the past years, there have been extensive efforts to examine the variables that are most closely correlated with acceptance of anthropogenic climate change [35–39]. In a recent meta-analysis, Hornsey et al. [39] synthetized the results of 171 academic studies across 56 countries and concluded that the factor that correlates most with climate change belief is political view. In Canada and the U.S., this factor is very important for explaining the acceptance of anthropogenic climate change, with conservatives being less likely to believe in climate change than liberals [40–42]. In the forest sector in particular, it has been shown that concerns regarding climate change vary with political views and geographical context (e.g. the province of origin; [43]), but also with individual factors such as specific forest utilisation, knowledge of forestry, and level of economic dependence on forests [44]. It has also been shown that forest managers’ beliefs about climate change are correlated with their willingness to try specific adaptation or mitigation measures [45]. These studies thus show that determining the main factors influencing maple syrup producers’ perceptions of climate change is very important for the identification of potential adaptation strategies for the maple syrup industry in the face of climate change.

The primary objective of this study was to examine the perceptions of maple syrup producers from Canada and the U.S. toward (i) climate change in general, (ii) its effects on sugar bushes health and productivity, and (iii) potential adaptation measures. For this purpose, we conducted an online survey addressed to maple syrup producers from all producing regions of Canada and the U.S. Because maple syrup production is highly climate-dependent [11,21], we hypothesized that maple syrup producers would generally be more sensitive to the concerns raised by the scientific community in relation to climate change than the general population. We therefore expected that maple producers’ perceptions would not be significantly related to their political views. Also, given the ongoing shift of the climatic envelope suitable for maple syrup production toward northward latitudes [24], we expected an effect of spatial context on producer’s opinions. More specifically, we expected that producers in southerly regions of our study area, being potentially more affected by climate change impacts than producers from more northerly regions, would have higher levels of concern about future climate change impacts on maple syrup production. Finally, as implementing adaptation measures can be viewed as costly for producers [31], we hypothesized that factors associated with the size of the sugar bush would be important in predicting responses rates for questions or statements associated with adaptation measures, with large-scale producers more inclined to adopt adaptation measures than small-scale producers.

## Methods

### Survey design and distribution

The survey used in this study was distributed to Canadian and American maple syrup producers (specifically those who were members of maple producer associations) via an internet-based survey platform (SurveyGizmo, Boulder, CO, USA). To seek responses from as many respondents as possible, we contacted the maple syrup producers’ associations from all regions of the study area (delineated by the sugar maple’s range; Fig 1) and asked them to send a link of the survey to their members and publicize the survey through websites, social media pages, and letters to members. We also publicized the survey on various maple producers’ forums in Canada and the U.S. (e.g. MapleTrader.com, Lessucriers.com, and Sugarbush.info) and through paper journals (e.g. Maple News and Maple Digest).

The survey was divided in three sections: (i) questions and statements about general perceptions of climate change (eight statements), (ii) climate change impact on maple syrup production (11 statements), and (iii) adaptation strategies to climate change (13 statements). For most statements, respondents specified their degree of agreement using a 5 or 7-grade Likert scale. For each proposed adaptation strategy, respondents specified if it was efficient, and if they planned to use it, or were already using it. For some questions, a Yes/No answer was collected. Furthermore, we recorded as potential predictor variables socio-economic information about the respondents (age, gender, education level, and political view), characteristics of the sugar bush (number of taps, age of the largest maples, harvesting method, years of experience in the maple syrup industry, someone to take over once retired, percentage of household income contributed by maple sugar business, investment planning during the next 10 years, and yield in 2016), and sugar bush location (country, region, latitude, and longitude). Because of the low number of respondents in many provinces/states, we grouped respondents according to regions as delimited by Murphy et al. [20] (Canada: Ontario, Québec, and Atlantic; U.S.: Northeast, and Midwest-Southeast; Fig 1). The full questionnaire and the distribution of responses for each question and statement can be found in the Supplementary Material 1.

### Data analyses

To determine the relative influence of predictors (socio-economic information about the respondents, characteristics of the sugar bush, and its spatial location) in explaining the distribution of responses by survey respondents, we used a conditional inference classification tree approach. In contrast with traditional approaches such as multiple regression, this technique has the advantage of accommodating a large number of explanatory variables, even if they are highly correlated [46,47]. In addition, this approach makes it possible to include variables of different types (nominal, ordinal, interval and quantitative), as in our dataset. This approach has been used successfully in several research areas [48–50], including studies identifying the factors influencing responses in climate change-related surveys [36,43].

As suggested by Lee et al. [36], we used a two-step approach during data analyses in order to avoid an excessive number of explanatory variables in each tree model (one for each question/statement of the survey). First, we determined the most influential variable(s) using a random forests permutation-based procedure, as implemented in the R package *VSURF* [51]. This process ranks the predictors iteratively based on an importance metric, and returns a small subset of variables with minimal redundancy between them. Second, a single conditional inference tree was produced for each question of the survey, using as input only the variables selected during the first step. For this procedure, we used the R function *ctree*, as implemented in the *partykit* package [52]. In summary, this recursive function performs univariate divisions of the response variable based on the values of a set of covariates. For this step, we used a random training subset consisting of 80% of the respondents. A default threshold of *p* = 0.05 was used to determine if variables were dropped from the model. Finally, for each question/statement of the survey, we calculated the classification accuracy (CA; [36]) of each conditional tree by calculating the proportion of correct predictions using the remaining 20% of the dataset.

### Results and discussion

Using an online survey, we examined the perceptions of maple syrup producers from North America about (i) climate change in general, (ii) its current and future impacts on the health of sugar bushes and maple syrup yield, and (iii) their opinions about adaptation to climate change and their willingness to adopt different adaptation strategies. A total of 354 individuals completed the survey. The complete questionnaire (S1 File) as well as the complete dataset (S2 File) can be found in the Supplementary Material. Sixty-eight percent of the respondents were Canadian producers (N = 241), and 32% were Americans (N = 113) (Table 1). When considering the distribution of maple syrup farms in Canada and the U.S., our study slightly over-represents the opinions of Canadian producers–mainly those from the province of Quebec–and under-represents the views of U.S. producers (Table 1). In terms of average tap yield, Canadian respondents reported slightly lower yields than the country average, while yields reported by U.S. respondents were more similar to the country average (Table 1).

**Table 1:** Comparisons between the distribution of maple syrup farms in North America (regions correspond to Fig 1) and the mean yield per tap per region with the results of our survey.

Maple syrup regions	Distribution of maple syrup farms				Yield per tap (lbs) in 2016	
	Censuses [53,54]		Present survey		Censuses [54,55]	Present survey
**Canada**	**11,468**	**(58)**	**241**	**(68)**	**3.43**	**3.17 ± 1.18**
Atlantic	416	(2)	7	(2)	2.79	2.48 **±** 0.99
Quebec	7,863	(40)	206	(58)	3.47	3.16 **±** 1.18
Ontario	3,003	(15)	28	(8)	3.08	3.50 **±** 1.16
**U.S.**	**8,261**	**(42)**	**113**	**(32)**	**3.70**	**3.74 ± 1.43**
Northeast	4,646	(24)	63	(18)	3.90	3.71 **±** 1.39
Midwest	3,612	(18)	50	(14)	2.77	3.79 **±** 1.50
**Total**	**19,719**	**(100)**	**354**	**(100)**	**3.64**	**3.35 ± 1.29**

Most survey respondents were male (87.4%), the mean respondents’ age was 49.1 (±13.7), and 66.9% had at least a college degree. A quarter (24.6%) of the respondents described their political view as being on the left or center-left of the political spectrum, 40.6% on the center, and 34.8% on the center-right or right. The average number of tap per farm was 6442 (±13,180), the average yield was 3.35 (±1.29) pounds per tap, and 71.0% of the respondent reported using tubing with vacuum to harvest sap. Most respondents were small-scale producers, as the percentage of household income contributed by maple sugar business was higher than 50% for only 20.2% of the respondents (Section D in S1 File).

To verify if our sample was representative of the larger population of maple syrup producers, we compared some characteristics of the survey respondents with available data from census made in the province of Quebec by the Ministère de l’Agriculture, des Pêcheries et de l’Alimentation du Québec [56] and in the U.S. by the United States Department of Agriculture [57]. The percentage of women in our sample was slightly lower than the larger population of maple syrup producers, as was the average age of respondents (Table 2). In Quebec, fewer respondents declared to hold a post-secondary diploma (61%) compared to the larger population of maple syrup producers in that province (71%) [56]. In the U.S., fewer respondents declared that their maple syrup business contributed to at least 50% of their household income (9%) compared to the larger population of maple syrup producers in the U.S. (22%) [57].

**Table 2:** Comparisons between the percentage of women and mean age of maple syrup producers in Quebec and the U.S. with the results of our survey.

Maple syrup regions	Percentage of women		Mean age	
	Censuses [57,58]	Present survey	Censuses [56,57]	Present survey
Quebec	24.00	17.24	55.00	47.92
U.S.	9.78	5.36	55.30	50.81

Using conditional inference trees, we quantified the influence of socio-economic factors, characteristics of the sugar bush, and the spatial location of the sugar bush on responses given by survey participants. In the following sections, we present and discuss the main findings of our study and their consequences for implementing short- and long-term adaptation strategies in the maple syrup industry. The most influential variable(s) retained for each question or statement of the survey during data analyses can be found in Table 3.

**Table 3:** Variables selected as potential predictors for each question or statement of the survey.

No.	Questions or statements	*Age*	*Gen*	*Edu*	*Pol*	*Lat*	*Lon*	*Cou*	*Reg*	*Tap*	*Har*	*Yie*	*Map*	*Exp*	*Sto*	*Inc*	*Inv*
**A.**	**General perceptions of climate change**																
A.1.	How confident are you that the average temperature on Earth is increasing?				●												
A.2.	Is the Earth getting warmer mostly because of human activity such as burning fossil fuels or mostly because of natural patterns in the Earth’s environment?				●												
	*Indicate your degree of agreement for the following* *statements*																
A.3.	I have a good knowledge of climate change.																
A.4.	Climate change impacts are happening slowly enough to let us adapt as it comes.				●					○							○
A.5.	Climate change is now noticeable in my region.				●												
A.6.	The impact of climate change on me and my community is tangible.			●		○											
A.7.	The projected impacts of climate change are exaggerated.				●									○			
A.8.	*What do you think is the probability that the following climatic events will happen more frequently in the next 30 years*?																
A.8.1.	High annual mean temperature				●												
A.8.2.	Heavy rainfall episodes				●												
A.8.3.	Snow and ice storms				●												
A.8.4.	Droughts				●					○							
A.8.5.	Forest fires			○						○							
A.8.6.	Insect outbreaks	○		○	●		○			○						○	
A.8.7.	Warm winters	○		●	●					○						●	
A.8.8.	Shifts in timing of the spring period in which freeze-thaw events happen				●										○		
A.8.9.	High number of winter thaw events				●												
A.8.10.	Severe windstorms				●												
A.8.11.	Summer heatwaves			○		●			●			○					
A.8.12.	Hail storms				●	●	○		●								○
A.8.13.	Extreme high temperature periods during the spring season that prematurely stop or slow sap flow	○			●												
**B.**	**Climate change impact on maple syrup production**																
B.1.	*In the last decades*, *which of these climatic hazards have caused significant damage to your sugar bush*?																
B.1.1.	Ice storm					●			●					●			
B.1.2.	Hail					○	○			○							
B.1.3.	Drought					○				○						○	
B.1.4.	Windstorm and tornadoes				○	○			○								
B.1.5.	Forest fire															○	
B.1.6.	Insect outbreak															●	
B.1.7.	Invasive species					○				○						○	
B.2.	In the last decades, what have been the impact of climate change on tap yield?					●	○					○					
	*Indicate your level of agreement with the following statements concerning current impacts of climate change*:																
B.3.	Maple syrup production is closely linked to climate.										●					●	
B.4.	The beginning of the tapping season is already happening earlier because of climate change.	○									○						
B.5.	Climate change has led to variability in the beginning of the tap season between years.				●												
B.6.	It is now easy to determine the best moment to tap maples.					○			●								
B.7.	In my sugar bush, I have observed an increase in maple dieback because of climate change.				●			○		○							
B.8.	In the next 30 years, what impact will have climate change on tap yield?				●												
	*Indicate your level of agreement with the following statements concerning future impacts of climate change*:																
B.9.	In the next 30 years, the beginning of the sap collection season is going to happen earlier because of climate change.	○			●												
B.10.	In the next 30 years, climate change will lead to variability in the beginning of the sap collection season between years.				●												
B.11.	In the future it will be harder and harder to determine the best moment to tap maples.								○								
**C.**	**Adaptation strategies to climate change**																
	*Indicate your level of agreement with the following statements*:																
C.1.	The existing information on climate change impacts on maple syrup production is easily accessible.									○	○						
C.2.	Possible adaptations to climate change are numerous for the maple syrup industry.				○				●								
C.3.	New ways to adapt to climate change are needed in the maple syrup industry.					○	○							○			
C.4.	I have a wide knowledge of the newest tapping technologies (e.g., high-vacuum tubing, new spouts, liming and fertilizing, reverse osmosis, silvicultural management, tube cleaning/spout replacement).										●						
C.5.	New maple syrup technologies will help me face the new challenges coming from climate change.										●						
C.6.	It is highly probable that I will adopt climate adaptation strategies if I think it could increase my maple syrup production.									○	●						
C.7.	Before making any changes to adapt to climate change, I will wait to see what effects it has on my maple syrup production.				○	○	○	●									
C.8.	*Which of these adaptation measures would allow producers in general to effectively adapt to climate change*?																
C.8.1.	Increasing the sugar bush’s number of taps.					○									○		
C.8.2.	Installing a high-vacuum tubing system for sap collection.					○	●			○							
C.8.3.	Tapping earlier in the year.								●								
C.8.4.	Using spring forecast models of sap flow to predict the perfect moment to tap.												○				
C.8.5.	Using maples adapted to future climate conditions.			○													
C.8.6.	Doing silvicultural management in your sugar bush to, for example, maintain the density of trees at a good level or to favor biodiversity.									○	●			○			
C.8.7.	Liming and fertilizing to limit maples dieback.	○				○			●	○							
C.8.8.	Adopting strong sanitation practices (tubing and spout cleaning and/or annual spout replacement).					●			○								
C.8.9.	Tapping red maples.									●			●				
C.8.10.	Keeping track of new research about maple production.					○	○			○							
C.9.	*Which of these adaptation measures would you like to use yourself*?																
C.9.1.	Increasing the sugar bush’s number of taps.				○												
C.9.2.	Installing a high-vacuum tubing system for sap collection.										●						
C.9.3.	Tapping earlier in the year.										●						
C.9.4.	Using spring forecast models of sap flow to predict the perfect moment to tap.																
C.9.5.	Using maples adapted to future climate conditions.					●	○					○		○		○	
C.9.6.	Doing silvicultural management in your sugar bush to, for example, maintain the density of trees at a good level or to favor biodiversity.										●						
C.9.7.	Liming and fertilizing to limit maples dieback.					○			●								
C.9.8.	Adopting strong sanitation practices (tubing and spout cleaning and/or annual spout replacement).										●						
C.9.9.	Tapping red maples.				○	○	○			○	○	○	●				
C.9.10.	Keeping track of new research about maple production.						●				●						
C.10.	*Which of these adaptation measures would you like to see used by the maple syrup industry*?																
C.10.1.	Promoting the distinctive syrup harvested in the very late season.						●			○						●	
C.10.2.	Helping the northward progression of sugar maple by plantation and by human augmented seed transport.						○		○	○	○						
C.10.3.	Tapping in the north of the sugar maple distribution range.				●						○		○			○	○
C.10.4.	Selecting maples that are adapted to future climatic conditions.			○		○							○				
C.11.	*To understand how easily your business would be able to adapt (if needed) to any potential impacts of climate change in the future*, *please indicate if you agree or disagree with each statement below*.																
C.11.1.	If any changes in labor (number of workers, and/or hours worked) are needed due to climate change, my business could quickly get the help it needs to operate.					○					●						○
C.11.2.	If any changes in maple production technologies are needed due to climate change, my business could afford to quickly adopt the new technologies.									○		○					○
C.11.3.	If any severe damage to my sugar bush occurred due to climate change, my business could quickly change how it collects and/or obtains sap.			●													
C.12.	*Are the following constraints limiting your adoption of new technologies and strategies designed to reduce the impacts of climate change on your business*?																
C.12.1.	Lack of information							●									
C.12.2.	Lack of financial means	●															
C.12.3.	Lack of technical support											●					
C.12.4.	I don’t believe that climate change will have much impact on my syrup production			●	●												

*Age*: Age of the respondent; *Gen*: Gender of the respondent; *Edu*: Education level of the respondent; *Pol*: Political view of the respondent; *Lat*: Latitude; *Lon*: Longitude; *Cou*: Country; *Reg*: Maple syrup region; *Tap*: Number of taps; *Har*: Harvesting method; *Yie*: Yield in 2016; *Map*: Age of the largest maple; *Exp*: Number of years of experience; *Sto*: Someone to take over after retirement; *Inc*: Percentage of income from sugaring operations; *Inv*: Investment planning. Variable selection was based on variable importance (see text). Black dots (●) indicate significant predictors at *p* ˂ 0.05 retained in the construction of conditional inference classification trees. Unfilled dots (○) indicate variables selected during the first step of modelling but not retained in the final conditional inference classification tree.

### Part 1: General perceptions of climate change

Given the close links between climate and maple syrup production, and the close contacts between sugaring operations and the forest environment, we hypothesized that (i) maple syrup producers would be more sensitive to the concerns raised by the scientific community about climate change than the general population, and that (ii) their perceptions would be relatively independent of political view. Our results do not support these hypotheses. Instead, we found that surveyed Canadian maple syrup producers were a bit less confident that the average temperature on Earth is increasing (77.2%) than the general population of the Ontario, Quebec and Atlantic regions of Canada (81.1%) [84]. This difference might be related to the fact that most survey respondents were men (Table 2), as recent studies have shown that sex was an important predictor of climate change perception in the general population in the U.S. [85] and for cranberry growers in Massachusetts [86]. In the U.S., respondents were as likely to believe in global warming (70.4%) than the general population of the Midwest and Northeast regions (69.9%) in 2016 [87]. This is in agreement with a study on U.S. farmers that shown that 65% of them believe that climate change is a reality, which is fairly similar than the general public (67–73%) [88].

**Fig 2:**
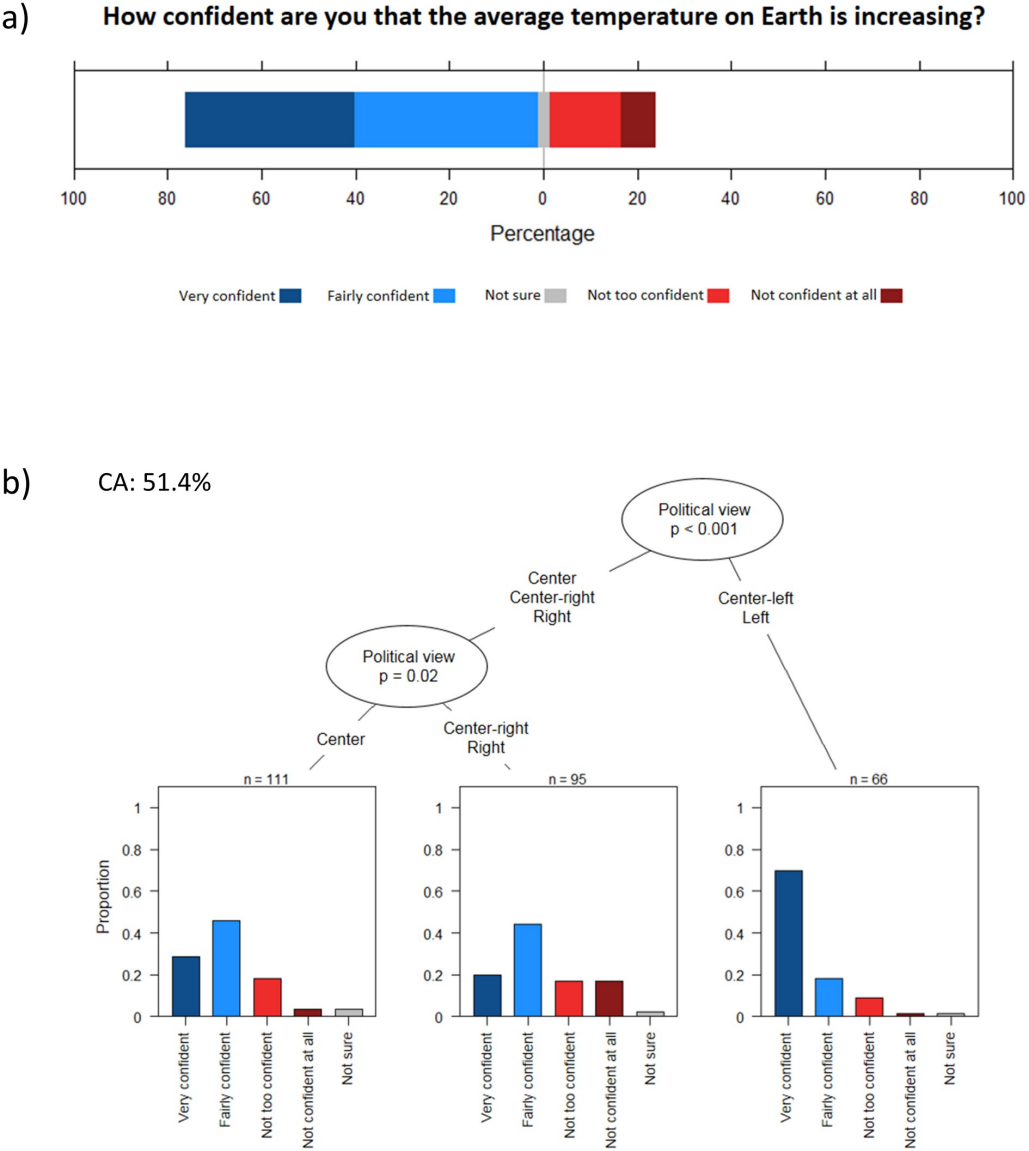
Belief in climate change. **a)** Responses of the survey participants to the question *How confident are you that the average temperature on Earth is increasing*? **b)** Conditional inference classification tree predicting the responses. Only significant predictors at p ˂ 0.05 were retained by the algorithm. Sample size used to build the tree can be calculated by adding sample sizes indicated at each terminal node. CA value is the classification accuracy.

Furthermore, as for the general population of Canada and the U.S. [40–42], political view was the strongest predictor of the responses for this statement (Table 3): while a majority (75%) of maple syrup producers were very or fairly confident that the average temperature on Earth is increasing (Fig 2a), this proportion was much higher (~ 90%) for respondents that positioned themselves at the left or center-left of the political spectrum (Fig 2b).

**Fig 3:**
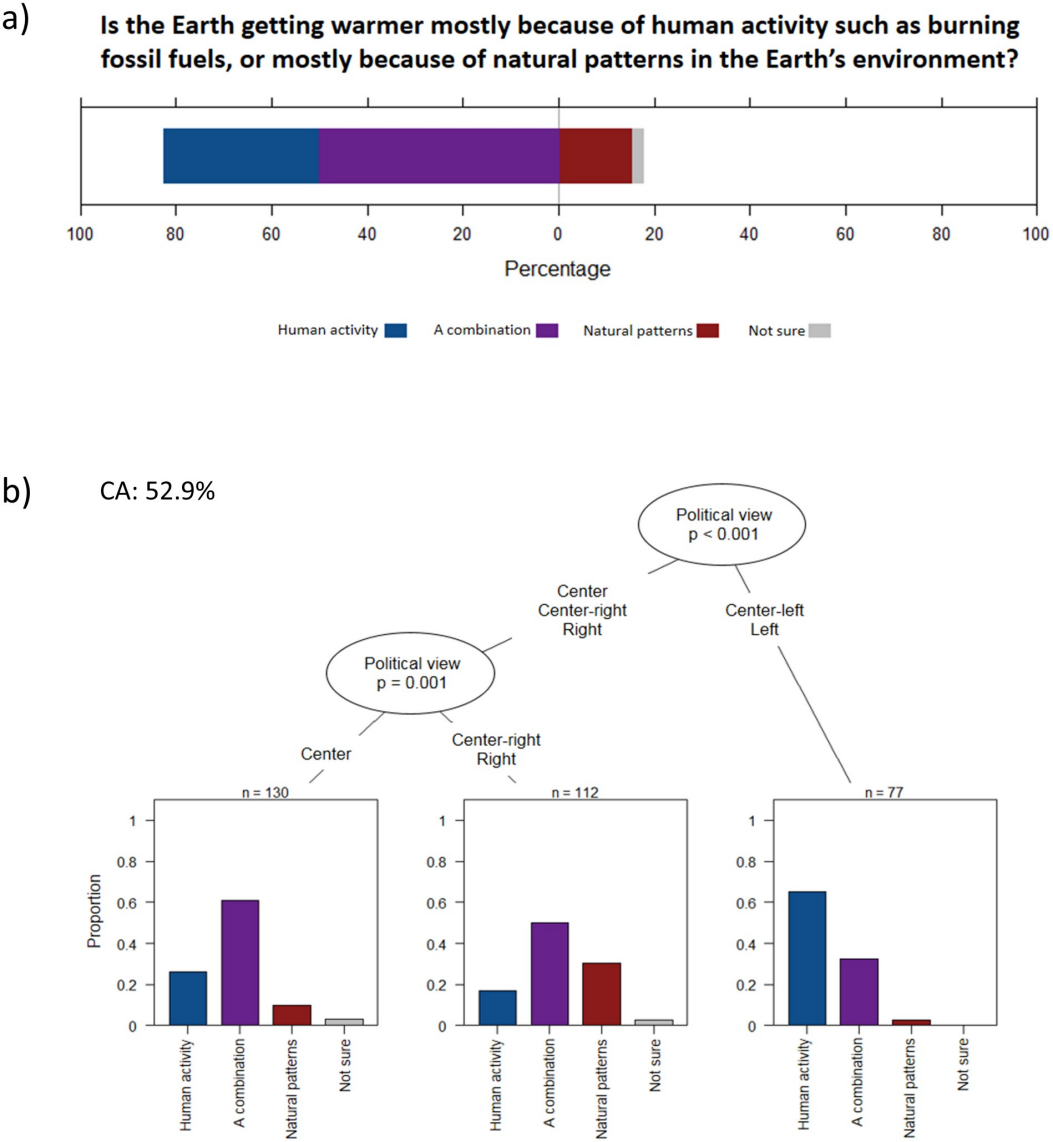
Causes of climate change. **a)** Responses of the survey participants for the question *Is the Earth getting warmer mostly because of human activity such as burning fossil fuels or mostly because of natural patterns in the Earth’s environment*? **b)** Conditional inference classification tree predicting the responses. Only significant predictors at p ˂ 0.05 were retained by the algorithm. Sample size used to build the tree can be calculated by adding sample sizes indicated at each terminal node. CA value is the classification accuracy.

Regarding the perceived causes of climate change, 32% of the respondents identified human activity as the main driver, 50% identified a combination between human activity and natural patterns, and 15% attributed the causes to natural patterns only (Fig 3a). Again, political view was the main predictor of the responses to this question, with respondents from the left and center-left being more inclined to identify human activity as the main driver of climate change (~ 65%) than other respondents (~ 20%) (Fig 3b).

**Fig 4:**
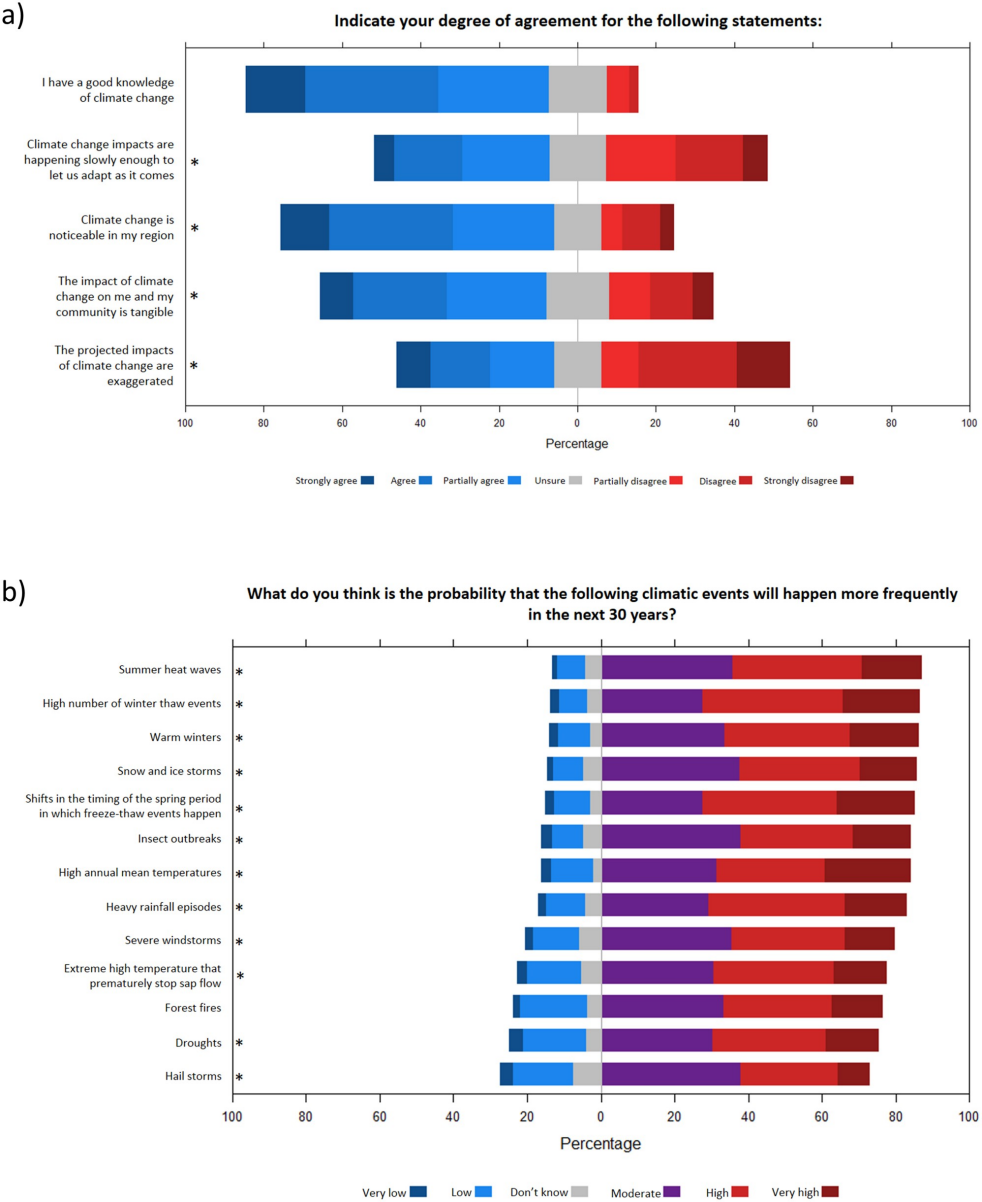
General impacts of climate change. **a)** Responses of the survey participants for general statements about climate change. **b)** Responses of the survey participants for the question *What do you think is the probability that the following climatic events will happen more frequently in the next 30 years*? Statements followed by a * symbol indicate a significant effect of at least one predictor (see Table 3 and S3 File).

For the perceived impacts of climate change (Fig 4a), political view was also the most important factor determining the responses of survey participants (Table 3). Respondents at the left of the political spectrum were less likely to agree that climate change impacts are happening slowly enough to let them adapt as the impacts appear (Fig A in S3 File), and that the projected impacts of climate change are exaggerated (Fig D in S3 File). Also, these respondents were more likely to agree that climate change is noticeable in their region (Fig B in S3 File). Education level significantly explained the responses of participants for the statement *The impact of climate change on me and my community is tangible*, with university-graduated respondents being more likely to agree (Fig C in S3 File).

Finally, regarding the probability that specific climatic events (e.g. high annual mean temperature, heavy rainfall episodes, etc.) would happen more frequently in the next 30 years (Fig 4b), political view was frequently a significant predictor (Table 3). For a majority of these questions, respondents at the left of the political spectrum were more inclined to believe that the probability of an increased frequency of extreme climatic events in the next 30 years was high or very high (Figs. F-P in S3 File). Other variables that significantly explained the responses of the survey participants for this subsection of the survey included educational level, latitude, region and percentage of household income contributed by the maple sugar business (Table 3; Figs. E, G, and P in S3 File).

### Part 2: Climate change impact on maple syrup production

Potential impacts of climate change on maple syrup production include changes in the exposure of the sugar maple stands to extreme climatic events [21], sap flow timing [24–26], and maple syrup yield [23]. With the northward shift of the climatic envelope favorable to the sugar maple [78], production in U.S. states in the southern portion of the maple’s range could be more at risk by the end of the century than in northern regions. For this reason, we expected a latitudinal gradient on producers’ opinions about the effects of climate change on sugar bush health and maple syrup production. Partly in line with this hypothesis, we found a few geographic variables had a significant effect on responses to some questions, but political view was also an important and significant factor shaping the responses, particularly with regard to future impacts (Table 3).

**Fig 5:**
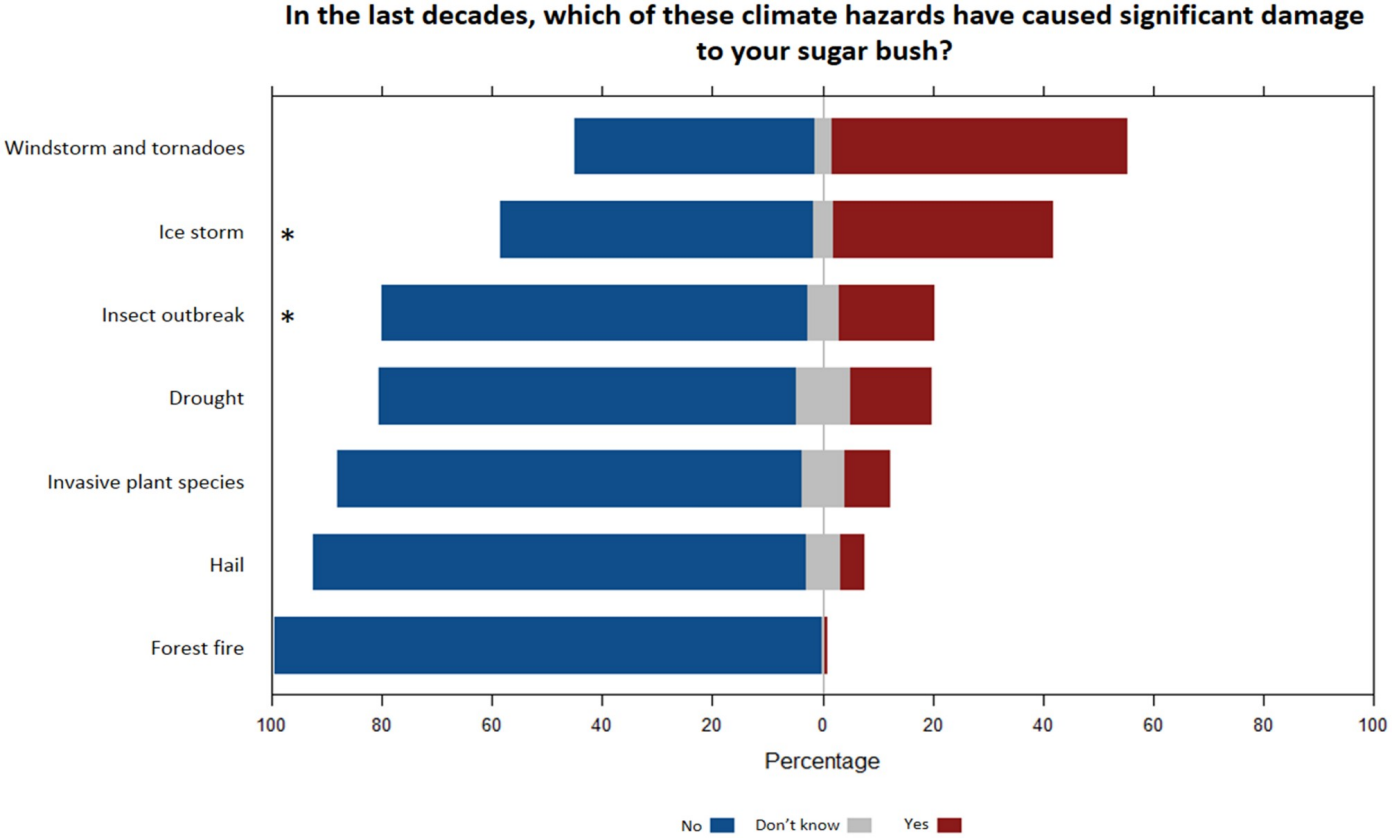
Exposure to past climatic events. Responses of the survey participants for the question *In the last decades*, *which of these climatic events have caused significant damages to your sugar bush*? Statements followed by a * symbol indicate a significant effect of at least one predictor (see Table 3 and Supplementary Material 3).

From a list of climatic hazards, windstorms and tornadoes were identified by the largest proportion of respondents as having caused significant damage to their sugar bush in the last decades (53%), followed by ice storms (40%), insect outbreaks (17%), droughts (14%), and invasive plant species (12%) (Fig 5). Significant models were obtained only for ice storms (Fig Q in S3 File) and insect outbreaks (Fig R in S3 File). Interestingly, respondents from southern Canada with more than 10 years of experience were more likely to report damages by ice storms to their sugar bushes during the last decades (Fig Q in S3 File). This pattern can be explained by the large areas of forest damaged by the ice storm of January 1998 that befell on the province of Quebec [89–91].

**Fig 6:**
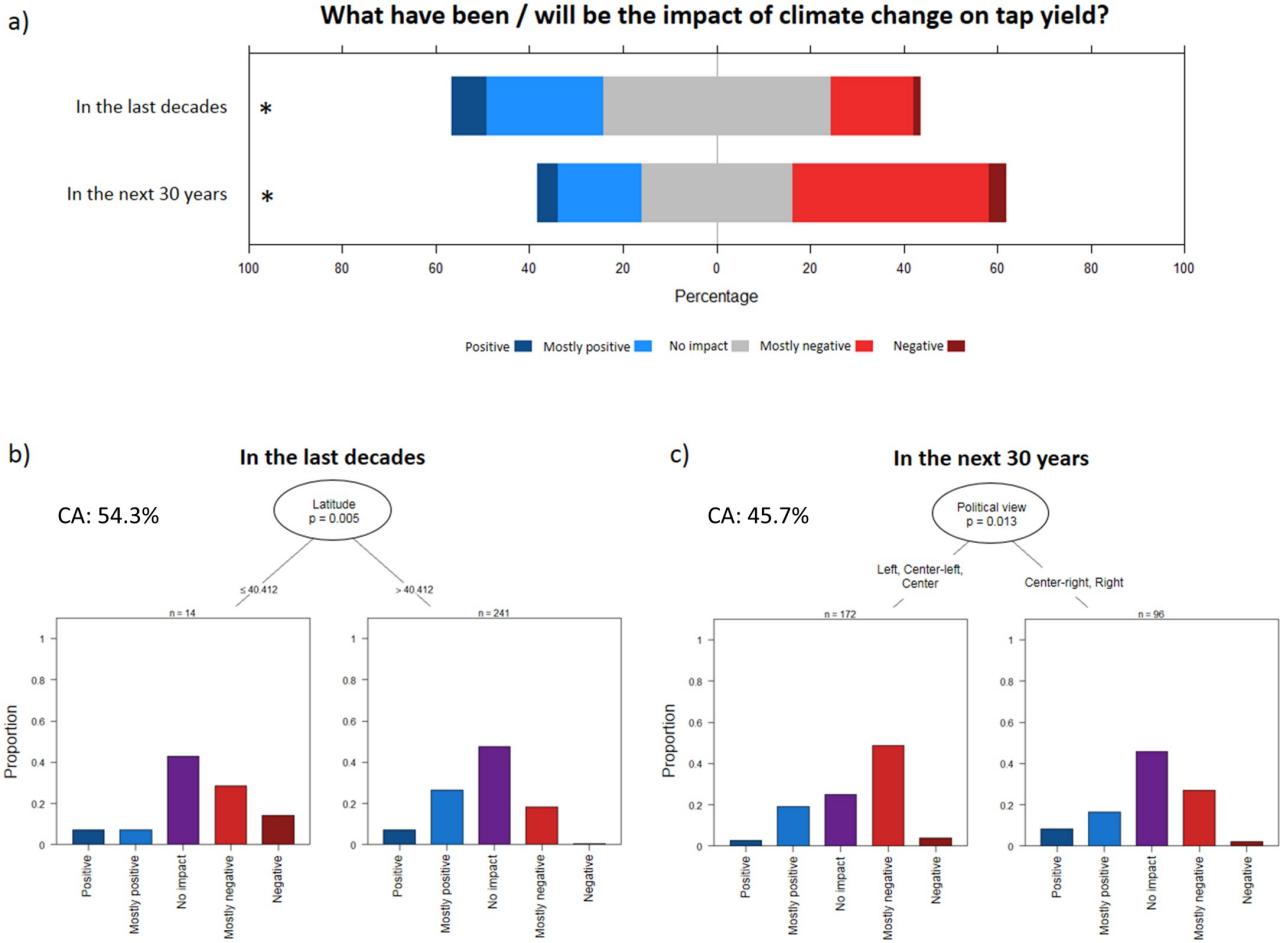
Climate change and tap yield. **a)** Responses of the survey participants for the questions *What have been/will be the impact of climate change on tap yield in the last decades and in the next 30 years*? **b and c)** Conditional inference classification trees predicting the responses. Only significant predictors at p ˂ 0.05 were retained by the algorithm. Sample size used to build the tree can be calculated by adding sample sizes indicated at each terminal node. CA value are the classification accuracies.

When asked for their perceptions of the impacts climate change has had on tap yield in recent decades, almost half (49%) of the respondents reported no impacts, 32% reported positive or mostly positive impacts, and 19% reported negative or mostly negative impacts (Fig 6a). However, the proportion of producers reporting negative impacts was significantly higher for respondents from lower latitudes (i.e. ≤ 40°) (Fig 6b). This result suggests that maple syrup production in the southern portion of the study area in the U.S. has already been impacted by global warming in recent years, potentially revealing the first manifestation of the expected northward shift of the climatic envelope favorable to sap flow [24]. For the next 30 years, a higher proportion (45%) of the respondents are anticipating negative or mostly negative impacts (Fig 6a), with respondents from the left to the center of the political spectrum being more likely to anticipate negative impacts (Fig 6c). These results are somewhat surprising since political view appears to be a stronger predictor of producers’ beliefs about future climate change impacts than personal experience due to past exposure to climate change effects. In other words, while maple syrup producers recognize impacts of climate change based on their personal experience, political ideology is more important in shaping their beliefs about future impacts. Interestingly, a similar result has been found among Idaho’ farmers: while many are noticing changes in precipitations, winter temperatures and growing season lengths, only a few connect these with anthropogenic climate change, indicating that direct experience of climate change manifestations is not sufficient to overcome the influence of political views [92].

**Fig 7:**
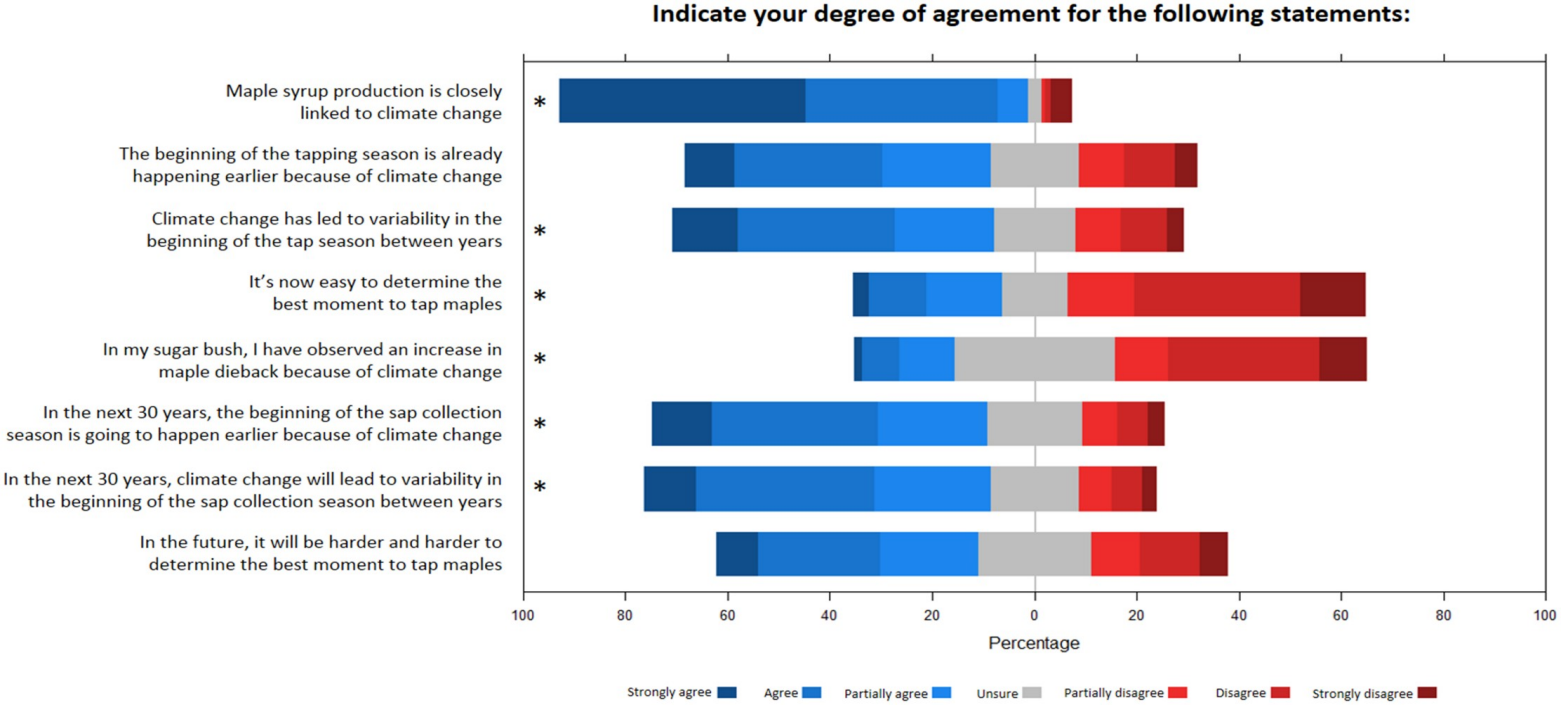
Climate change and maple syrup production. Responses of the survey participants for statements about climate change impacts on maple syrup production. Statements followed by a * symbol indicate a significant effect of at least one predictor (see Table 3 and S3 File).

A substantial majority (86%) of participants strongly agreed that maple syrup production is closely linked to climate, but only a minority observed an increase in maple dieback because of climate change (Fig 7). Regarding the beginning of the tapping season, 59% of producers answered that they have already observed its earlier occurrence because of climate change. A similar proportion of respondents agreed with the idea that this trend will continue in the future (Fig 7), but the proportion was significantly lower for respondents at the right end of the political spectrum (Fig W in S3 File). For statements concerning the variability in the beginning of the tapping season from year to years, 63% of the respondents agreed to a certain degree that climate change has led to higher variability, and 60% agreed that the trend will continue in the next 30 years (Fig 7). For these two statements, political view was the most important predictor of response, with participants from the right of the political spectrum having less agreement with the two statements (Figs. T and V in S3 File). Finally, only 29% of the respondents agreed with the statement that it is now easy to determine the best moment to tap maples (Fig 7), this proportion being lowest for respondents from U.S. regions (Fig U in S3 File). About half (51%) of the respondents agreed with the statement that in the future, it will be harder and harder to determine the best moment to tap maples (Fig 7); there was no difference between countries (Table 3).

### Part 3: Adaptation strategies to climate change

**Fig 8:**
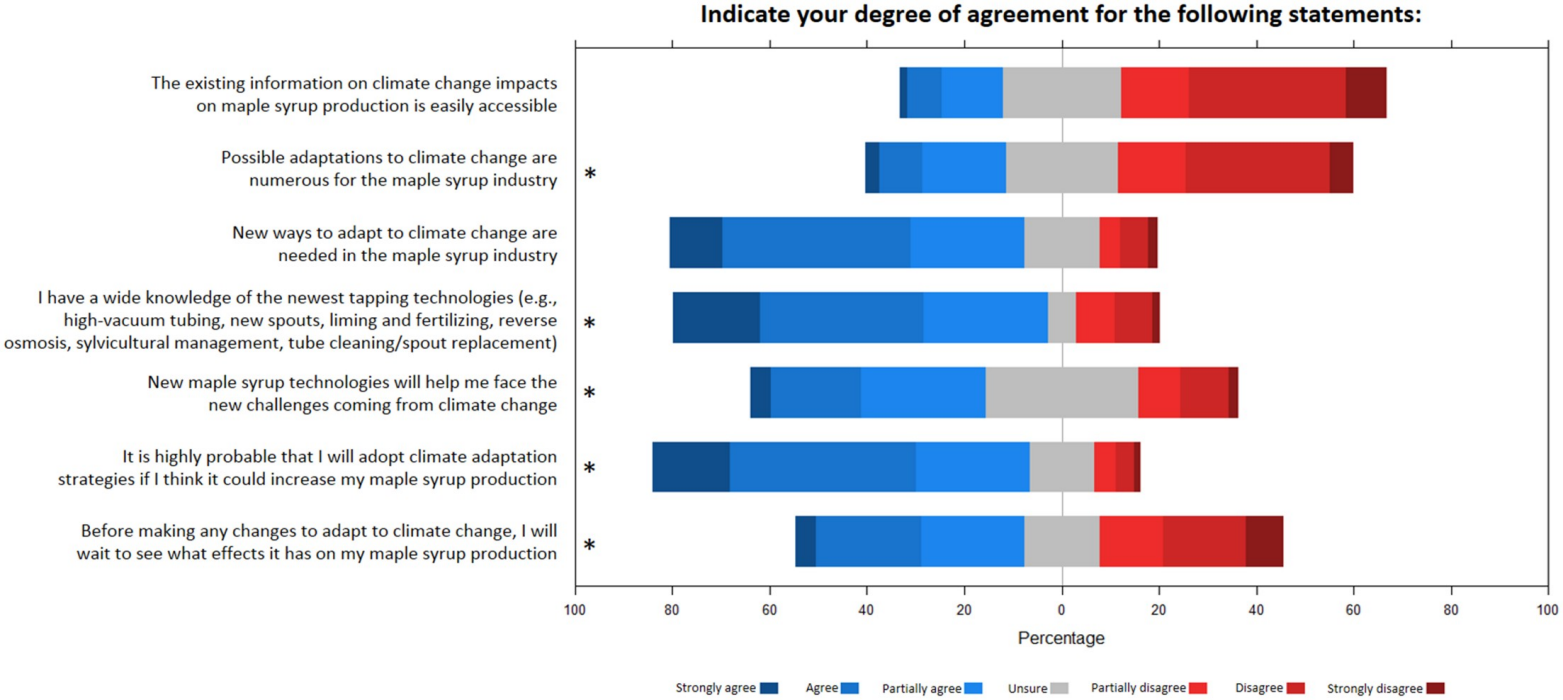
General statements on adaptation to climate change for maple syrup production. Responses of the survey participants for statements about adaptation to climate change for maple syrup production. Statements followed by a * symbol indicate a significant effect of at least one predictor (see Table 3 and S3 File).

Regarding questions and statements about adaptation to climate change, we hypothesized a significant influence of the scale of the sugaring operation on respondents’ responses, with large-scale producers being more inclined to adopt adaptation measures. We first proposed general statements on adaptation to climate change for maple syrup production (Fig 8). Sap harvesting method (i.e. buckets or bags, tubing with or without vacuum) is generally related to the size of sugaring operations, and was often selected as the stronger predictor of responses (Table 3). For example, producers using vacuum tubing to collect sap were more likely to agree with the statement that new ways to adapt to climate change are needed in the maple syrup industry, and that they will adopt climate adaptation strategies that will increase maple syrup production (Fig AB in S3 File). Also, respondents using tubing for sap collection were more likely to agree that new maple syrup technologies will help mitigate challenges related to climate change, and that they would probably adopt adaptation strategies if they will increase maple syrup production (Fig AA-AB in S3 File).

**Fig 9:**
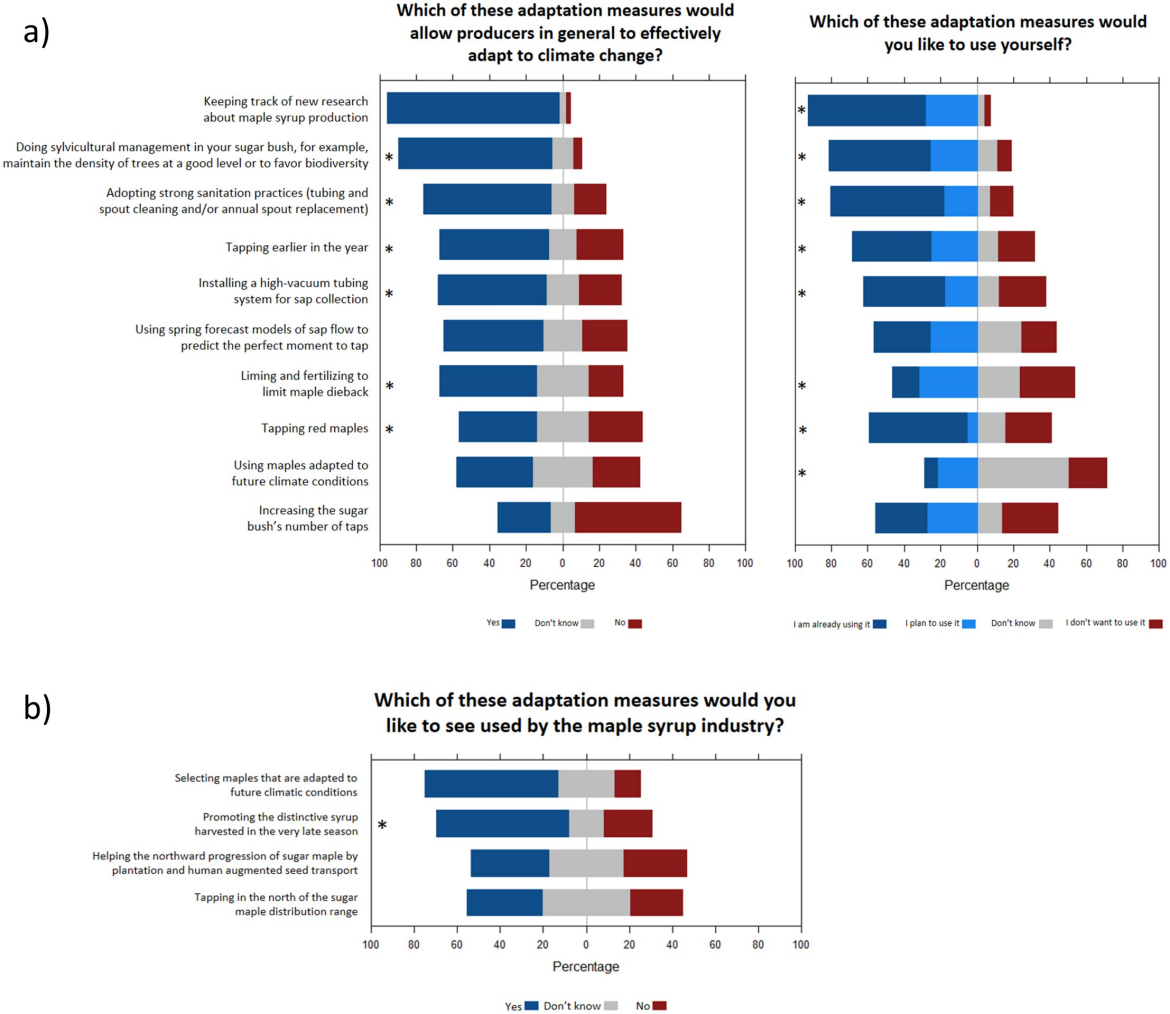
Specific adaptation measures. Responses of the survey participants for questions about specific adaptation measures that **a)** maple syrup producers or **b)** the maple syrup industry can adopt to mitigate climate change impacts. Statements followed by a * symbol indicate a significant effect of at least one predictor (see Table 3 and Supplementary Material 3).

Survey participants were then asked if various adaptation measures would effectively allow maple syrup producers to adapt to climate change, and if they would like to use these strategies in the future (Fig 9a; Table 4). The measures most frequently identified as efficient by respondents were keeping track of new research about maple syrup production, doing sylvicultural management, and adopting extensive spout and tubing sanitation practices (heavy use of sanitizers and/or annual replacement of spouts to lengthen taphole viability and enhance yield). Less popular measures included increasing a sugar bush’s number of taps, using maples adapted to future climate conditions, and tapping red maples (Fig 9a). When asked which of these adaptation measures they would like to use themselves, the perceptions of the respondents were generally in line with those of the previous question (Fig 9a). When asked which adaptation measures they would like to see developed by the maple syrup industry, however, the two positively viewed measures were selecting maples that are adapted to future climatic conditions, and promoting the distinctive syrup harvested in the very late season (Fig 9b).

**Table 4:** Description of the potential adaptation measures included in the survey, with corresponding findings from our survey study.

Level of implementation	Adaptation measure	Description	Findings from our study
**By maple syrup producers themselves**	Keeping track of new research about maple syrup production	The adaptation of the maple syrup industry to climate change depends in part on fundamental and applied research.	The overwhelming majority (92%) of respondents agreed that this measure is effective, and most respondents were already using it (65%) or planning to use it (28%) (Fig 10). Large-scale producers (i.e. those using tubing with vacuum to collect sap) were more likely to be already tracking new research about maple syrup production (Fig AJ in S3 File).
	Tapping earlier in the year	Early tapping could mitigate the negative effects of early sap flow seasons induced by climate change [6] and ensure that producers do not miss the start of the sap flow season. Tapping earlier has been shown to maintain maple syrup yields in both the short and long-term [59,60].	More than half (59%) of respondents identified this measure as effective, and this proportion was higher for respondents from Ontario and the two U.S. regions (Fig AF in S3 File). Forty three percent of the respondents said they were already using it, and this proportion reached 60% for larger-scale producers using high vacuum tubing (Fig AM in S3 File).
	Using spring forecast models of sap flow	There are no specific predictive models for determining the sap flow period of maples trees [61]. Only weather forecasts, intuition, experience and a good knowledge of maple syrup production allow producers to assess the best time to tap their sugar maples. Weather forecasts have improved considerably in the last three decades [62], and can be expected to become more reliable in the future.	Just over half of the respondents answered that seasonal forecast models of sap flow would allow them to adapt to climate change, and 26% answered that they were planning to use them in the future (Fig 10). It seems therefore that models more adapted to maple syrup production would be of interest for maple syrup producers.
	Liming and fertilizing to limit maple dieback	Liming has been shown to have a positive impact on maple trees growth when soil conditions are not optimal [63,64]. Also, nitrogen fertilization has been shown to increase sap sweetness [16]. To be effective, however, liming and fertilizing have to be integrated into a coherent sylvicultural strategy [65].	There is a significant contrast between the opinions of Canadian and U.S. maple syrup producers regarding this measure (Fig AH in S3 File). More than 60% of Canadian respondents believe that liming and fertilizer applications are solutions for climate change adaptation, while only 29% of Americans agree with this statement. This difference may be related to the current use of liming and fertilizers by producers, which varies considerably between the two countries (Fig AO in S3 File). This difference could be due to the fact that maple stands soils in the U.S. are generally richer and less acidic than in Canada [66].
	Doing sylvicultural management	In order to maintain a healthy maple stand, it can be desirable to diversify the species composition and age structure of the sugar maple bush and avoid the establishment of a pure maple stand [67]. Creating gaps and cutting moribund trees in the stand also helps promote the health and vigor of maple trees and the sustainability of maple syrup production.	A strong majority (84%) of respondents are of the view that forest management is a solution for climate change adaptation, and many answered that they were already using (55%) or were considering using this approach (26%) (Fig 10). Producers using high vacuum tubing were more likely to identify forest management as efficient (Fig AD in S3 File), or were already using it in a larger proportion (Fig AK in S3 File).
	Increasing the sugar bush’s number of taps	The number of taps in a sugar bush has a direct impact on yield. A higher number of taps in a sugar bush can increase total syrup production and thus compensate for a potential reduction in the length of the sap flow season.	Respondents considered increasing the number of taps the least effective adaptation measure to counter climate change impacts, though a majority answered that they had already increased of were planning to increase the number of taps (Fig 10).
	Tapping red maples	Red maple is a generalist species that tolerates widely variable climatic and soil conditions [68]. The abundance of this species considerably increased across North America during the 20^th^ century [69,70]. Although red maple provides lower sap sugar content than sugar maple [71], several producers tap them.	Forty-two percent of respondents answered that tapping red maple trees could help producers handle climate change impacts, and about half (53%) of respondents answered that they were already tapping red maples (Fig 10). However, only 5–6% of respondents are planning to tap this species in the future, and all of them are operating younger (≤60–80 years) sugar bushes (Fig AP in S3 File).
	Installing a high-vacuum tubing system for sap collection	In the past few years, yields per tap have tended to increase following the introduction of new sap harvesting technologies [11]. In particular, tubing with high vacuum can increase syrup production up to 3.5 times over a gravity system [72]. This improved yield could serve as a buffer for a shorter harvest season or for other negative impacts of climate change.	A majority (59%) of producers agreed that this measure could be efficient, and most producers were already using it or planning to use it (Fig 10). Producers from western regions were less likely to identify this measure as efficient (Fig AG in S3 File). Also, about 60% of small-scale producers using buckets or bags do not want to use high vacuum tubing in the future (Fig AN in S3 File).
	Strong sanitation practices	Cleaning maple syrup equipment (tubing, droplines, spouts) at the beginning and at the end of the harvest season reduces the growth of microorganisms in the sap and improves production during the entire sap flow season [73]. This measure could help prevent contamination of sap associated with higher temperatures.	A majority (70%) of respondents answered that this approach could address climate change issues (Fig 10). Interestingly, this proportion was higher (82%) for respondents from southern areas (Fig AE in S3 File). Producers using vacuum tubing were more likely to be using strong sanitation practices already, but a higher proportion of respondents using buckets or bags or tubing without vacuum were planning to use them in the future (Fig AL in S3 File).
**By the maple syrup industry**	Promoting the distinctive syrup harvested in the very late season	The harvest season usually ends with a week of sap flow that results in maple syrup with a taste very different from syrup produced during the rest of the season. This syrup does not meet the quality standards to be marketed by the industry. As a result, stocks of ‘buddy’ syrup accumulate year after year and are currently reaching millions of pounds [74]. Marketing this syrup as a sweetening agent [75,76] or for its nutraceutical properties [77] could allow for new market opportunities and increased revenues for the producers as the proportion of this type of syrup could potentially increase with a warming climate.	A majority (70%) of producers were of the opinion that the promotion of end-of-season syrup could help the maple industry adapt to climate change (Fig 10). This proportion was higher for larger-scale producers with higher percentage of total revenues that derive from their sugaring operations (Fig AR in S3 File).
	Tapping in the northern range of the sugar maple and helping the northward progression of the sugar maple	It is expected that the "climatic envelope" favorable to the growth of the sugar maple will shift northward with climate change [78]. However, the rate of sugar maple dispersal is not adequate to compensate for the displacement of its climatic envelope [79,80]. To circumvent this issue, assisted migration (that is to say, the transport and planting of seeds in new climatic zones; [81]) could act as a long-term measure of adaptation, enabling exploitation of new zones favourable for maple syrup production. First however, tapping efforts in the northern part of the current sugar maple range should be increased to see if maple syrup production can be profitable in northern areas.	Only 36% of respondents believed that tapping sugar maples in the northern part of its range might help the maple syrup industry adapt to climate change, and an almost identical number supports the idea of planting and transporting sugar maple seeds toward northern areas (Fig 10). It thus does not appear that a majority of producers support focusing on this avenue in dealing with climate change.
	Selecting maples that are adapted to future climatic conditions	Recent studies on tree genetics show that some genetic strains allow individuals of the same species to be better adapted to certain climatic conditions than others [82]. In this context, the search for sugar maple strains with attributes that promote survival in a warmer climate could act as a safeguard against the probable extirpation of the species from the southern part of its range [78]. The Sugar Maple Tree Improvement Program [83], conducted by Cornell University, undertook in the late 1990s the selection of genetic strains of sugar maple on the basis of maple syrup yield. However, since maple sap cannot be harvested from trees under 40 years of age, this type of project is inevitably a long-term one.	A majority (62%) of respondents were in favor of selection initiatives that might be developed by the maple syrup industry (Fig 10).

Producers using high vacuum tubing systems (>20 Hg) were more likely to identify sylvicultural management as efficient to adapt to climate change (Fig AD in S3 File) and were already using sylvicultural management in a larger proportion (Fig AK in S3 File). In addition, they were more likely to already be using strong sanitation practices already (Fig AL in S3 File). Producers using high vacuum tubing were also more likely to be tapping earlier in the year (Fig AM in S3 File), more likely to say they have a wide knowledge of newest tapping technologies (Fig Z in S3 File), and to already be keeping track of new research about maple syrup production (Fig AJ in S3 File). Finally, respondents using high vacuum tubing were more likely to agree that their business could quickly change its labor organisation (number of workers, and/or hours worked) following climate change impacts (Fig AS in S3 File).

The geographic location of the sugar bush was another important predictor of response for questions and statements about adaptation (Table 3). Only 23% of respondents from Canadian regions agreed with the statement that there are many ways to adapt to climate change for maple syrup production, compared to 43% of respondents from U.S. regions (Fig Y in S3 File). Respondents from these warmer regions within the study area were also more likely to identify the adoption of strong sanitation practices and earlier tapping as effective adaptation measures to mitigate climate change impacts (Fig AE-AF in S3 File), but less likely to identify liming and fertilizing as effective (Fig AH and AO in S3 File). There is, however, a considerable contrast between the producers of the two countries regarding the best moment to implement adaptation measures: the majority of U.S. producers (67%) believe that it is necessary to wait till they observe the effects of climate change, while Canadian producers are much more divided on this issue (Fig AC in S3 File).

**Fig 10:**
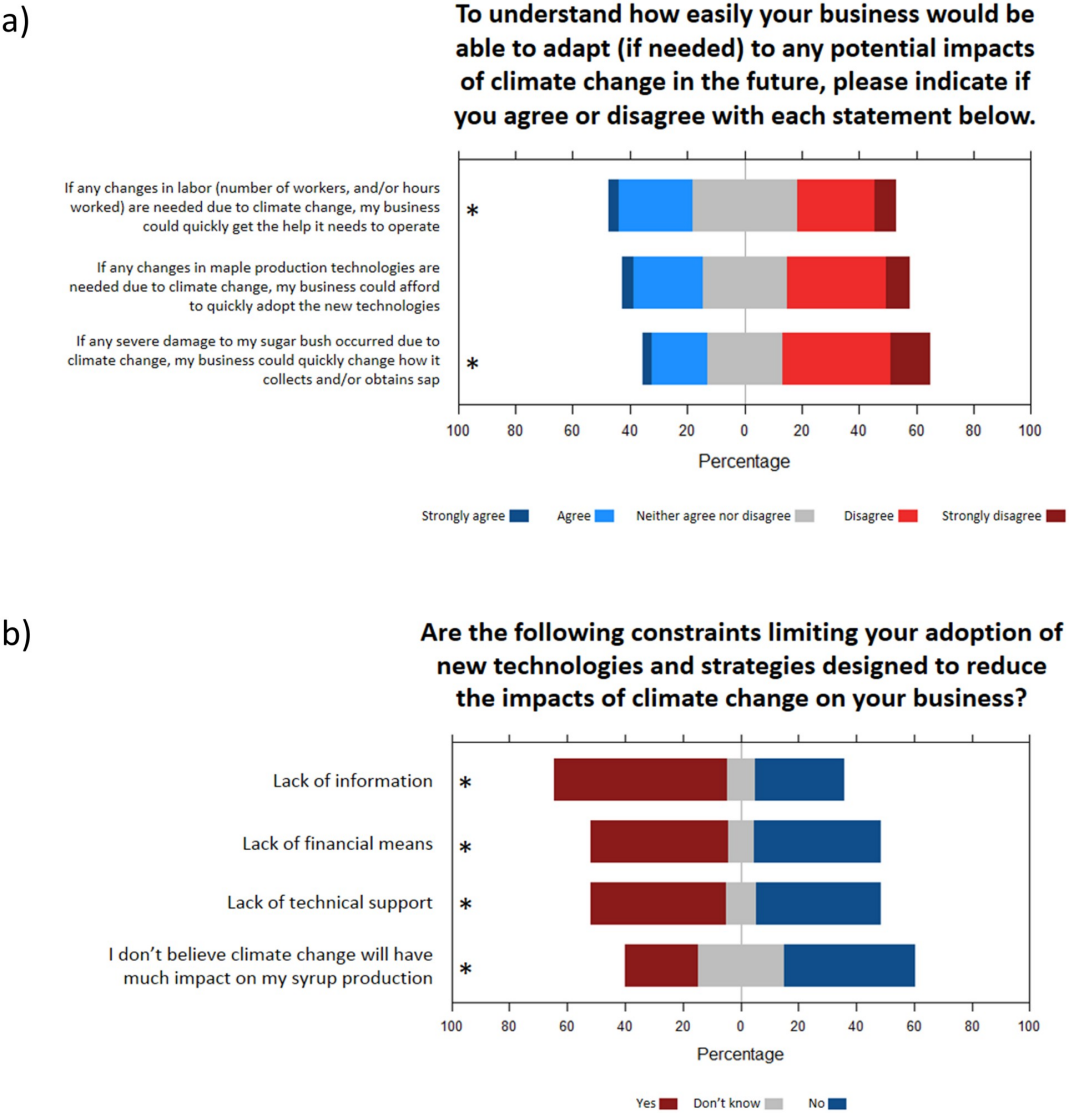
Adaptability and constraints to adaptation. **12.** Responses of the survey participants for questions about **a)** adaptability and **b)** potential barriers to adaptation. Statements followed by a * symbol indicate a significant effect of at least one predictor (see Table 3 and S3 File).

The last segment of the third part of the survey focused on the adaptability of respondents’ sugar bush, and barriers to adapting to climate change. Only a minority of respondents agreed that their business could quickly adapt to climate change impacts experienced through adjustments of labor, production technologies, or how sap is collected (Fig 10a). Regarding constraints to adaptation through the use of new technologies, lack of financial means was the most frequently identified (Fig 10b), particularly for younger respondents (Fig AV in S3 File). This constraint was followed in importance by the lack of information, particularly for Canadian respondents (Fig AU in S3 File), and the lack of technical support, particularly for respondents with lower sugar bush yields (Fig AV in S3 File). Finally, 25% of respondents identified their lack of belief in climate change as a constraint to adopting new technologies for coping with the challenges of climate change. This proportion was higher (almost 50%) for respondents at the right or center-right of the political spectrum, and respondents without high school diplomas (Fig AX in S3 File).

A more detailed discussion of findings associated with specific adaptation measures can be found in Table 4.

## Conclusions

In this study, we used a web survey to examine the perceptions of maple syrup producers from Canada and the U.S. about climate change in general, its effects on sugar bush health and productivity, and adaptation to climate change.

The first part of the survey focused on general perceptions of climate change. Overall, we found that a majority of respondents are confident that the average temperature on Earth is increasing. These levels of belief in climate change were somewhat similar than that of the general populations of Canada and the U.S. living in the areas covered by our survey [84,87]. As for the general population of both countries [40–42], political view was a very strong predictor of respondents’ general beliefs in climate change.

For the second part of the survey, which focused on current and future impacts of climate change on maple syrup production, our analyses retained a more diverse set of predictors, but political view was still the most frequent. One of the results indicate that almost half of respondents expect climate change to negatively impact tap yield in the next 30 years.

For the third part of the survey, which focused on adaptation strategies to climate change, influential predictors were most frequently associated with geography (e.g. latitude, region), and the scale of the sugaring operations (e.g. harvesting method, number of taps). Interestingly, a very large majority of respondents would adopt adaptation strategies if they believed it would increase maple syrup production. These include forest management to keep sugar bushes healthy and resilient to climatic hazards, investments in new technologies to boost productivity, and new varieties of resilient and productive sugar maple trees. However, the most important barriers to the implementation of these strategies appears to be the lack of easily accessible information about the effects of climate change on maple syrup production, followed by the lack of financial means and technical support.

Finally, about a quarter of respondents identified their lack of belief in climate change impacts on maple syrup production as limiting their adoption of adaptation measures. As increased in exposure to extreme climatic events can raise awareness and influence climate change beliefs [38,93], these respondents may change their opinions in the future. In the meantime, we believe that continuing to study the effects of climate change on maple syrup production and improving the ways of transmitting new information to maple syrup producers will help prepare the maple syrup industry for the climate of the future.

## Data limitations and future directions

In addition to the possible biases identified in Tables 1 and 2, it is important to mention that our sample may not be representative of all North American producers. Indeed, our analysis focuses mainly on producers that accepted to answer the survey. As a result, these producers may be more engaged about climate change, and may have access to more information than those who did not respond.

As our results suggest, the factors determining the opinions of maple syrup producers in the face of climate change are many and varied. Consequently, the adaptation of the maple industry to climate change will probably need a variety of strategies, each adapted to the climatic and socio-cultural specificities of each regions.

## Supporting information

S1 File Survey questionnaire. Survey questionnaire in the format presented to participants, with total responses indicated for each question.
(PDF)

S2 File Full dataset. Excel file containing the full dataset used for analyses. Column numbers correspond to Table 3 and S1 File. The geographic locations of the sugar bushes (i.e. latitude and longitude coordinates) have been removed from the table to ensure the anonymity of the respondents.
(XLSX)

S3 File Conditional inference (CI) classification trees. Selected conditional inference classification tree predicting the response rates of survey questions using only predictors identified as important during the random forest variable importance evaluation step of the analyses (see Table 1). For each tree, only significant predictors at p ˂ 0.05 were retained by the algorithm. Sample size used to build each tree can be calculated by adding sample sizes indicated at each terminal node. The classification accuracy was calculated for each tree using a subset of 20% the dataset.
(PDF)
