# Supplementary material for: Perceptions of U.S. and Canadian maple syrup producers toward climate change, its impacts, and potential adaptation measures
Source: PLoS One. 2019 Apr 25;14(4):e0215511. doi: 10.1371/journal.pone.0215511 (PMC6483340; doi:10.1371/journal.pone.0215511)
Supplement: S1 File — Survey questionnaire in the format presented to participants, with total responses indicated for each question. (PDF) [file pone.0215511.s001.pdf]

Supplementary Material S1

Survey questionnaire in the format presented to participants, with total responses indicated for each question.

Section A. General perceptions of climate change

A.1. How confident are you that the average temperature on Earth is increasing?

Very confident	Fairly confident	Not too confident	Not confident at all	Not sure	**Total answered**	No answer
127	138	54	25	9	**353**	1

A.2. Is the Earth getting warmer mostly because of human activity such as burning fossil fuels, or mostly because of natural patterns in the Earth's environment?

Human activity	A combination	Natural patterns	Not sure	**Total answered**	No answer
106	166	51	7	**330**	24

Indicate your degree of agreement for the following statements :	Strongly disagree	Disagree	Partially disagree	Unsure	Partially agree	Agree	Strongly agree	**Total answered**	No answer
**A.3** I have a good knowledge of climate change.	0	7	19	50	93	112	50	**334**	20
**A.4** Climate change impacts are happening slowly enough to let us adapt as it comes.	20	57	59	49	74	58	16	**333**	21
**A.5** Climate change is now noticeable in my region.	11	32	18	40	86	105	40	**332**	22
**A.6** The impact of climate change on me and my community is tangible.	17	36	34	54	84	79	27	**331**	23
**A.7** The projected impacts of climate change are exaggerated.	44	83	31	41	54	50	28	**331**	23

**A.8** What do you think is the probability that the following climatic events will happen more frequently **in the next 30 years**?	Very low	Low	Moderate	High	Very high	Don't know	**Total answered**	No answer
**A.8.1.** High annual mean temperatures	8	38	105	97	77	8	**333**	21
**A.8.2.** Heavy rainfall episodes	7	35	97	123	56	15	**333**	21
**A.8.3.** Snow and ice storms	4	27	125	109	51	17	**333**	21
**A.8.4.** Droughts	12	57	101	102	47	14	**333**	21
**A.8.5.** Forest fires	5	61	111	98	45	13	**333**	21
**A.8.6.** Insect outbreaks	9	28	126	102	51	17	**333**	21
**A.8.7.** Warm winters	7	29	111	113	61	10	**331**	23
**A.8.8.** Shifts in timing of the spring period in which freeze-thaw events happen.	7	33	92	121	70	10	**333**	21
**A.8.9.** High number of winter thaw events	7	25	91	126	69	13	**331**	23
**A.8.10.** Severe windstorms	6	42	118	102	45	20	**333**	21
**A.8.11.** Summer heatwaves	4	25	119	117	53	15	**333**	21
**A.8.12.** Hail storms	10	54	124	86	28	25	**327**	27
**A.8.13.** Extreme high temperature periods during the spring season that prematurely stop or slow sap flows	8	49	101	108	47	18	**331**	23

Section B. Climate change impact on maple syrup production

**B.1 In the last decades**, which of these climate hazards have caused significant damage to your sugar bush?	Yes	No	Don't know	**Total answered**	No answer
**B.1.1.** Ice storm	139	198	13	**350**	4
**B.1.2.** Hail	15	310	22	**347**	7
**B.1.3.** Drought	50	263	36	**349**	5
**B.1.4.** Windstorm and tornadoes	187	151	12	**350**	4
**B.1.5.** Forest fire	1	342	3	**346**	8
**B.1.6.** Insect outbreak	59	267	21	**347**	7
**B.1.7. Invasive plant species**	**27**	**280**	**27**	**349**	**5**

B.2 In the last decades, what have been the impact of climate change on tap yield?

Positive	Mostly positive	No Impact	Mostly negative	Negative	**Total answered**	No answer
25	87	170	61	5	**348**	6

Indicate your level of agreement with the following statements concerning **current impacts of climate change**:	Strongly disagree	Disagree	Partially disagree	Unsure	Partially agree	Agree	Strongly agree	**Total answered**	No answer
**B.3** Maple syrup production is closely linked to climate.	14	4	2	10	21	132	169	**352**	2
**B.4** The beginning of the tapping season is already happening earlier because of climate change.	15	35	31	61	75	102	33	**352**	2
**B.5** Climate change has led to variability in the beginning of the tap season between years.	11	32	31	56	68	108	44	**350**	4
**B.6** It's now easy to determine the best moment to tap maples.	44	114	46	46	52	40	10	**352**	2
**B.7** In my sugar bush, I have observed an increase in maple dieback because of climate change.	31	104	36	111	38	25	5	**350**	4

**B.8 In the next 30 years**, what impact will have climate change on tap yield?						
Positive	Mostly positive	No Impact	Mostly negative	Negative	**Total answered**	No answer
14	62	112	145	12	**345**	9

Indicate your level of agreement with the following statements concerning future impacts of climate change:	Strongly disagree	Disagree	Partially disagree	Unsure	Partially agree	Agree	Strongly agree	**Total answered**	No answer
**B.9 In the next 30 years**, the beginning of the sap collection season is going to happen earlier because of climate change.	11	21	24	65	75	114	40	**350**	4
**B.10 In the next 30 years**, climate change will lead to variability in the beginning of the sap collection season between years.	9	21	23	61	80	123	35	**352**	2
**B.11 In the future,** it will be harder and harder to determine the best moment to tap maples.	19	41	33	79	67	84	28	**352**	2

Section C. Adaptation strategies to climate change

Indicate your level of agreement with the following statements:	Strongly disagree	Disagree	Partially disagree	Unsure	Partially agree	Agree	Strongly agree	**Total answered**	No answer
**C.1** The existing information on climate change impacts on maple syrup production is easily accessible.	29	113	49	86	44	25	5	**351**	3
**C.2** Possible adaptations to climate change are numerous for the maple syrup industry.	16	104	49	81	61	31	9	**351**	3
**C.3** New ways to adapt to climate change are needed in the maple syrup industry.	6	20	15	55	82	135	37	**350**	4
**C.4** I have a wide knowledge of the newest tapping technologies (e.g., high-vacuum tubing, new spouts, liming and fertilizing, reverse osmosis, silvicultural management, tube cleaning/spout replacement).	5	27	28	21	90	118	62	**351**	3
**C.5** New maple syrup technologies will help me face the new challenges coming from climate change.	6	35	30	111	90	65	14	**351**	3
**C.6** It is highly probable that I will adopt climate adaptation strategies if I think it could increase my maple syrup production.	4	13	15	48	82	134	55	**351**	3
**C.7** Before making any changes to adapt to climate change, I will wait to see what effects it has on my maple syrup production.	26	59	46	54	74	76	14	**349**	5

	**C.8** Which of these adaptation measures would allow **producers in general** to effectively adapt to climate change?					**C.9** Which of these adaptation measures would **you** like to use yourself?					
	Yes	No	Don't know	**Total answered**	No answer	I am already using it	I plan to use it	I don't want to use it	Don't know	**Total answered**	No answer
**1.** Increasing the sugar bush's number of taps.	100	201	48	**349**	5	98	97	107	48	**350**	4
**2.** Installing a high-vacuum tubing system for sap collection.	207	81	62	**350**	4	157	62	90	42	**351**	3
**3.** Tapping earlier in the year.	207	87	54	**348**	6	148	88	68	41	**345**	9
**4.** Using spring forecast models of sap flow to predict the perfect moment to tap.	190	85	75	**350**	4	106	89	66	84	**345**	9
**5.** Using maples adapted to future climate conditions.	145	90	114	**349**	5	25	76	72	176	**349**	5
**6.** Doing silvicultural management in your sugar bush to, for example, maintain the density of trees at a good level or to favor biodiversity.	293	15	41	**349**	5	194	91	26	40	**351**	3
**7.** Liming and fertilizing to limit maples dieback.	184	65	100	**349**	5	51	112	106	82	**351**	3
**8.** Adopting strong sanitation practices (tubing and spout cleaning and/or annual spout replacement).	244	61	44	**349**	5	217	64	44	25	**350**	4
**9.** Tapping red maples.	148	103	99	**350**	4	188	20	88	55	**351**	3
**10.** Keeping track of new research about maple production.	329	8	13	**350**	4	226	99	11	14	**350**	4

**C.10** Which of these adaptation measures would you like to see used by **the maple syrup industry**?	Yes	No	Don't know	**Total answered**	No answer
**C.10.1.** Promoting the distinctive syrup harvested in the very late season.	214	78	57	**349**	5
**C.10.2.** Helping the northward progression of sugar maple by plantation and by human augmented seed transport.	125	102	121	**348**	6
**C.10.3.** Tapping in the north of the sugar maple distribution range.	121	83	143	**347**	7
**C.10.4.** Selecting maples that are adapted to future climatic conditions.	216	41	92	**349**	5

**C.11** To understand how easily **your business** would be able to adapt (if needed) to any potential impacts of climate change **in the future**, please indicate if you agree or disagree with each statement below.	Strongly disagree	Disagree	Neither agree nor disagree	Agree	Strongly agree	**Total answered**	No answer
**C.11.1.** If any changes in **labor** (number of workers, and/or hours worked) are needed due to climate change, my business could quickly get the help it needs to operate.	26	93	130	89	11	**349**	5
**C.11.2.** If any changes in maple production **technologies** are needed due to climate change, my business could afford to quickly adopt the new technologies.	28	120	104	85	12	**349**	5
**C.11.3.** If any **severe damage to my sugar bush** occurred due to climate change, my business could quickly change how it collects and/or obtains sap.	47	131	93	68	9	**348**	6

**C.12.** Are the following constraints limiting your adoption of new technologies and strategies designed to reduce the impacts of climate change on your business?	Yes	No	Don't know	**Total answered**	No answer
**C.12.1.** Lack of information	165	152	32	**349**	5
**C.12.2.** Lack of financial means	208	106	35	**349**	5
**C.12.3.** Lack of technical support	163	150	37	**350**	4
**C.12.4.** I don't believe that climate change will have much impact on my syrup production	87	157	105	**349**	5

Section D. Sugar bush characteristics

D.1 Where is your sugarbush?			
**Country**	**Maple syrup region**	**Province or State**	**N**
**Canada**<br>**Total answered: 241**	**Atlantic**<br>Total answered: 7	Nova Scotia	1
		New Brunswick	6
	**Ontario**	Ontario	28
	**Quebec**	Quebec	206
**U.S.**<br>**Total answered: 113**	**Northeast**<br>Total answered: 63	Connecticut	1
		Maine	12
		Massachusetts	4
		New Hampshire	4
		New Jersey	1
		New York	22
		Pennsylvania	4
		Vermont	15
	**Midwest**<br>Total answered: 50	Illinois	5
		Indiana	3
		Kentucky	2
		Michigan	3
		Minnesota	6
		Missouri	1
		Ohio	12
		Oklahoma	1
		Virginia	1
		Wisconsin	16

**D.2** How many taps did you collect sap from in your sugar bush in 2016?
$\bar{x} \pm SD = 6442 \pm 13180$<br>**Total answered: 354**

D.3 In 2016, what is the approximate age of the largest maples in your sugar bush?

20 - 40	40 - 60	60 - 80	80 - 100	100 - 120	120 +	Don't know	**Total answered**	No answer
6	37	79	62	49	97	18	**348**	6

**D.4** What harvesting method do you use in your sugar bush?					
Buckets or bags	Tubing without vacuum	Tubing with vacuum < 20 Hg	Tubing with vacuum > 20 Hg	**Total answered**	No answer
64	37	75	172	**348**	6

**D.5** How many years of experience do you have in the maple industry?										
0 - 5	6 - 10	11 - 15	16 - 20	21 - 25	26 - 30	31 - 35	36 - 40	41 +	**Total answered**	No answer
69	67	42	43	28	24	19	18	39	**349**	5

**D.6** Do you have someone to take over your sugar bush once you get retired?			
Yes	No	**Total answered**	No answer
174	171	**345**	9

**D.7** How much (%) of your household income is contributed by your maple sugar business?											
0 - 10%	11 - 20%	21 - 30%	31 - 40%	41 - 50%	51 - 60%	61 - 70%	71 - 80%	81 - 90%	91 - 100%	**Total answered**	No answer
155	46	44	16	16	15	9	11	5	30	**347**	7

**D.8** What was your mean syrup yield for the 2016 season (lbs/tap)(Gallons/tap)?								
(0 - 1) (0 - 0.075)	(1 - 2) (0.075 - 0.15)	(2 - 3) (0.15 - 0.225)	(3 - 4) (0.225 - 0.3)	(4 - 5) (0.3 - 0.375)	(5 - 6) (0.375 - 0.45)	(6+) (0.45+)	**Total answered**	No answer
47	80	80	106	48	24	17	**322**	32

**D.9** During the next 10 years, how much **($/tap)** do you plan to invest in **silvicultural work** (stand management, liming and fertilizing…) in your sugar bush?
$\bar{x}$ ± SD = 4.37 ± 11.36<br>**Total answered: 353**

**D.10** During the next 10 years, how much **($/tap)** do you plan to invest to **upgrade or replace equipment** in your sugar bush and sugarhouse (Ex: tubing, inverted osmosis, high-vacuum system, GPS, buckets…)?
$\bar{x}$ ± SD = 20.36 ± 133.54<br>**Total answered: 354**

**D.11** How much **($/tap)** do you plan to invest to **modernize the infrastructures** of your sugar bush during the next 10 years (Ex: restaurant, warehouse, off-road machinery, carriage, forest roads…)?
$\bar{x}$ ± SD = 10.52 ± 55.57<br>**Total answered: 353**

Section E. General characteristics

**E.1** What is your gender?				
Male	Female	Don't want to answer	**Total answered**	No answer
306	44	4	**354**	0

**E.2** What is your year of birth? (Converted to age of respondents in 2016)
$\bar{x} \pm SD = 49.05 \pm 13.69$<br>**Total answered: 354**

**E.3** What is your education level?					
No diploma	High school	College Degree	Post-graduate degree	**Total answered**	No answer
16	101	151	85	**353**	1

**E.4** How would you describe your political views on a left to right scale, where left is liberal and right is conservative?						
Left	Center left	Center	Center right	Right	**Total answered**	No answer
24	60	139	80	39	**342**	12
