# Supplementary material for: Perceptions of U.S. and Canadian maple syrup producers toward climate change, its impacts, and potential adaptation measures
Source: PLoS One. 2019 Apr 25;14(4):e0215511. doi: 10.1371/journal.pone.0215511 (PMC6483340; doi:10.1371/journal.pone.0215511)
Supplement: S3 File — Selected conditional inference classification tree predicting the response rates of survey questions using only predictors identified as important during the random forest variable importance evaluation step of the analyses (see Table 1). For each tree, only significant predictors at p ˂ 0.05 were retained by the algorithm. Sample size used to build each tree can be calculated by adding sample sizes indicated at each terminal node. The classification accuracy was calculated for each tree using a subset of 20% the dataset. (PDF) [file pone.0215511.s003.pdf]

Supplementary Material S3

Conditional Inference (CI) classification trees.

Selected conditional inference classification tree predicting the response rates of survey questions using only predictors identified as important during the random forest variable importance evaluation step of the analyses (see Table 1). For each tree, only significant predictors at $p < 0.05$ were retained by the algorithm. Sample size used to build each tree can be calculated by adding sample sizes indicated at each terminal node. The classification accuracy was calculated for each tree using a subset of 20% the dataset.

Indicate your degree of agreement for the following statement:

Climate change impacts are happening slowly enough to let us adapt as it comes

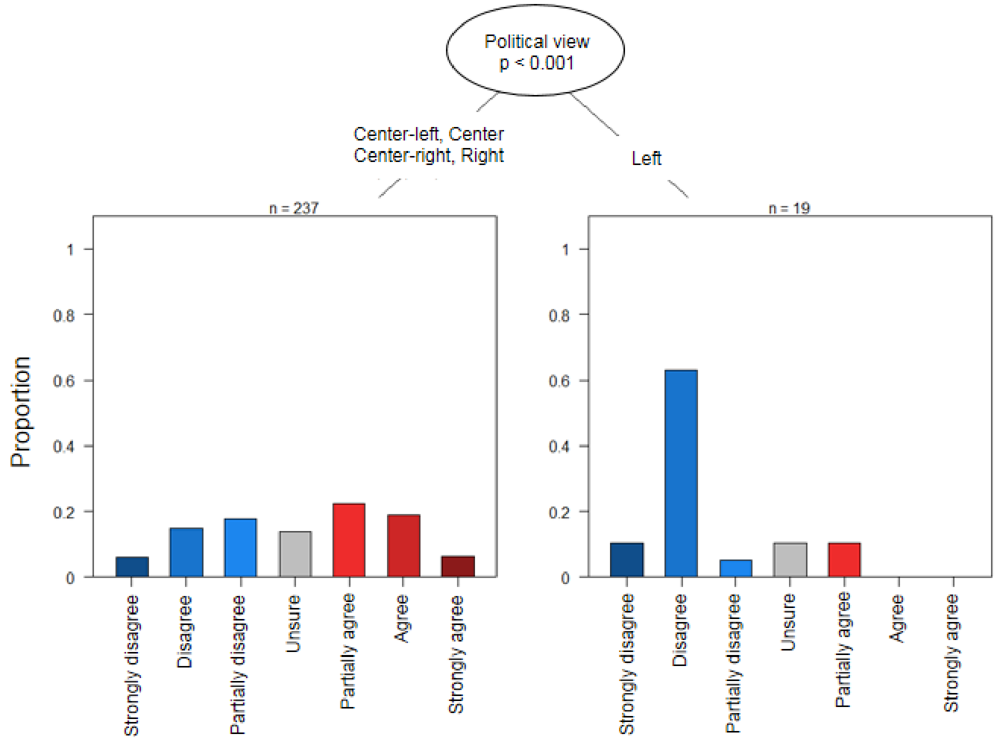

Fig. A. CA = 21.4%.

Indicate your degree of agreement for the following statement:

Climate change is now noticeable in my region

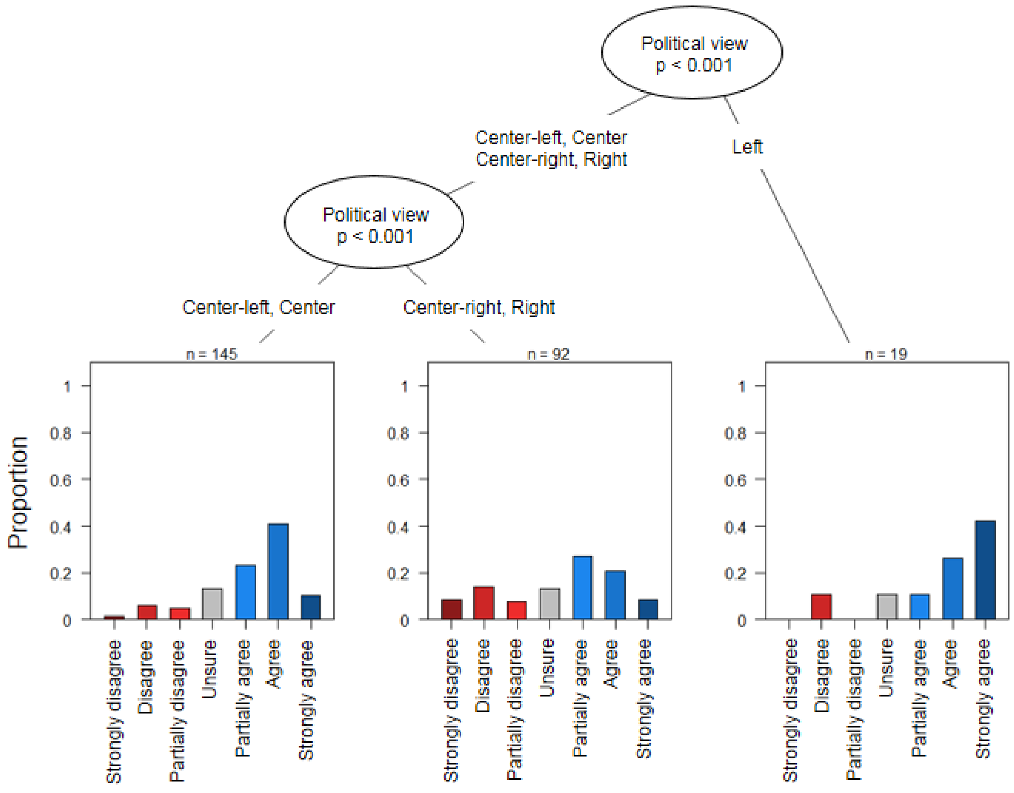

Fig. B. CA = 30.0%.

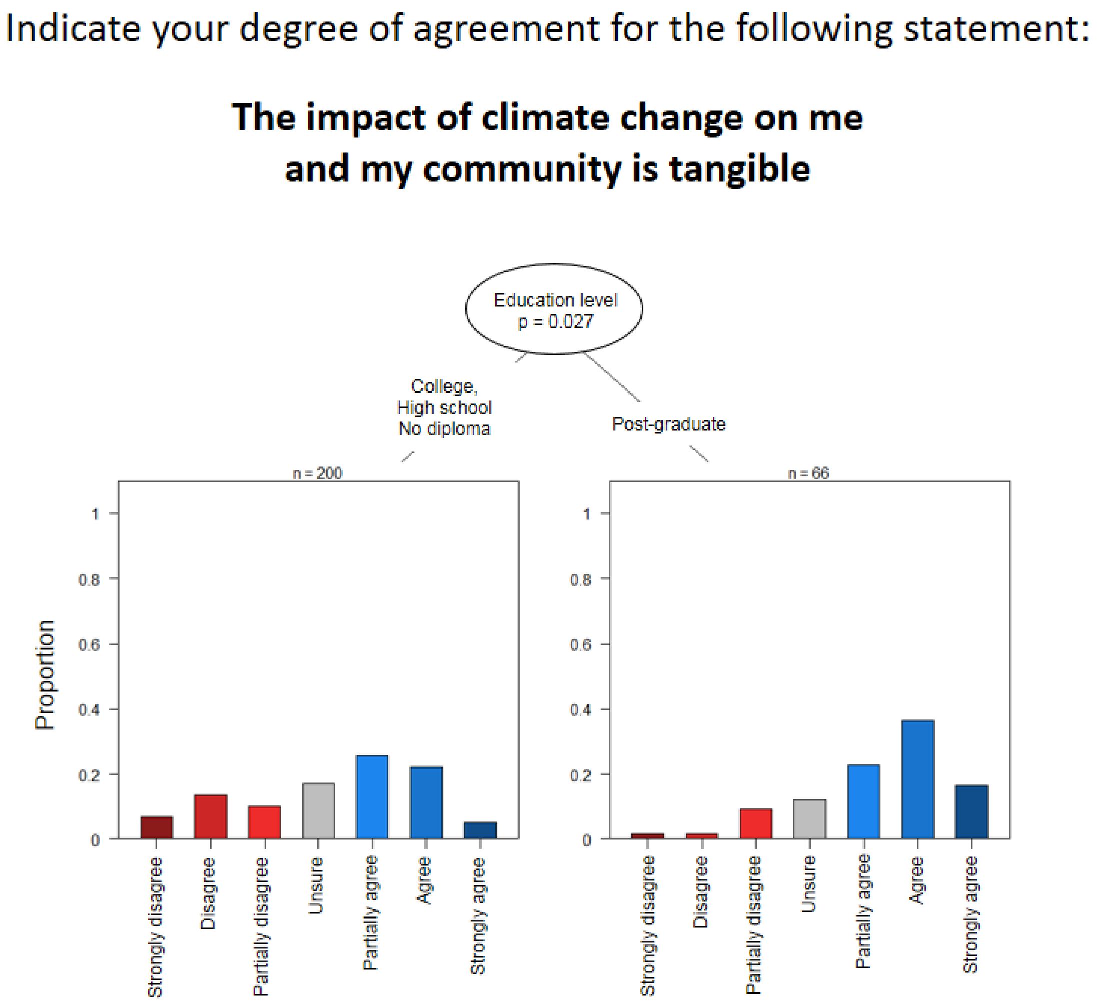
Indicate your degree of agreement for the following statement:
The impact of climate change on me and my community is tangible
Education level
p = 0.027
College, High school No diploma
Post-graduate
n = 200
n = 66
Proportion
1
0.8
0.6
0.4
0.2
0
Strongly disagree
Disagree
Partially disagree
Unsure
Partially agree
Agree
Strongly agree

Fig. C. CA = 25.7%.

Indicate your degree of agreement for the following statement:

The projected impacts of climate change are exaggerated

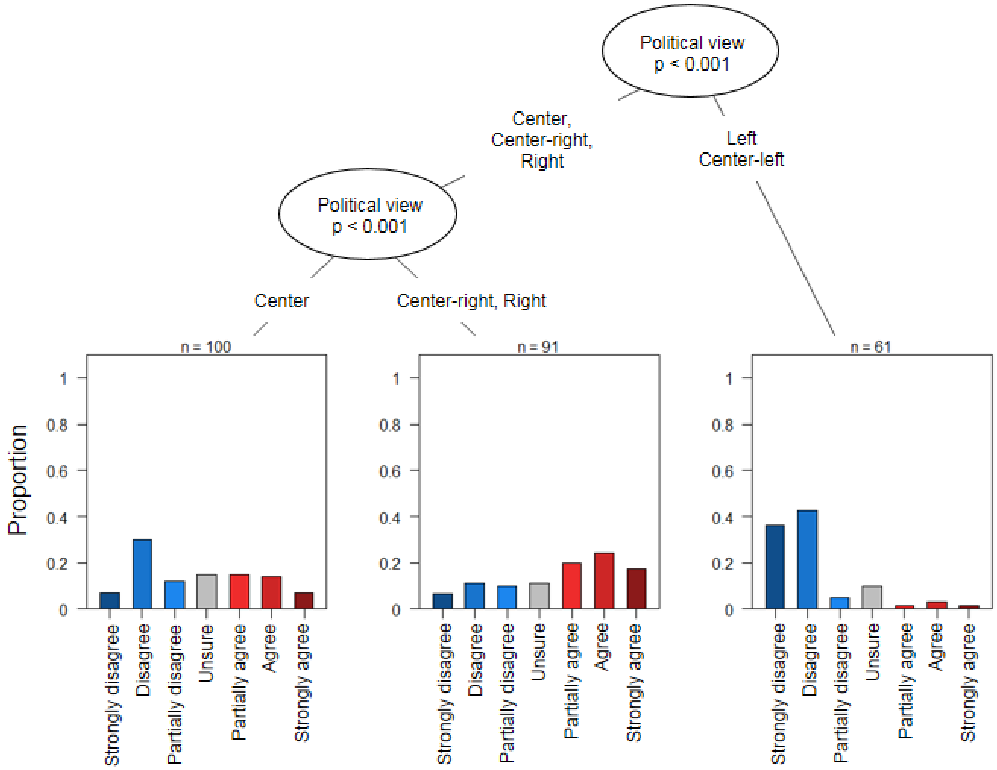

Fig. D. CA = 22.9%.

What do you think is the probability that the following climatic events will happen more frequently in the next 30 years?

Summer heat waves

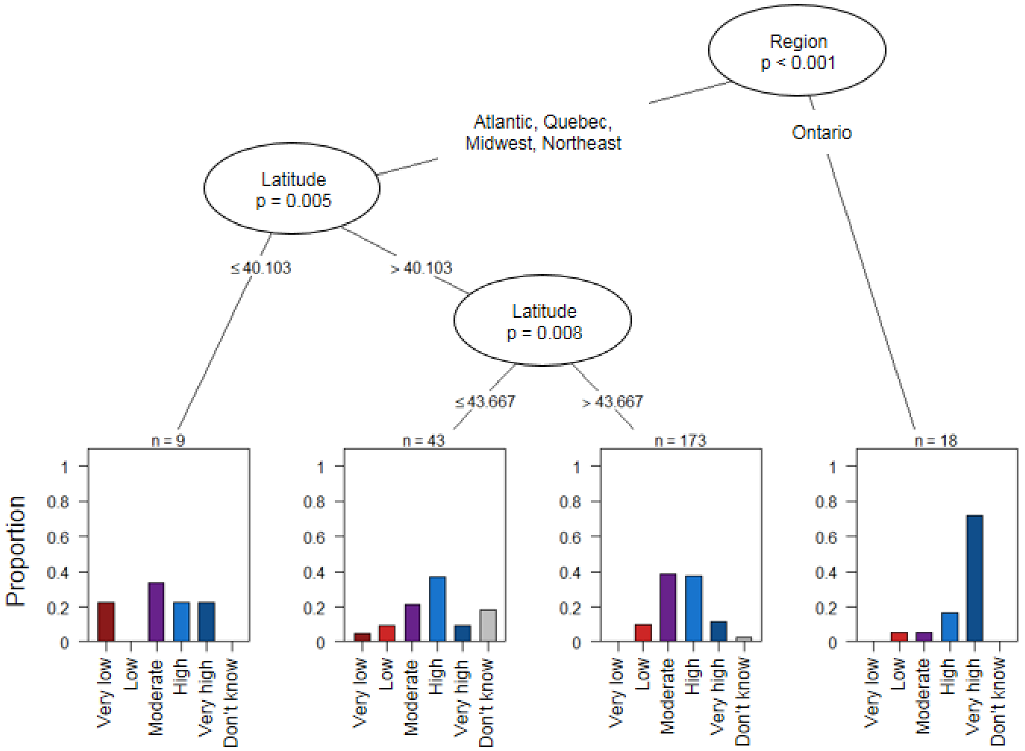

Fig. E. CA = 35.7%.

What do you think is the probability that the following climatic events will happen more frequently in the next 30 years?

High number of winter thaw events

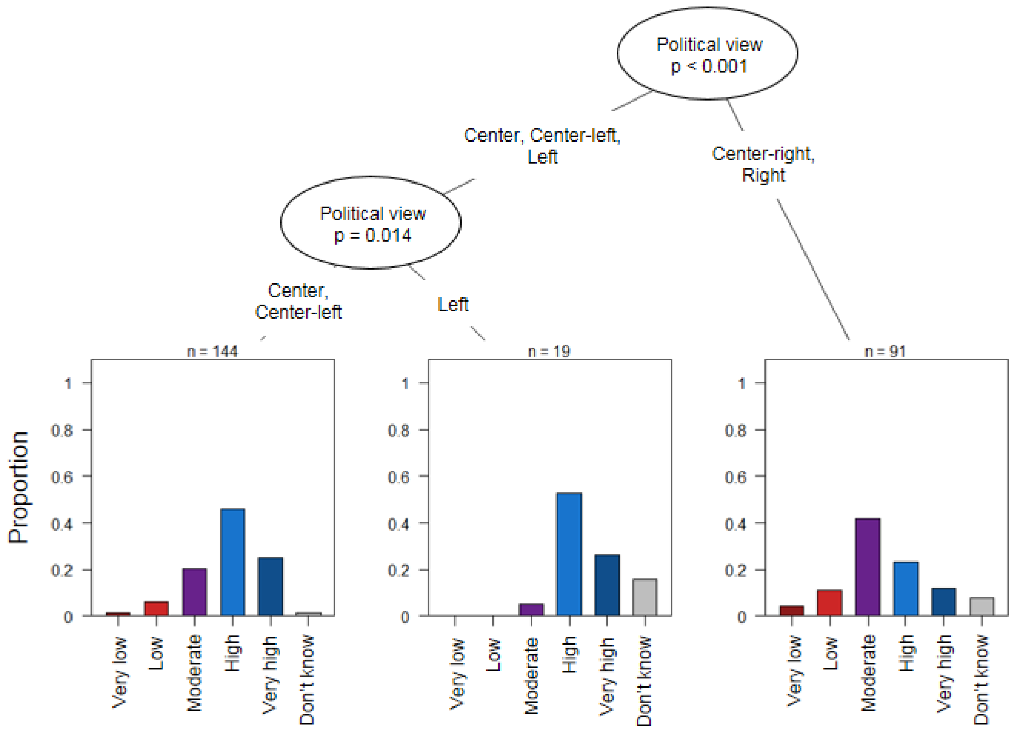

Fig. F. CA = 40.0%.

What do you think is the probability that the following climatic events will happen more frequently in the next 30 years?

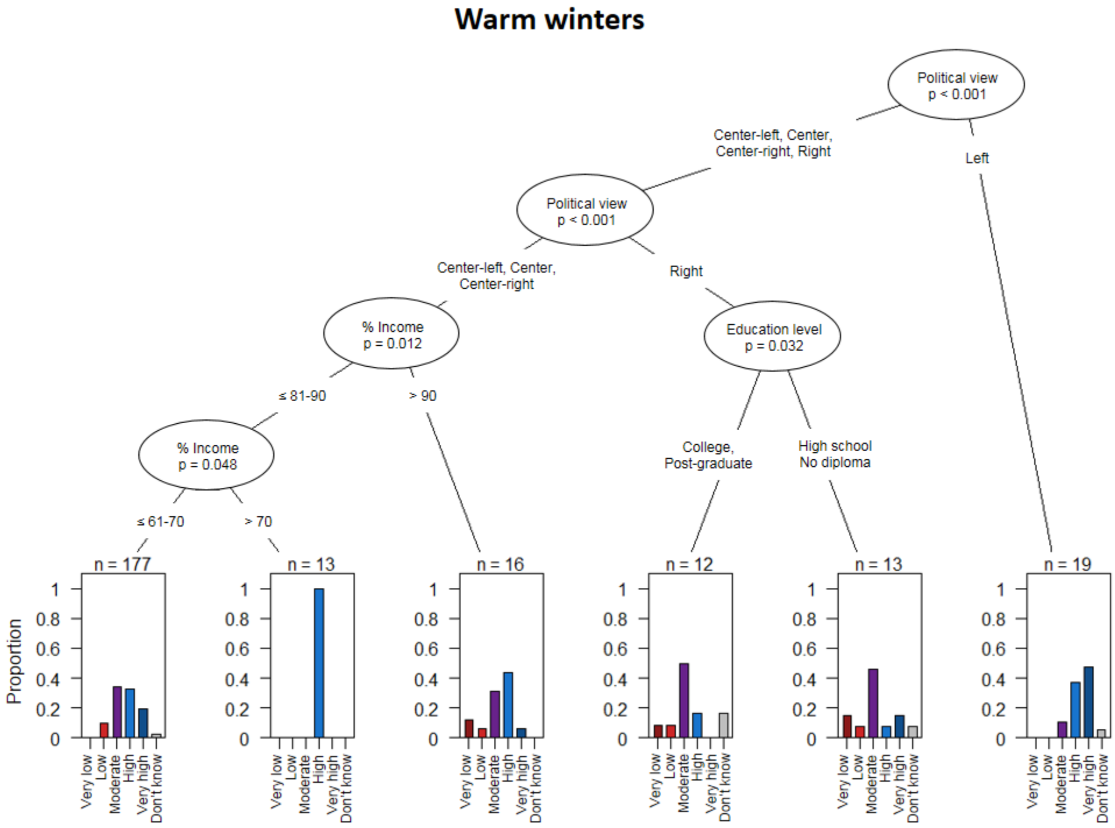

Fig. G. CA = 32.9%.

What do you think is the probability that the following climatic events will happen more frequently in the next 30 years?

Snow and ice storms

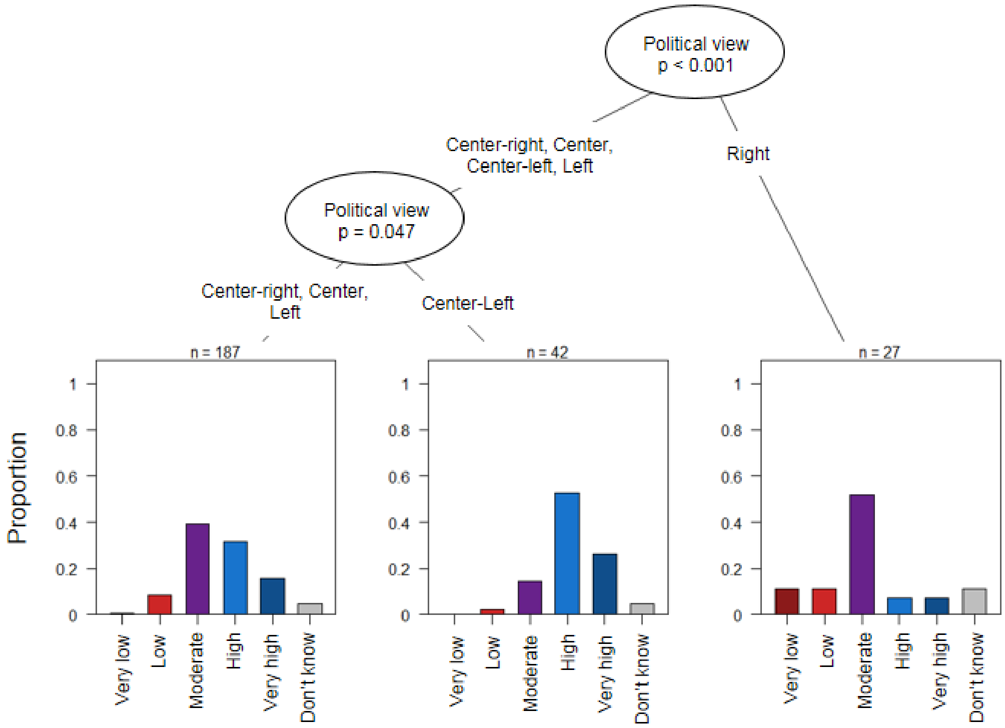

Fig. H. CA = 42.9%.

What do you think is the probability that the following climatic events will happen more frequently in the next 30 years?

Shifts in timing of the spring period in which freeze-thaw events happen

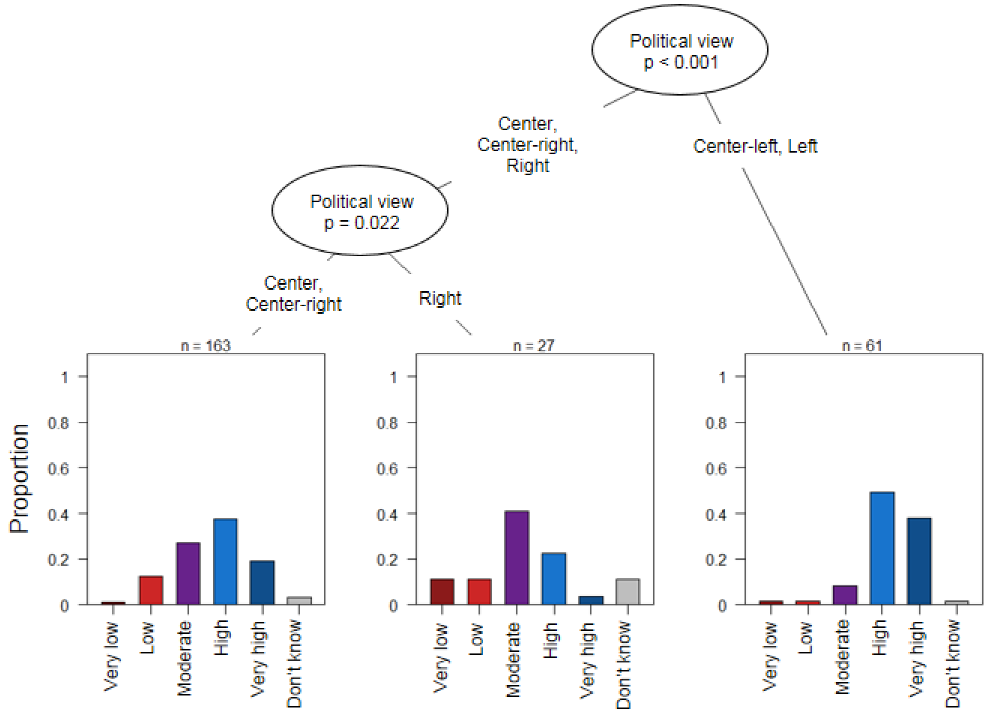

Fig. I. CA = 35.7%.

What do you think is the probability that the following climatic events will happen more frequently in the next 30 years?

Insect outbreaks

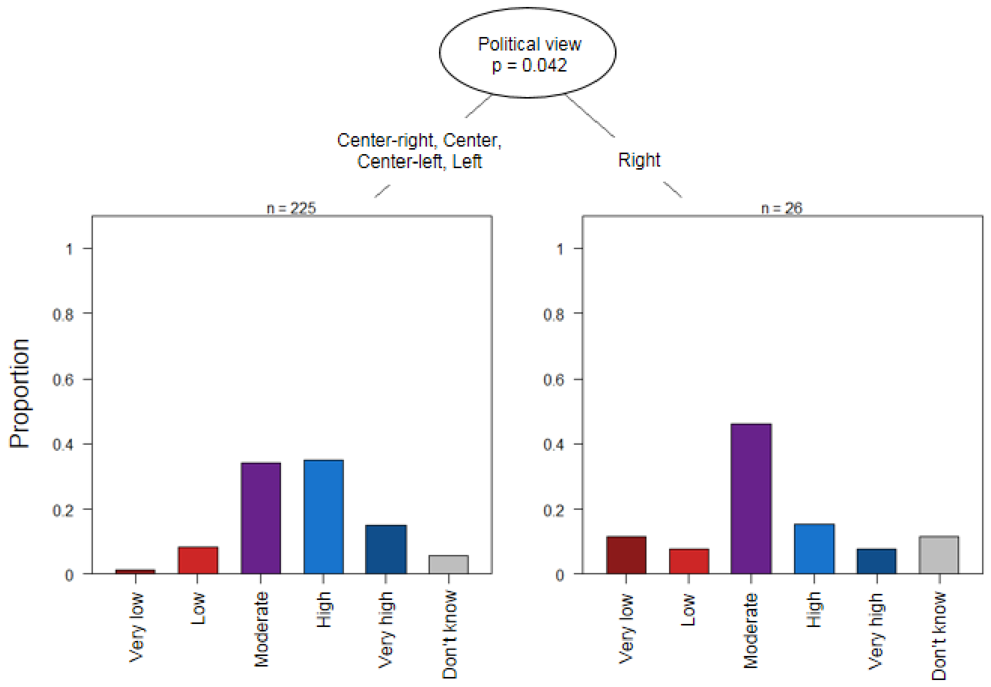

Fig. J. CA = 25.7%.

What do you think is the probability that the following climatic events will happen more frequently in the next 30 years?

High annual mean temperatures

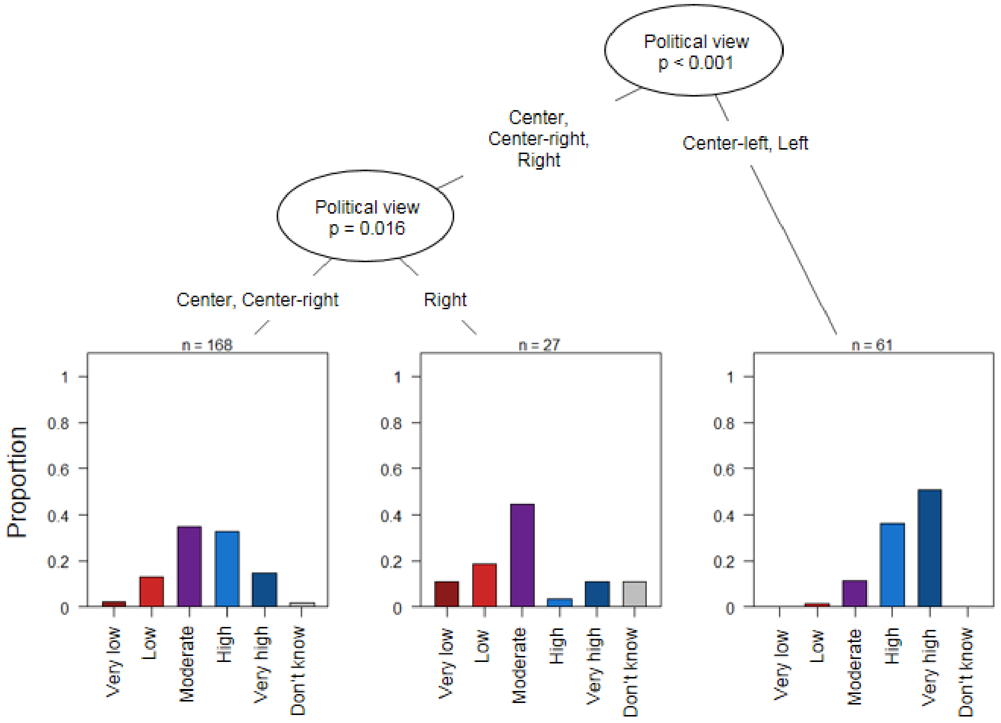

Fig. K. CA = 34.3%.

What do you think is the probability that the following climatic events will happen more frequently in the next 30 years?

Heavy rainfall episodes

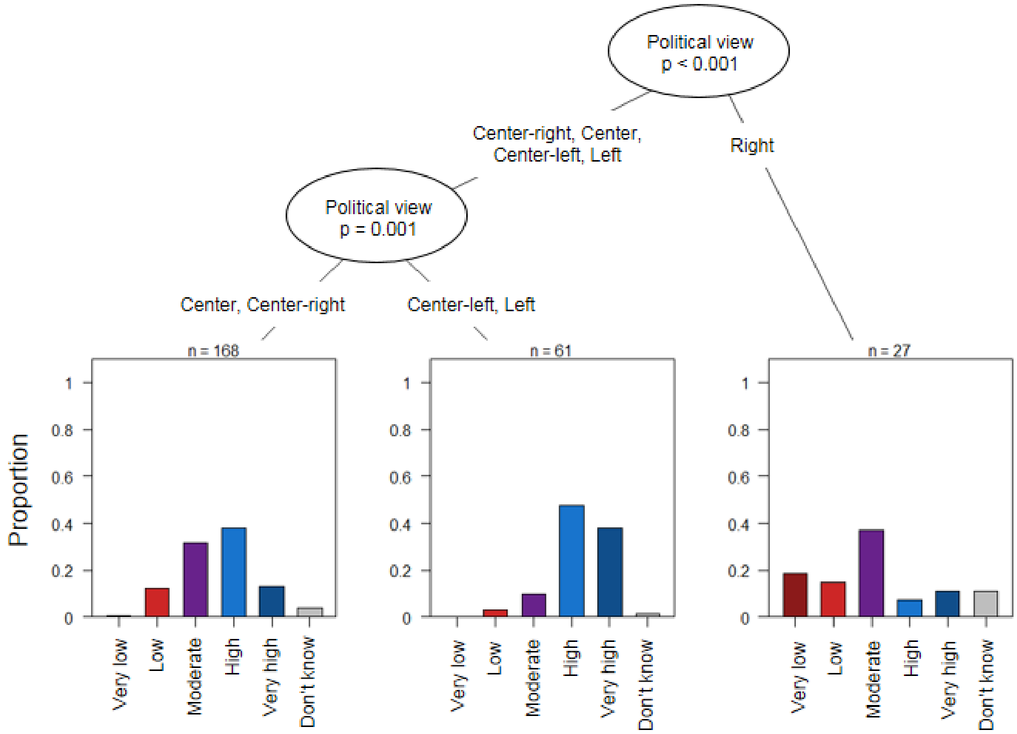

Fig. L. CA = 37.1%.

What do you think is the probability that the following climatic events will happen more frequently in the next 30 years?

Severe windstorms

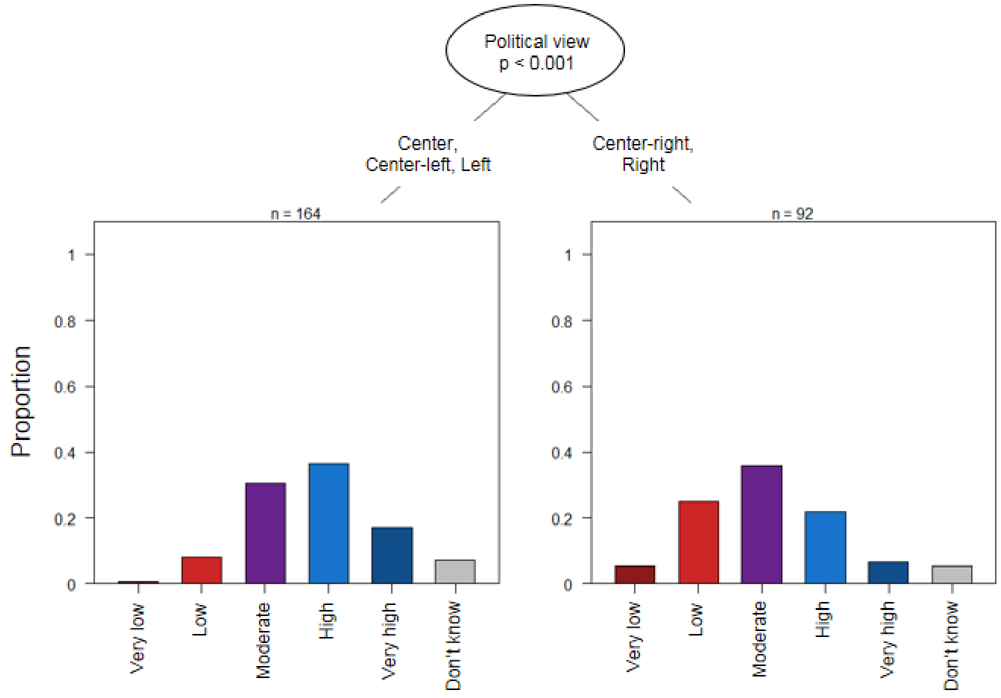

Fig. M. CA = 28.6%.

What do you think is the probability that the following climatic events will happen more frequently in the next 30 years?

Extreme high temperature periods during the spring season that prematurely stop or slow sap flow

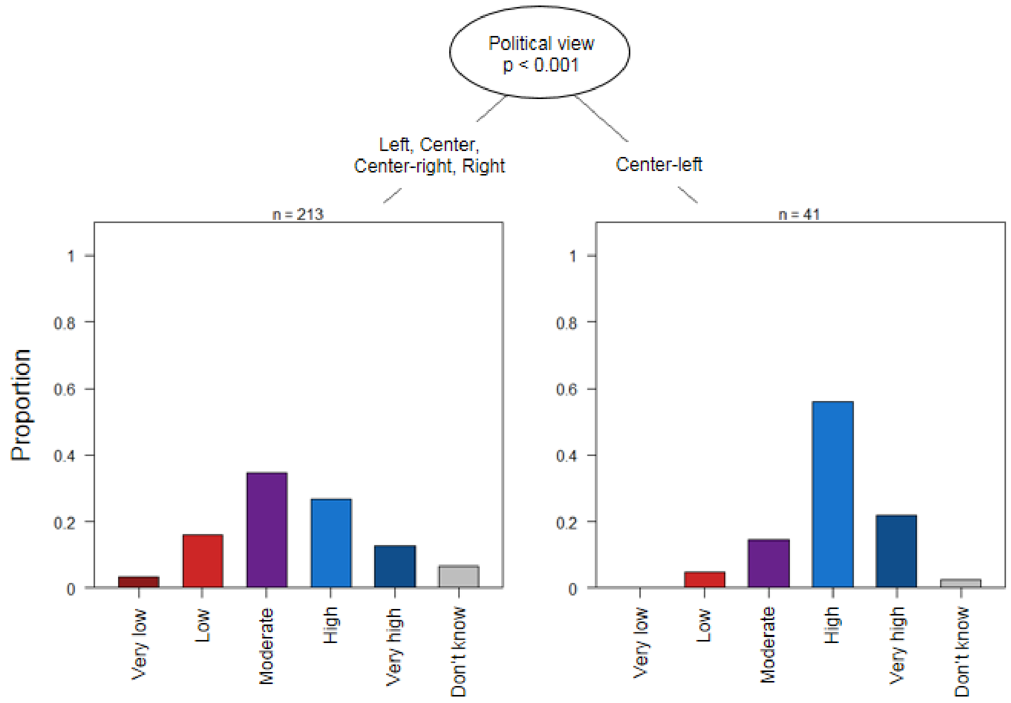

Fig. N. CA = 35.7%.

What do you think is the probability that the following climatic events will happen more frequently in the next 30 years?

Droughts

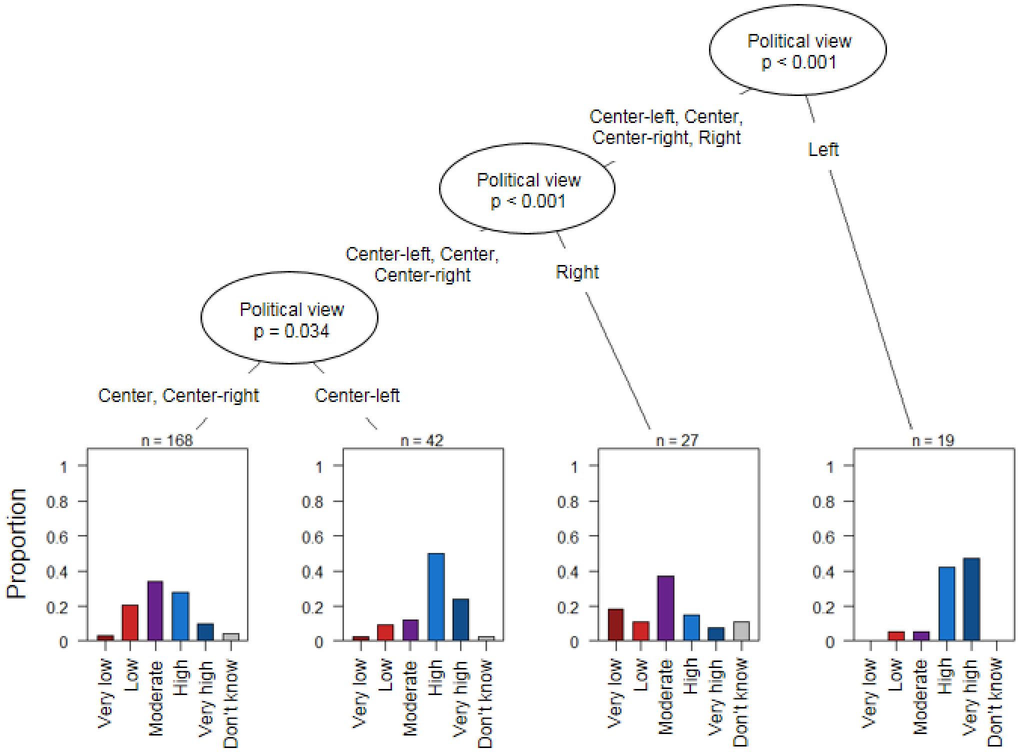

Fig. O. CA = 31.4%.

What do you think is the probability that the following climatic events will happen more frequently in the next 30 years?

Hail storms

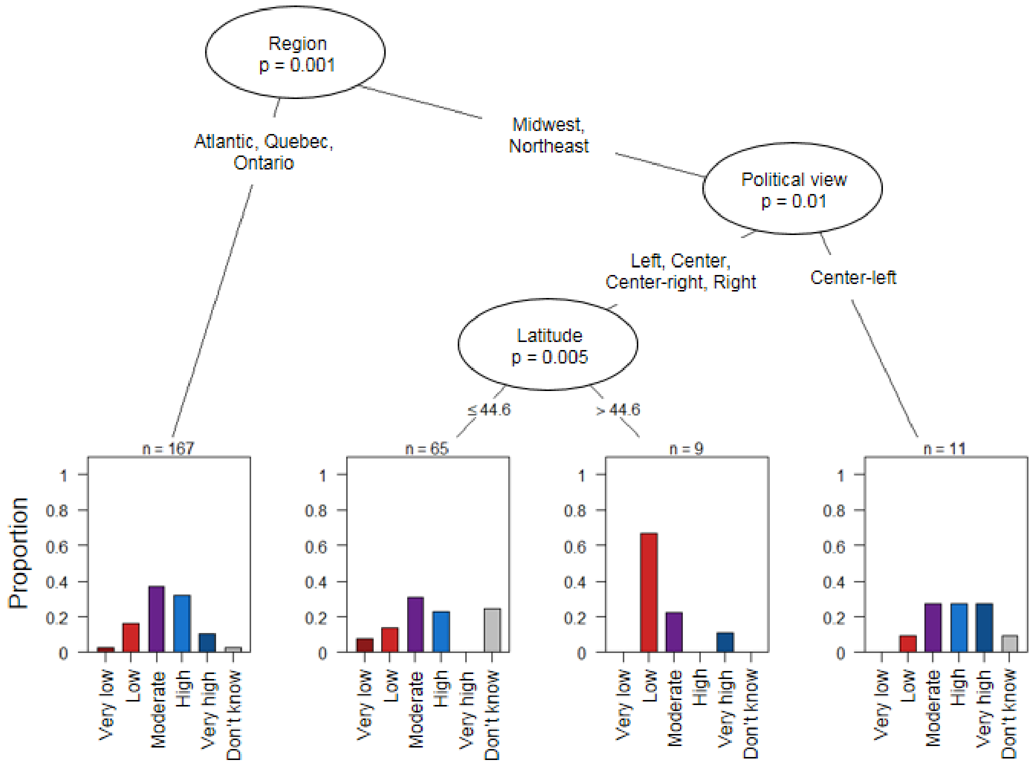

Fig. P. CA = 37.1%.

In the last decades, which of these climate hazards have caused significant damage to your sugar bush?

Ice storm

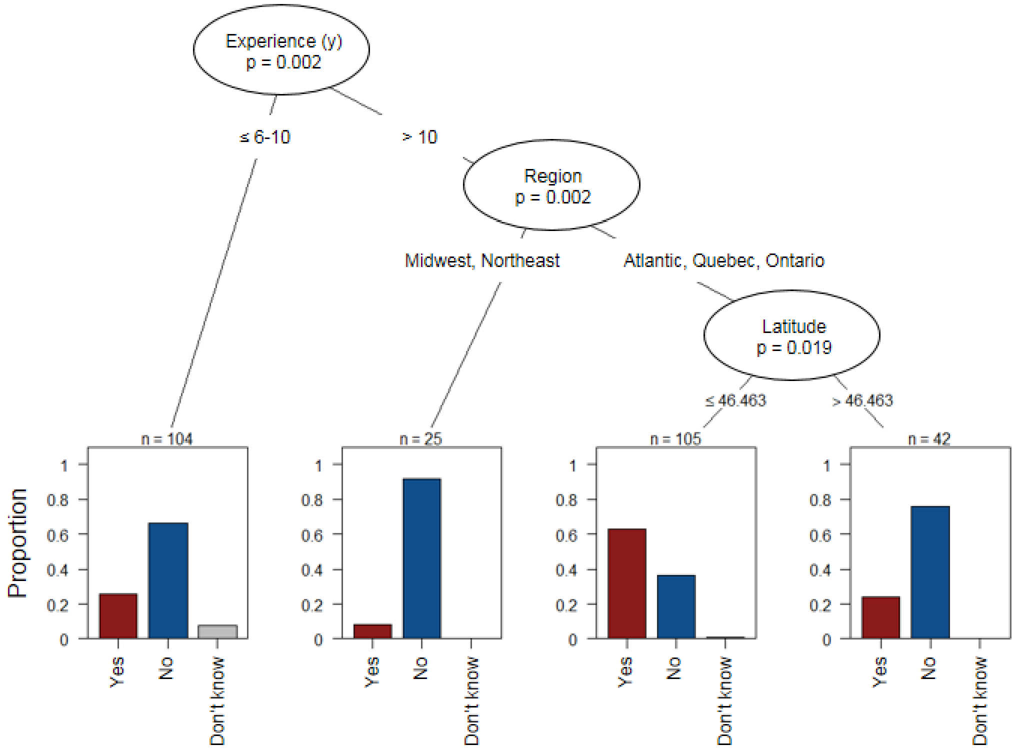

Fig. Q. CA = 51.4%.

In the last decades, which of these climate hazards have caused significant damage to your sugar bush?

Insect outbreak

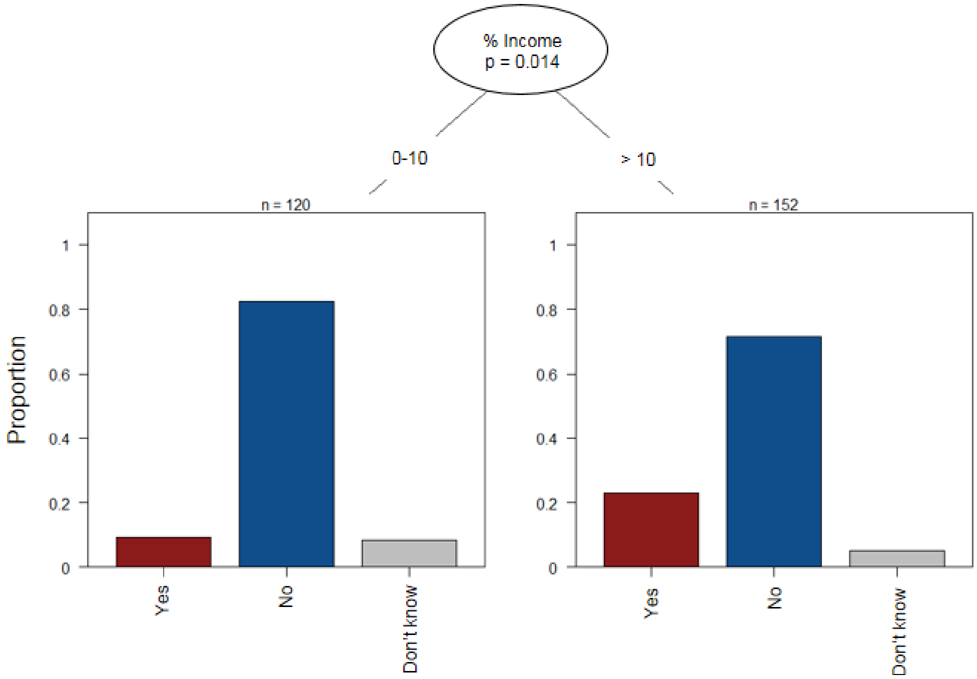

Fig. R. CA = 80.0%.

Indicate your degree of agreement for the following statements:

Maple syrup production is closely linked to climate change

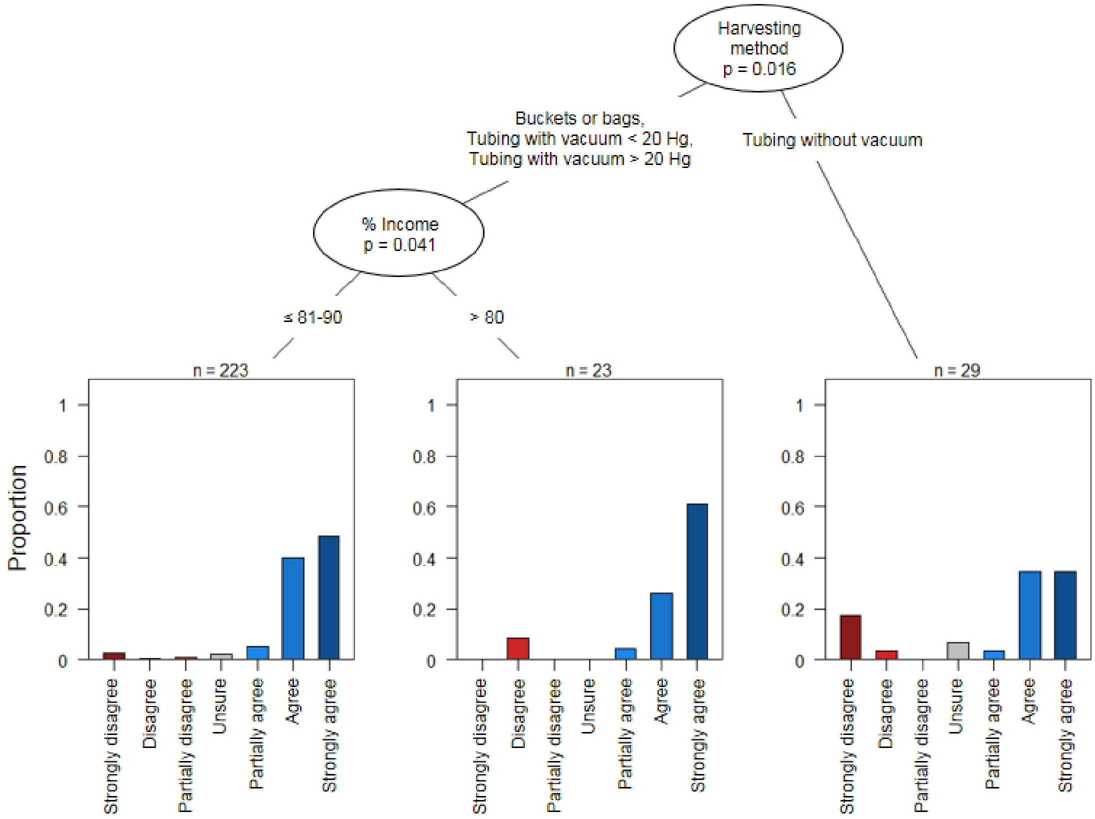

Fig. S. CA = 42.9%.

Indicate your degree of agreement for the following statements:

Climate change has led to variability in the beginning of the tap season between years

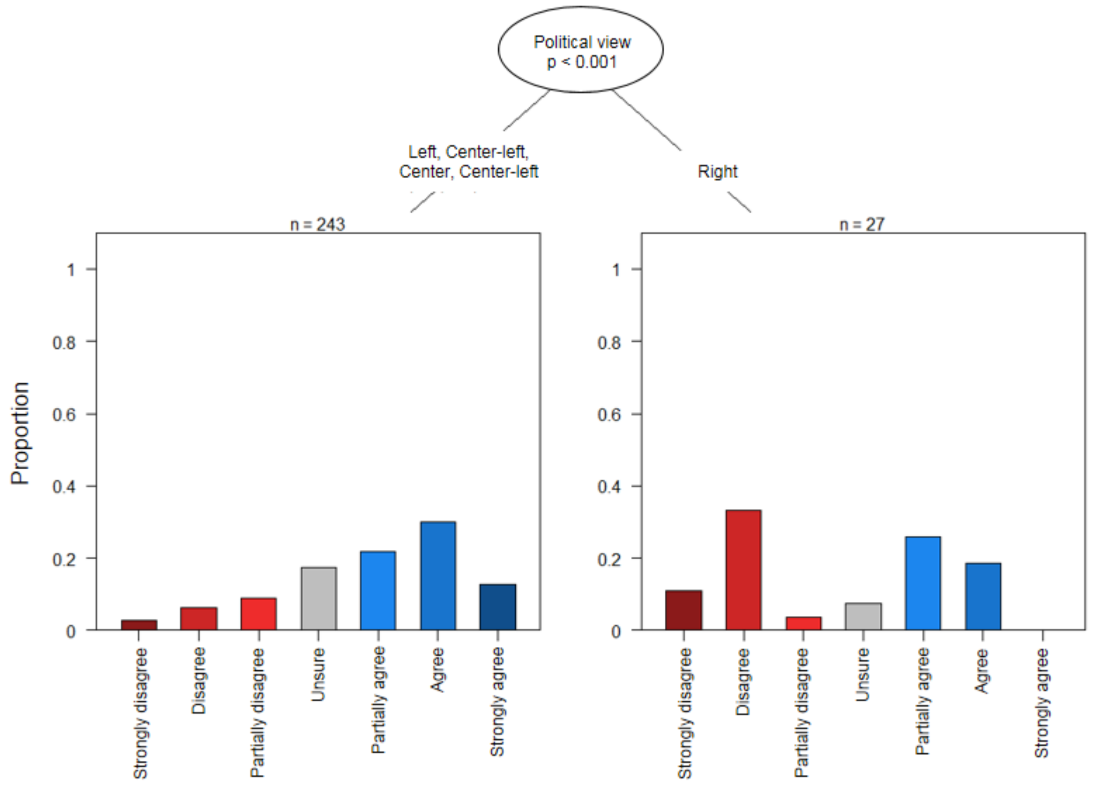

Fig. T. CA = 31.4%.

Indicate your degree of agreement for the following statements:

It's now easy to determine the best moment to tap maples

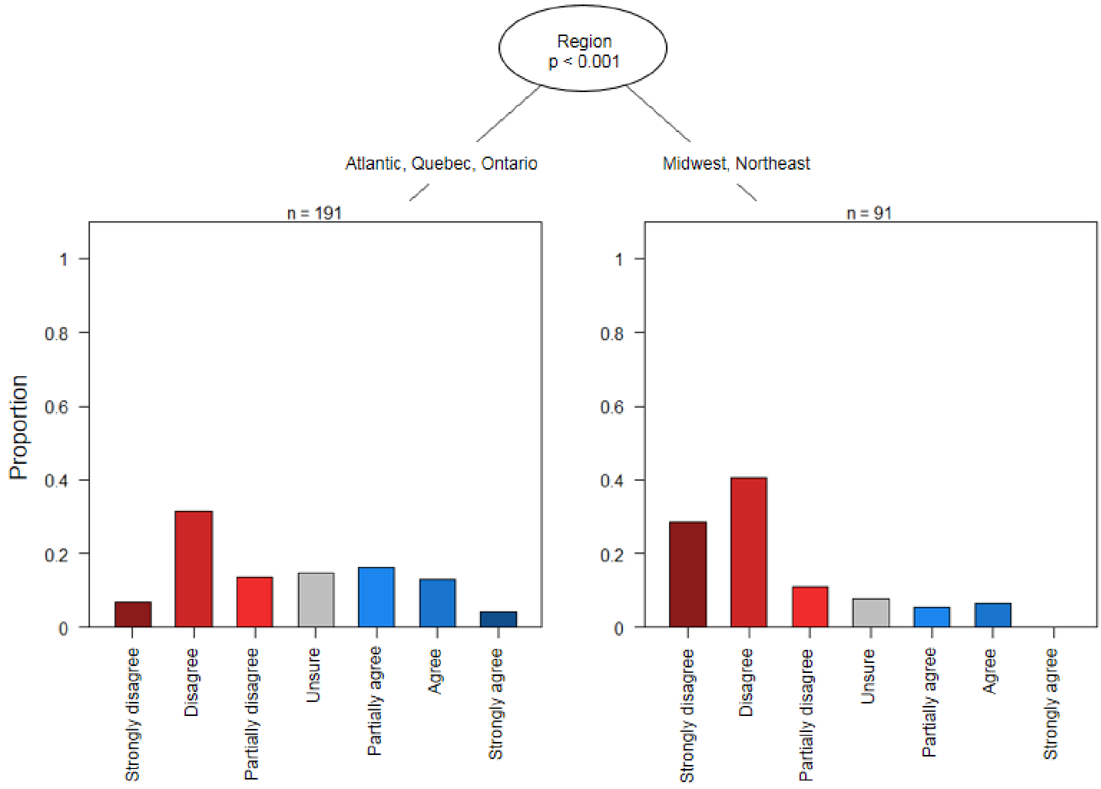

Fig. U. CA = 24.3%.

Indicate your degree of agreement for the following statements:

In my sugar bush, I have observed an increase in maple dieback because of climate change

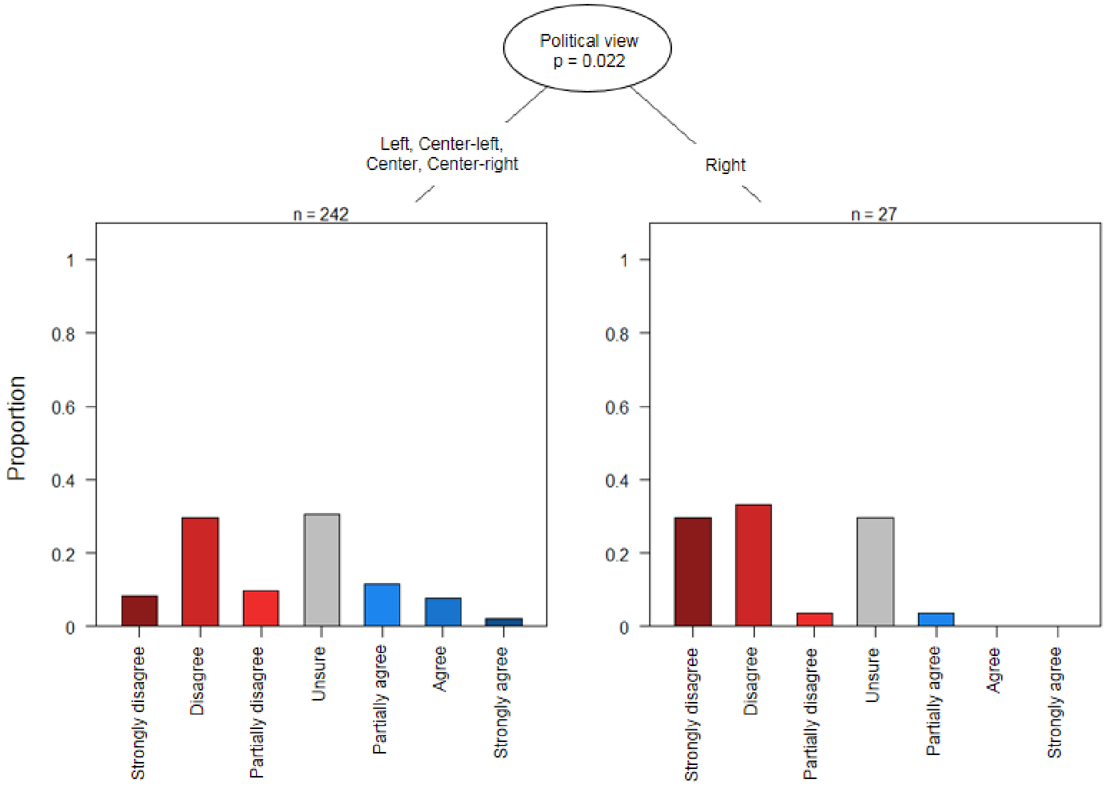

Fig. V. CA = 27.1%.

Indicate your degree of agreement for the following statements:

In the next 30 years, the beginning of the sap collection season is going to happen earlier because of climate change

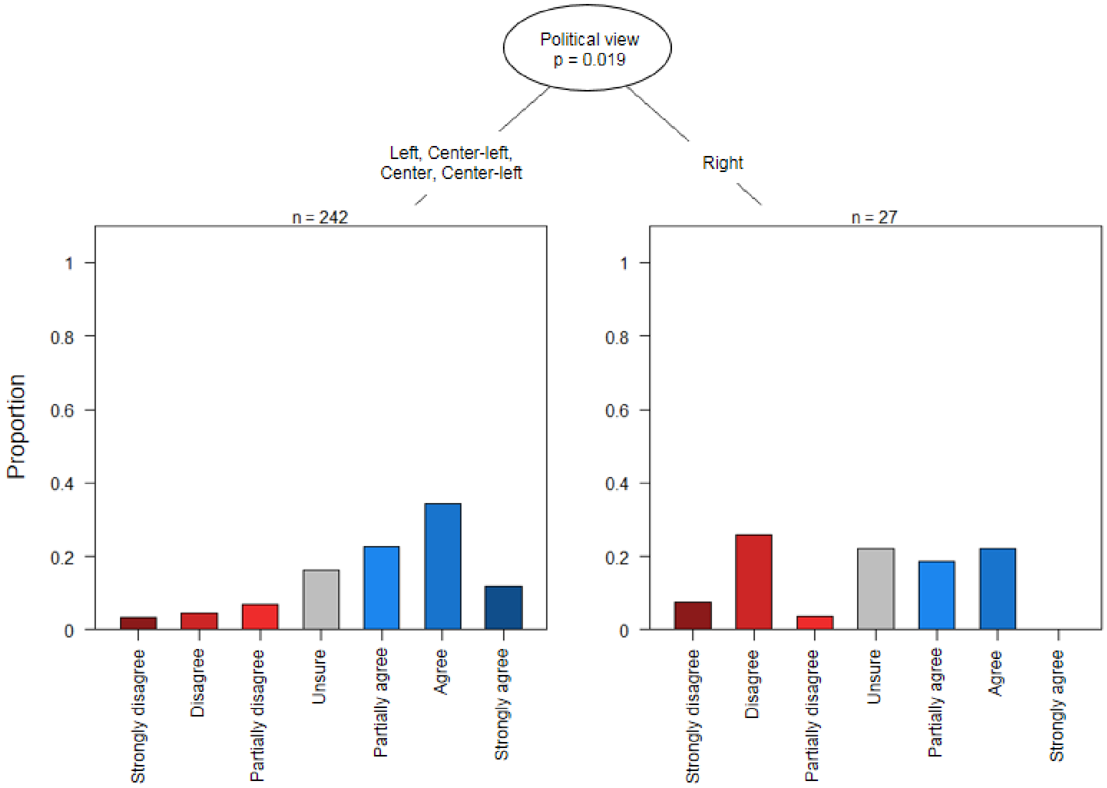

Fig. W. CA = 28.6%.

Indicate your degree of agreement for the following statements:

In the next 30 years, climate change will lead to variability in the beginning of the sap collection season between years

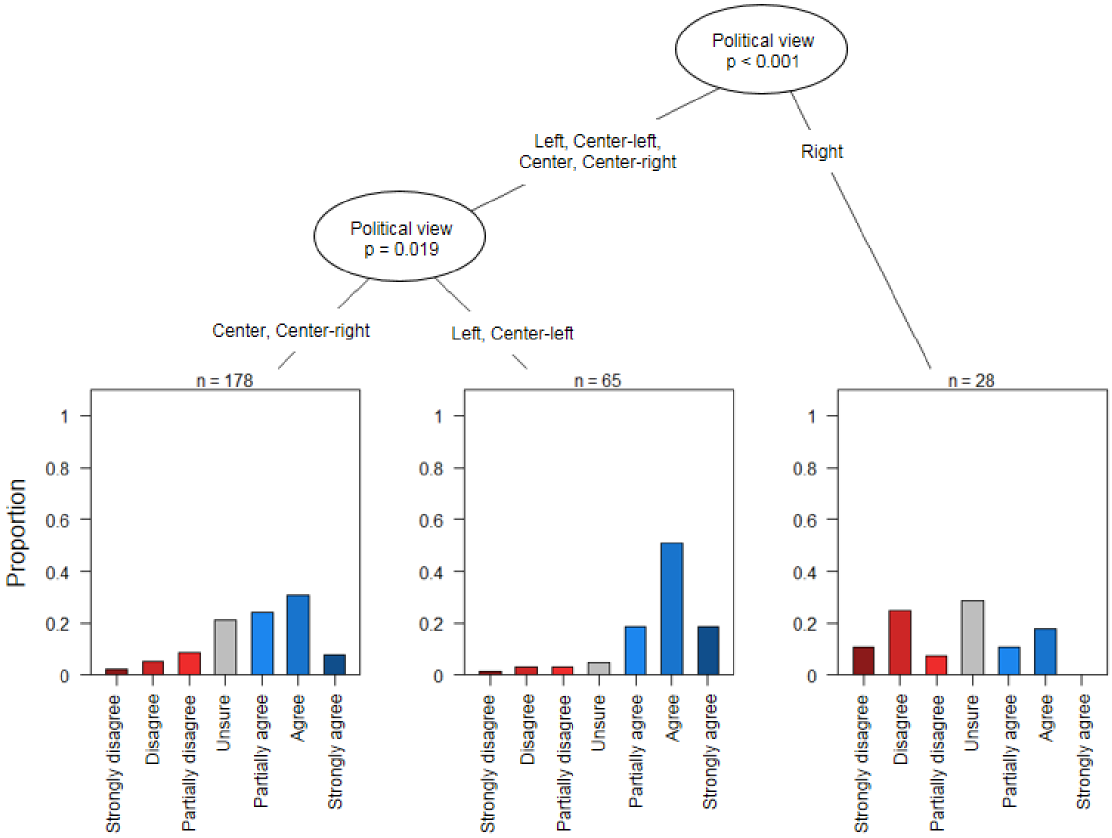

Fig. X. CA = 30.0%.

Indicate your degree of agreement for the following statements:

Possible adaptations to climate change are numerous for the maple syrup industry

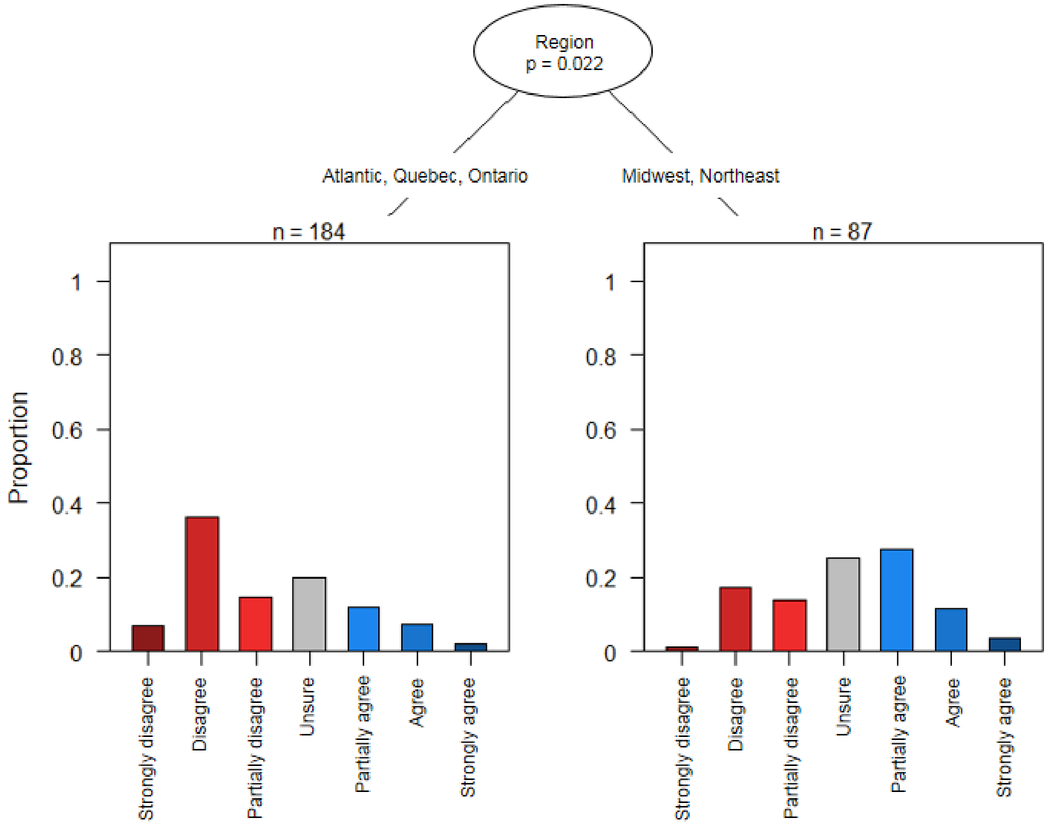

Fig. Y. CA = 30.0%.

Indicate your degree of agreement for the following statements:

have a wide knowledge of the newest tapping technologies (e.g., high-vacuum tubing, new spouts, liming and fertilizing, reverse osmosis, sylvicultural management, tube cleaning/spout replacement)

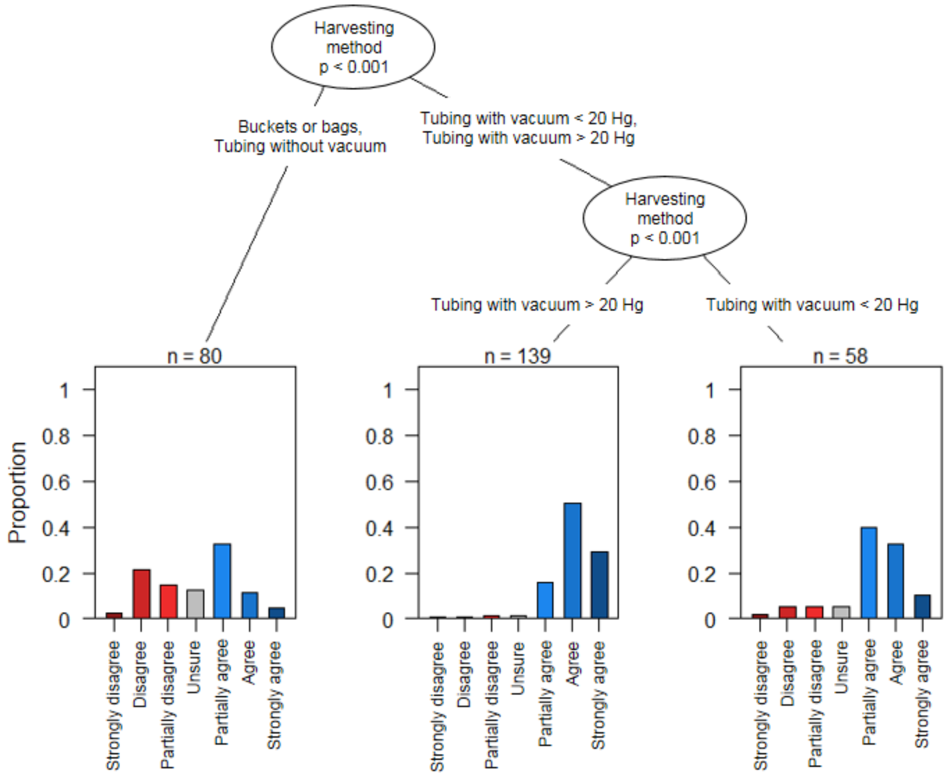

Fig. Z. CA = 37.1%.

Indicate your degree of agreement for the following statements:

New maple syrup technologies will help me face the new challenges coming from climate change

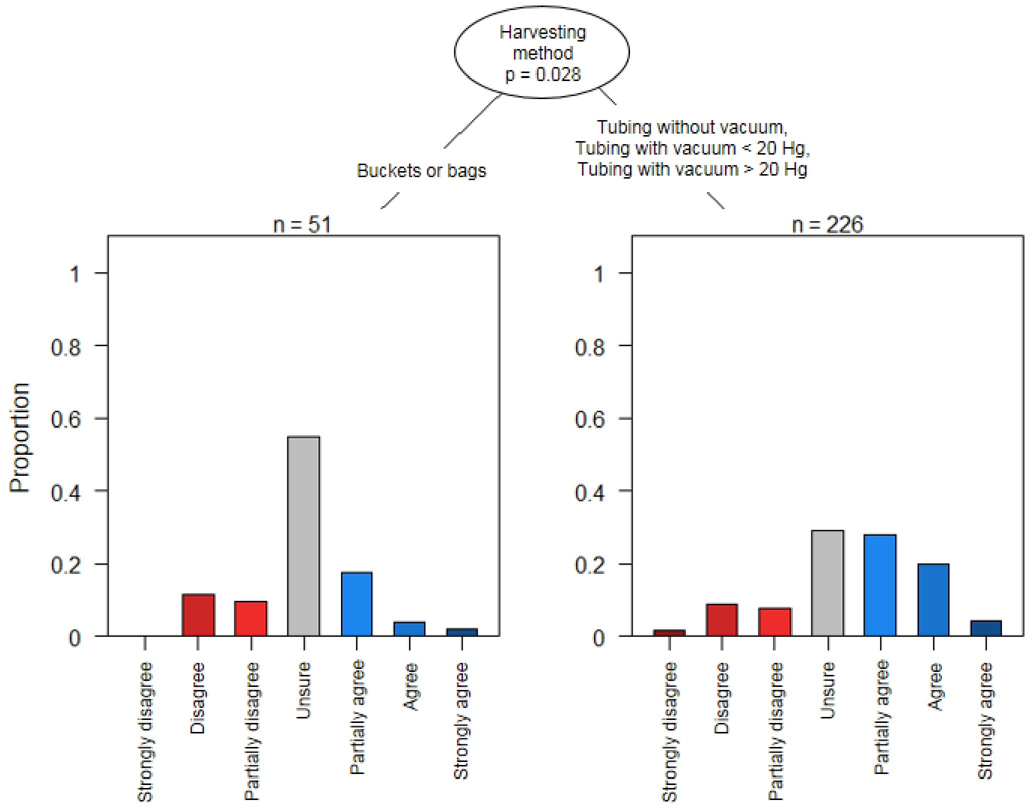

Fig. AA. CA = 21.4%.

Indicate your degree of agreement for the following statements:

It is highly probable that I will adopt climate adaptation strategies if I think it could increase my maple syrup production

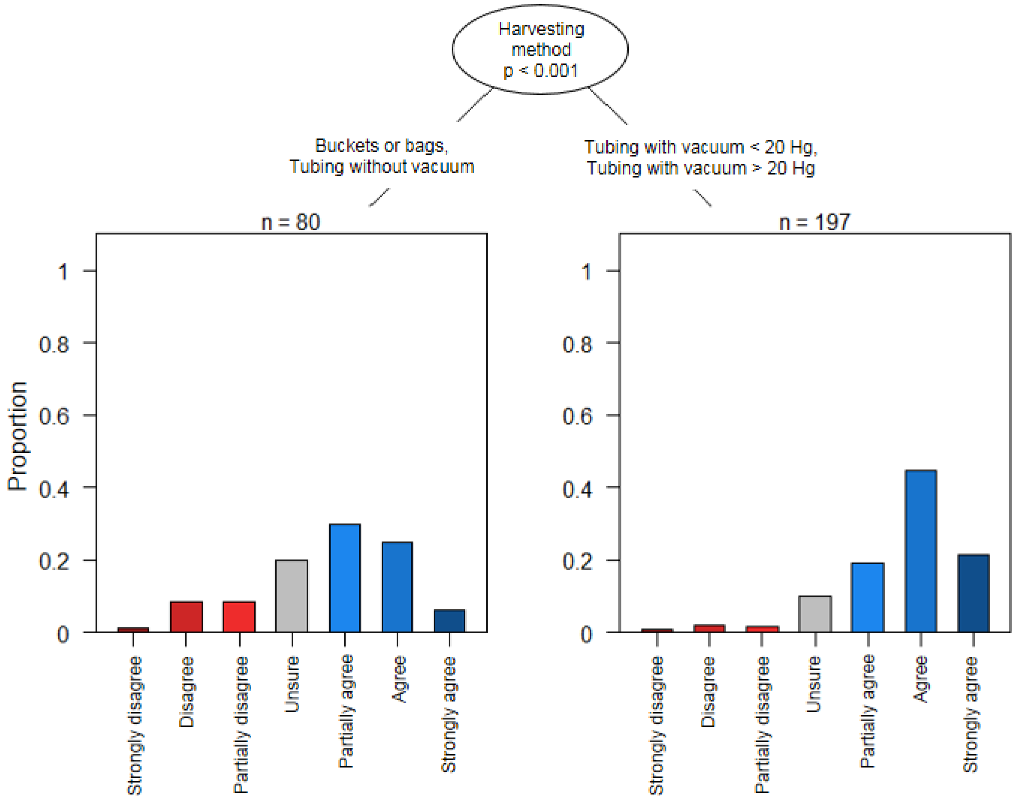

Fig. AB. CA = 34.3%.

Indicate your degree of agreement for the following statements:

Before making any changes to adapt to climate change, I will wait to see what effects it has on my maple syrup production

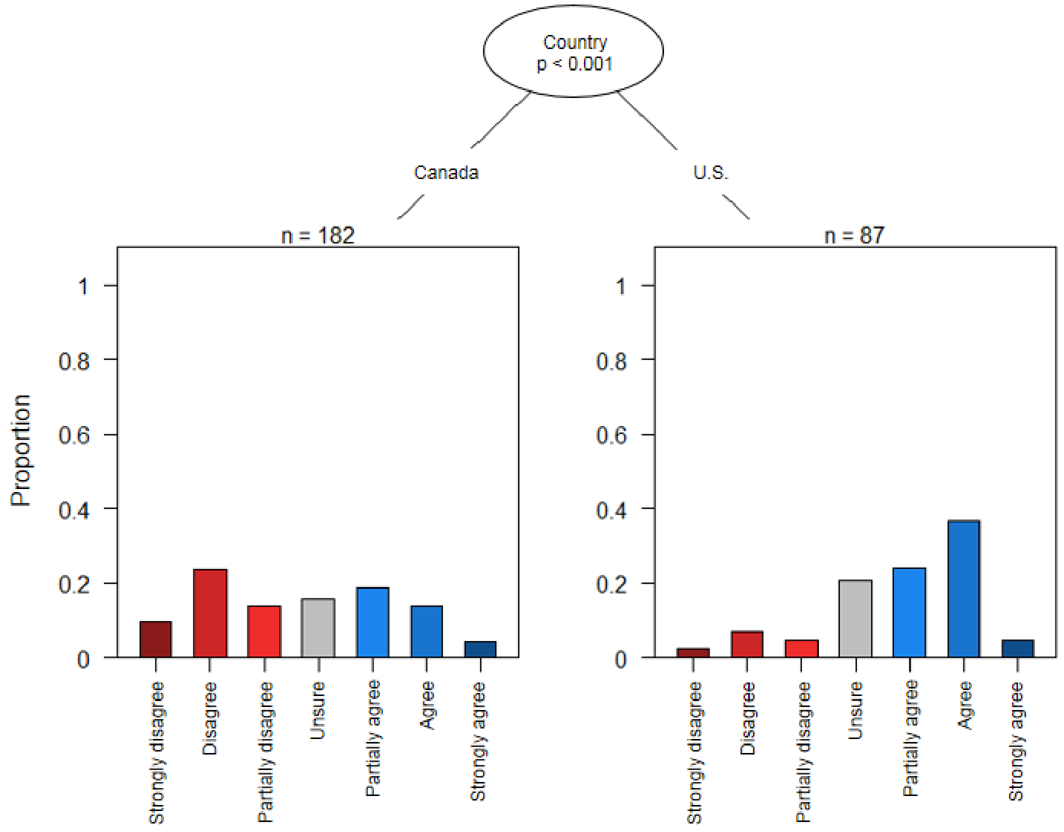

Fig. AC. CA = 21.4%.

Which of these adaptation measures would allow producers in general to effectively adapt to climate change?

Doing sylvicultural management in your sugar bush, for example, maintain the density of trees at a good level or to favor biodiversity

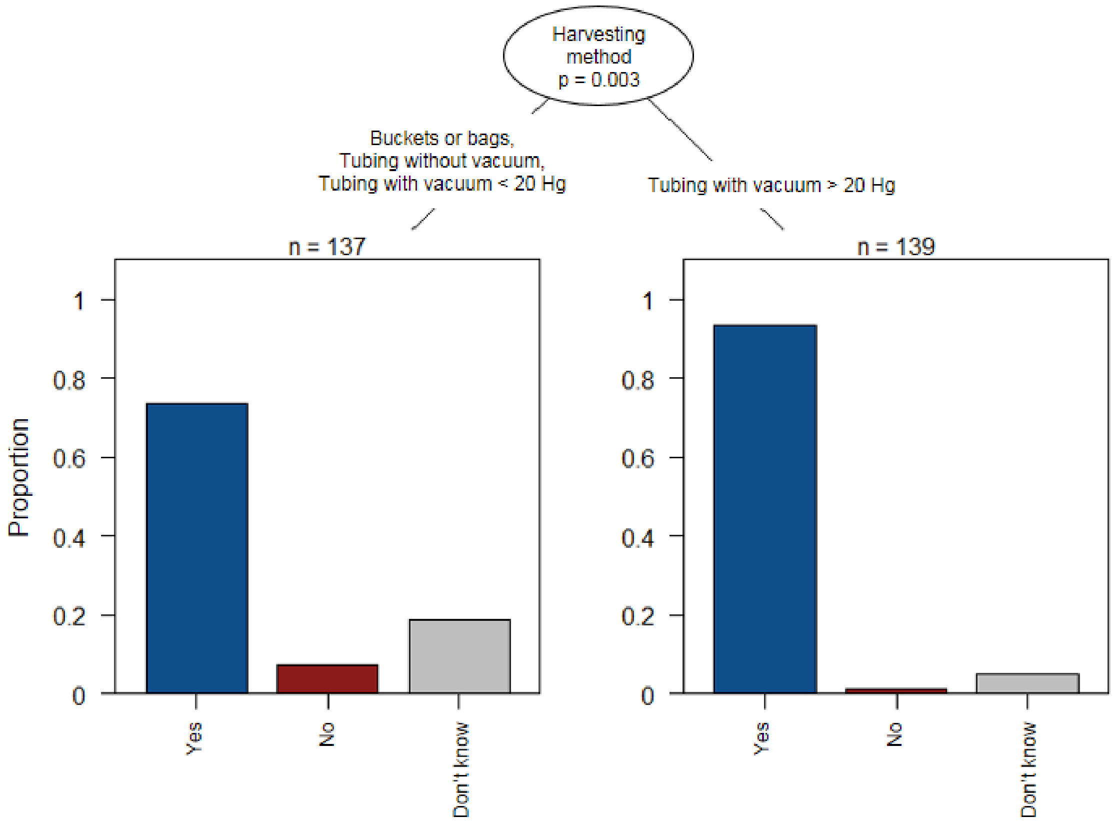

Fig. AD. CA = 85.7%.

Which of these adaptation measures would allow producers in general to effectively adapt to climate change?

Adopting strong sanitation practices (tubing and spout cleaning and/or annual spout replacement)

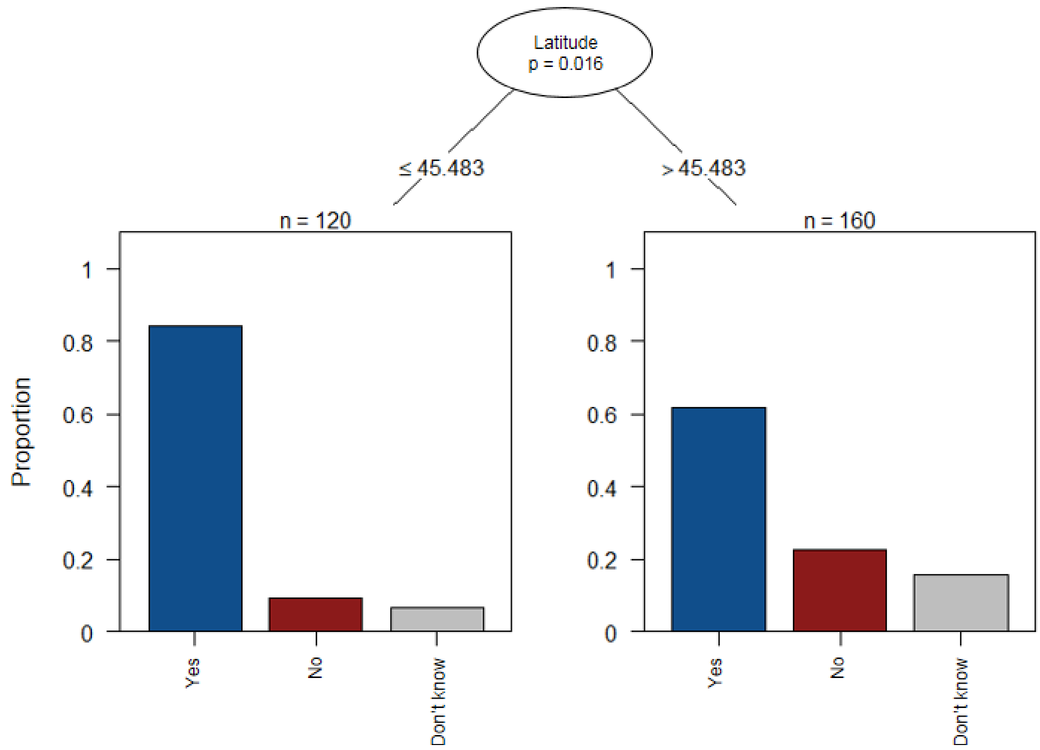

Fig. AE. CA = 62.9%.

Which of these adaptation measures would allow producers in general to effectively adapt to climate change?

Tapping earlier in the year

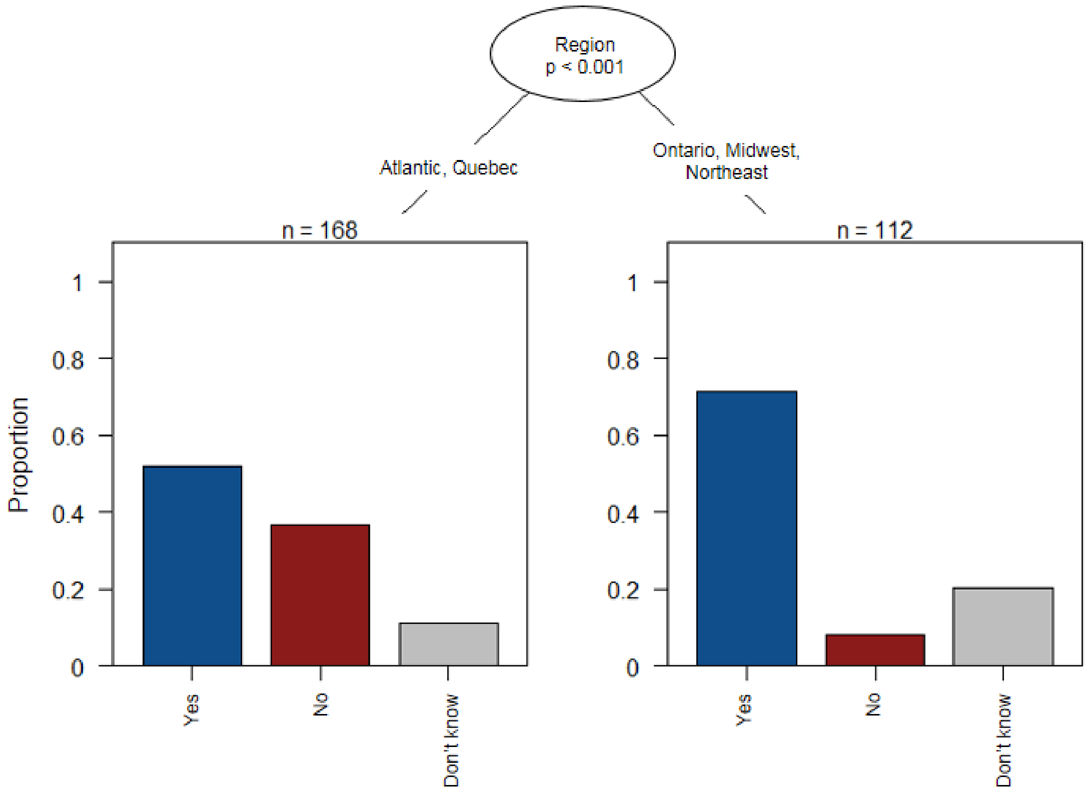

Fig. AF. CA = 57.1%.

Which of these adaptation measures would allow producers in general to effectively adapt to climate change?

Installing a high-vacuum tubing system for sap collection

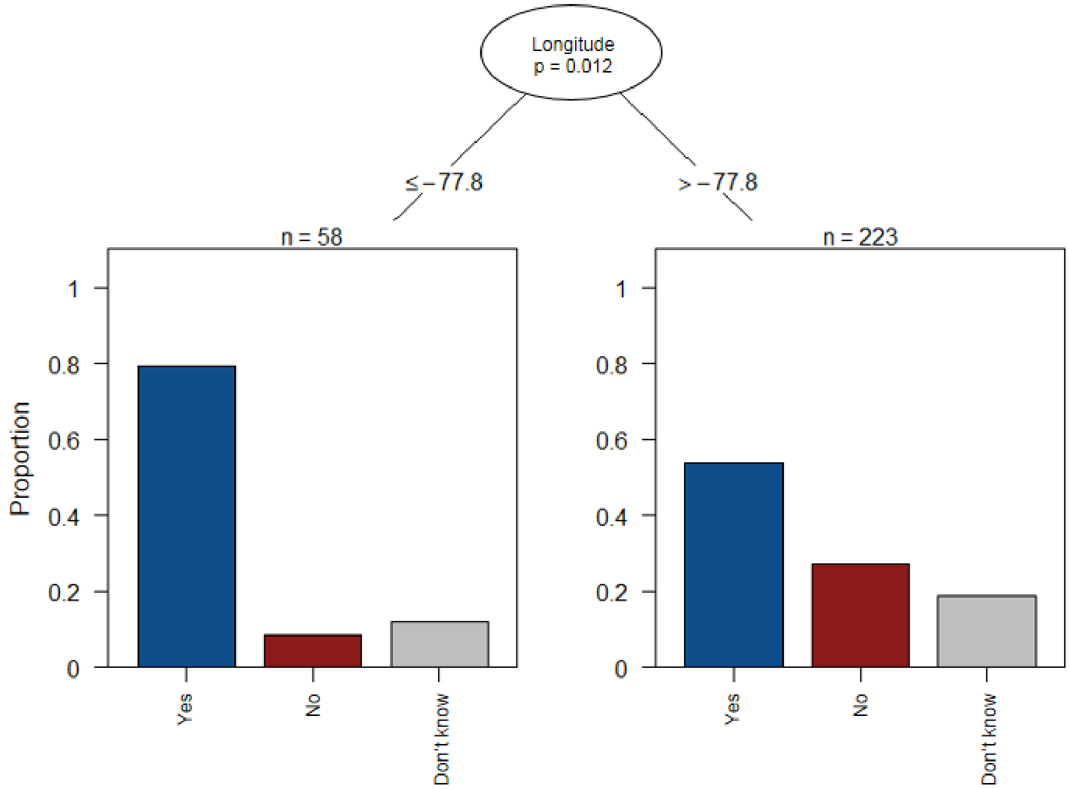

Fig. AG. CA = 58.6%.

Which of these adaptation measures would allow producers in general to effectively adapt to climate change?

Liming and fertilizing to limit maple dieback

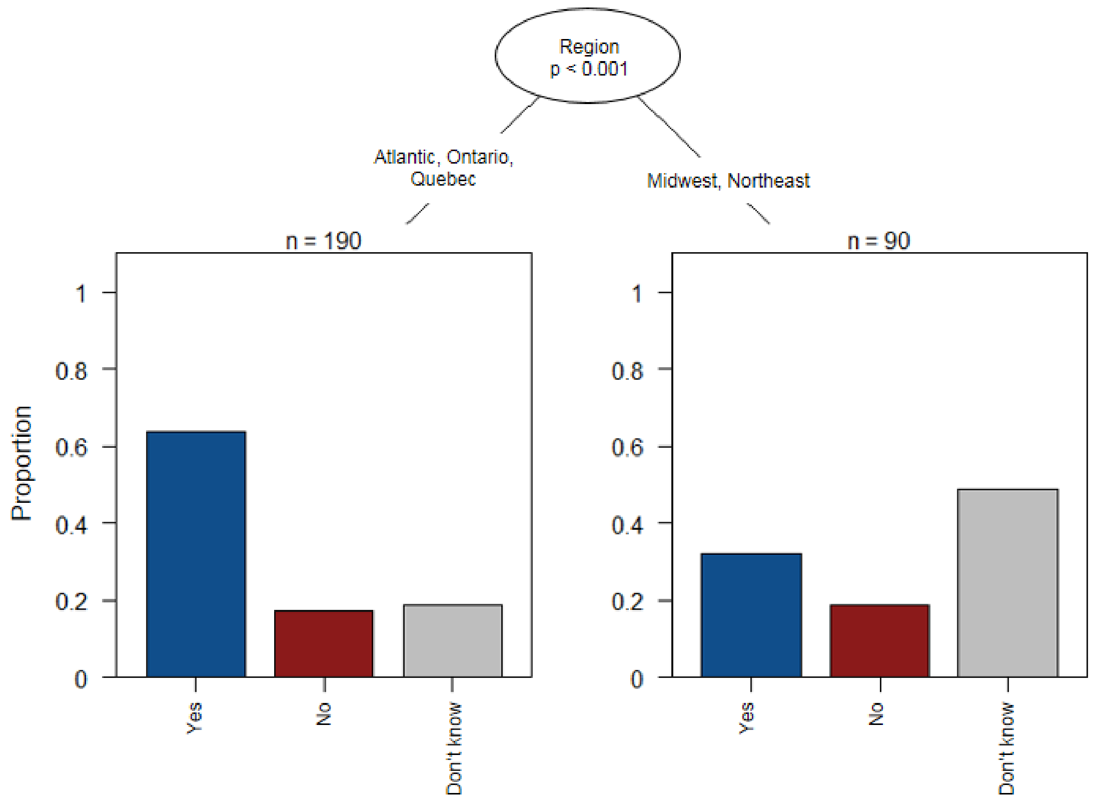

Fig. AH. CA = 57.1%.

Which of these adaptation measures would allow producers in general to effectively adapt to climate change?

Tapping red maples

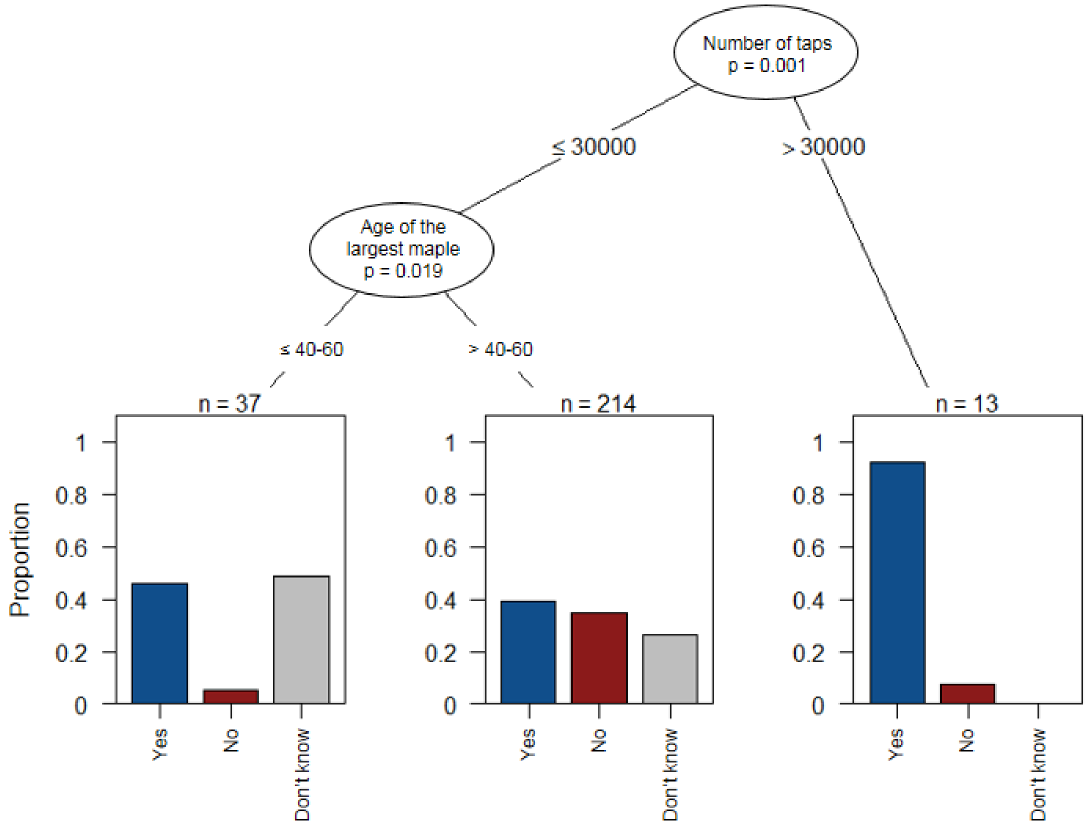

Fig. AI. CA = 38.6%.

Which of these adaptation measures would you like to use yourself?

Keeping track of new research about maple syrup production

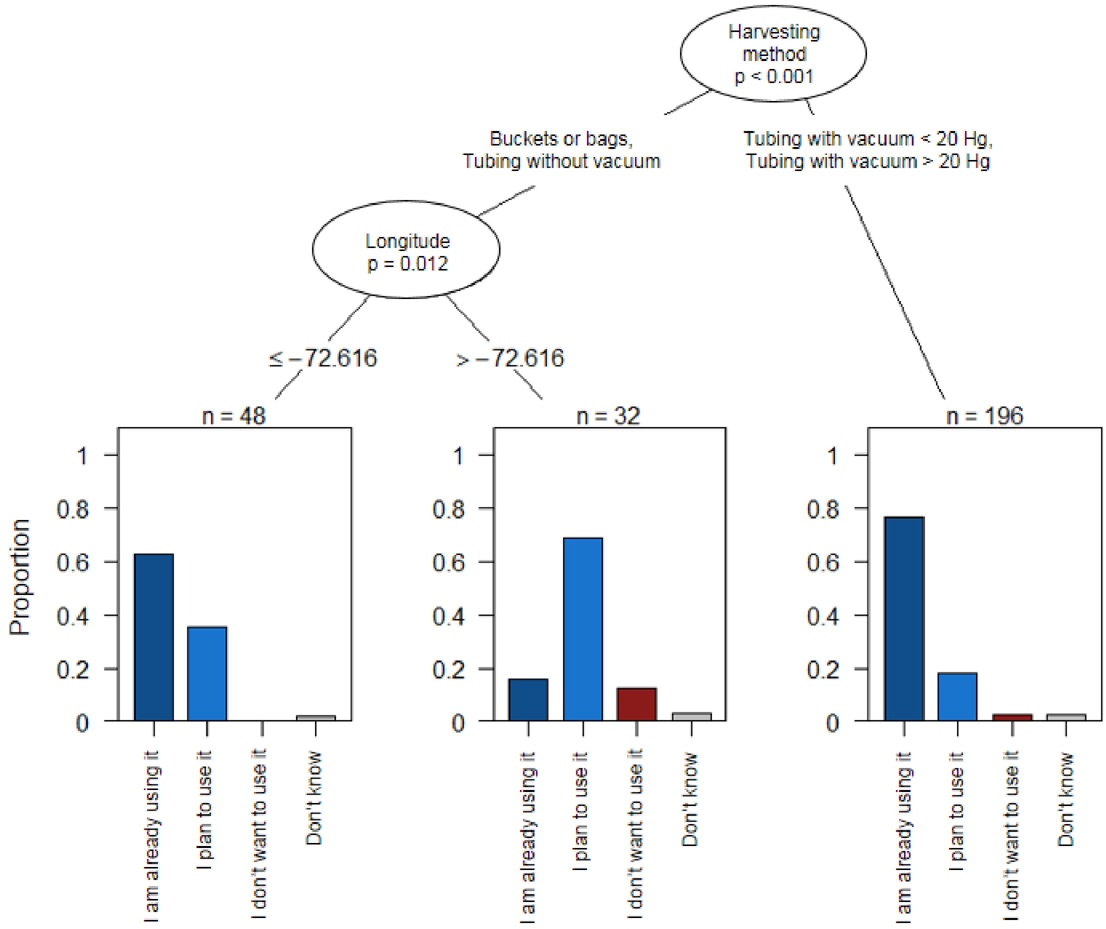

Fig. AJ. CA = 57.1%.

Which of these adaptation measures would you like to use yourself?

Doing sylvicultural management in your sugar bush, for example, maintain the density of trees at a good level or to favor biodiversity

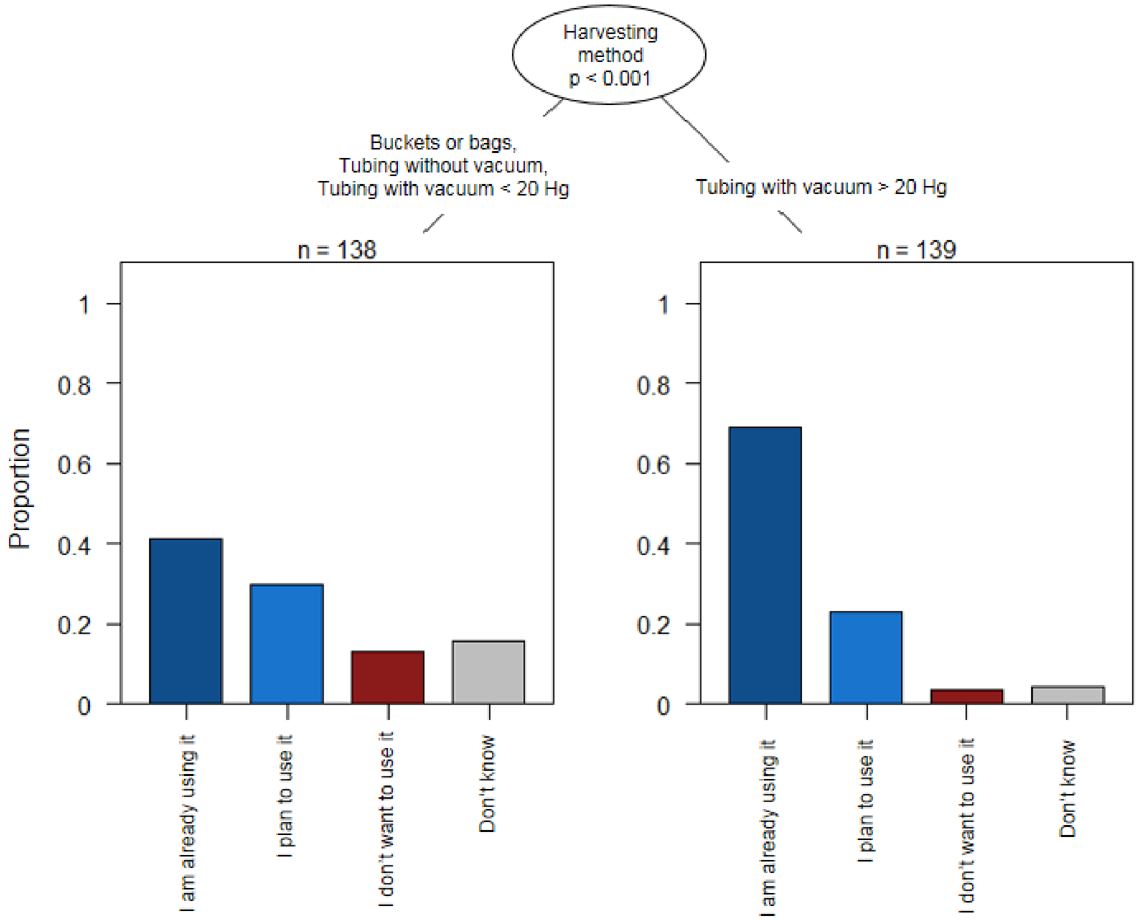

Fig. AK. CA = 55.7%.

Which of these adaptation measures would you like to use yourself?

Adopting strong sanitation practices (tubing and spout cleaning and/or annual spout replacement)

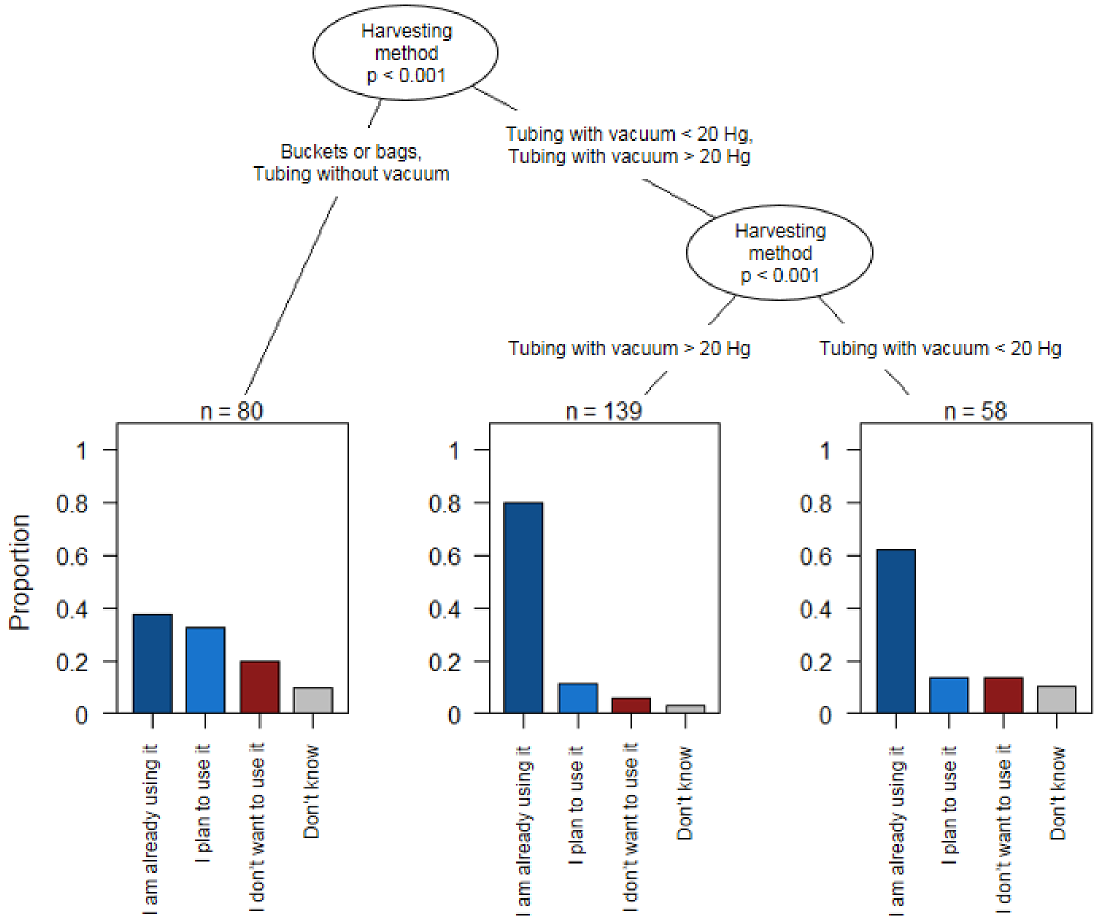

Fig. AL. CA = 55.7%.

Which of these adaptation measures would you like to use yourself?

Tapping earlier in the year

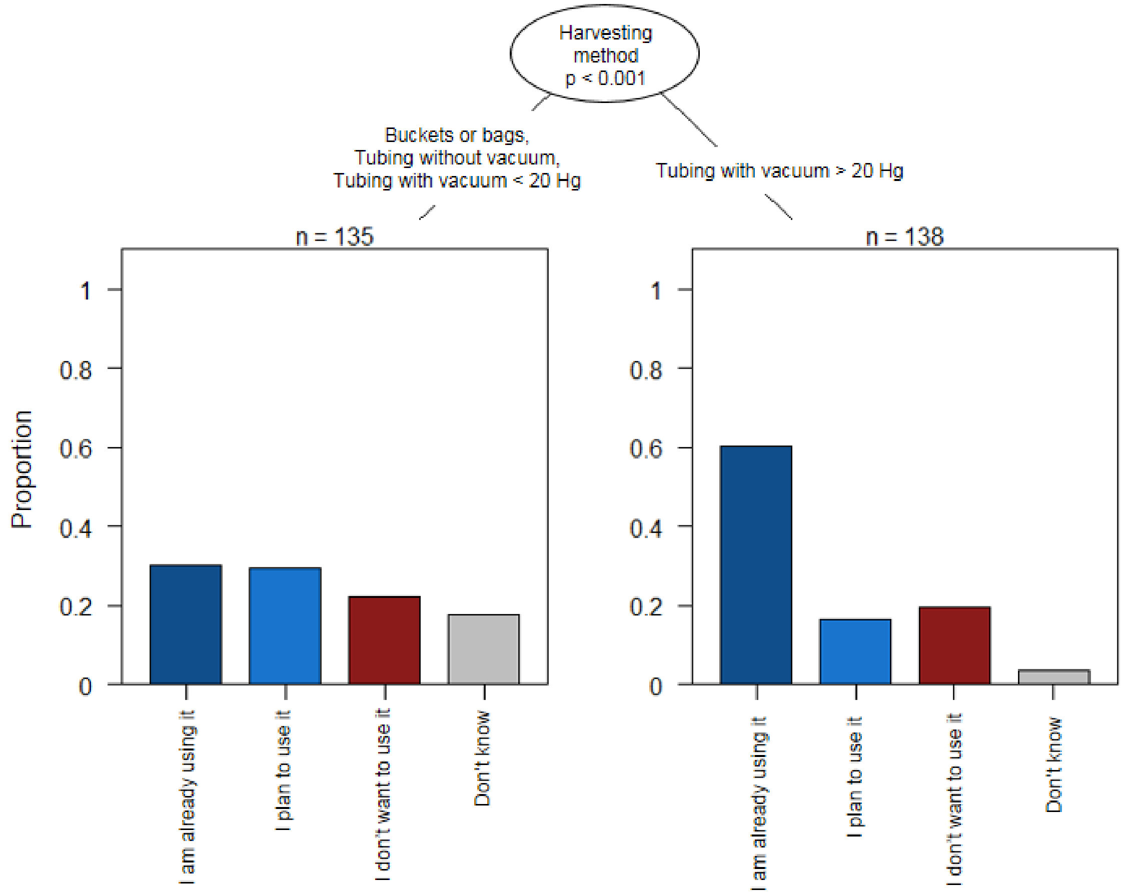

Fig. AM. CA = 34.3%.

Which of these adaptation measures would you like to use yourself?

Installing a high-vacuum tubing system for sap collection

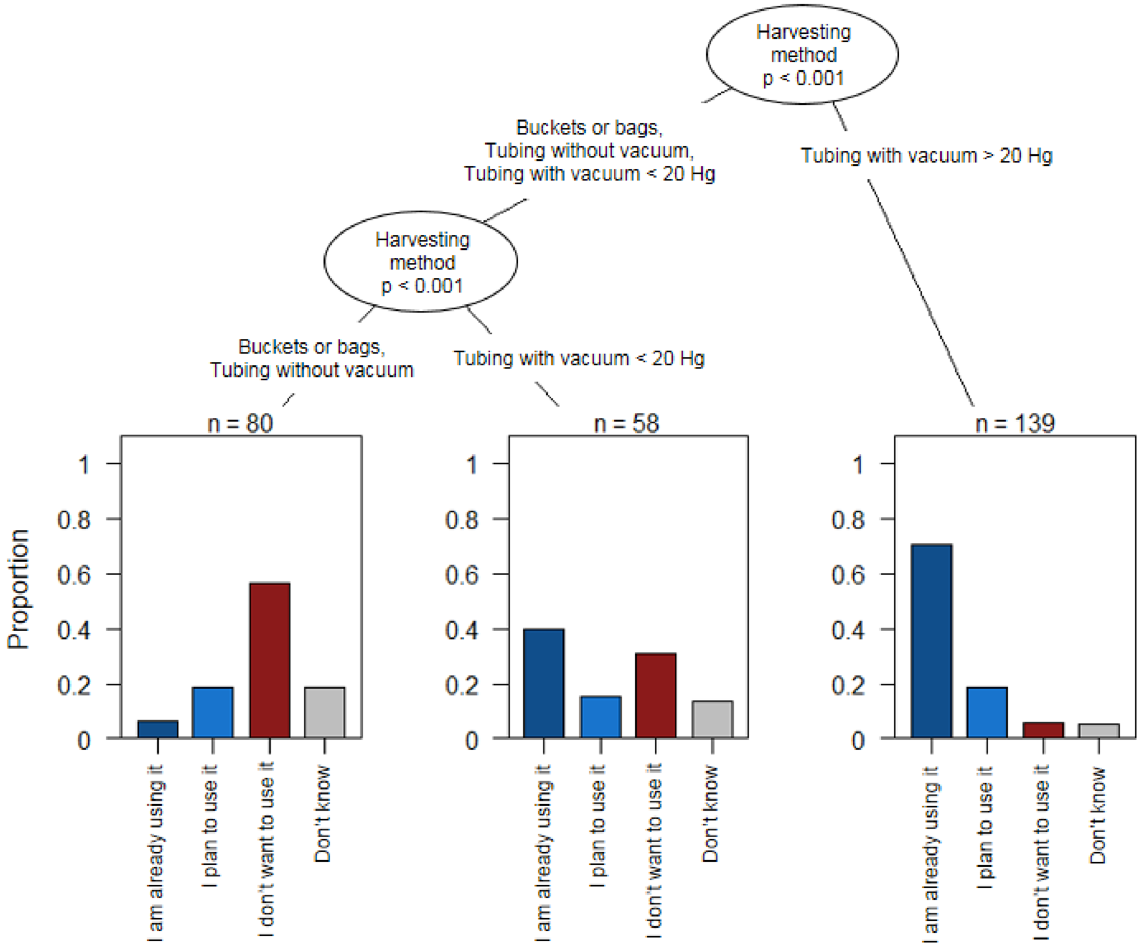

Fig. AN. CA = 58.6%.

Which of these adaptation measures would you like to use yourself?

Liming and fertilizing to limit maple dieback

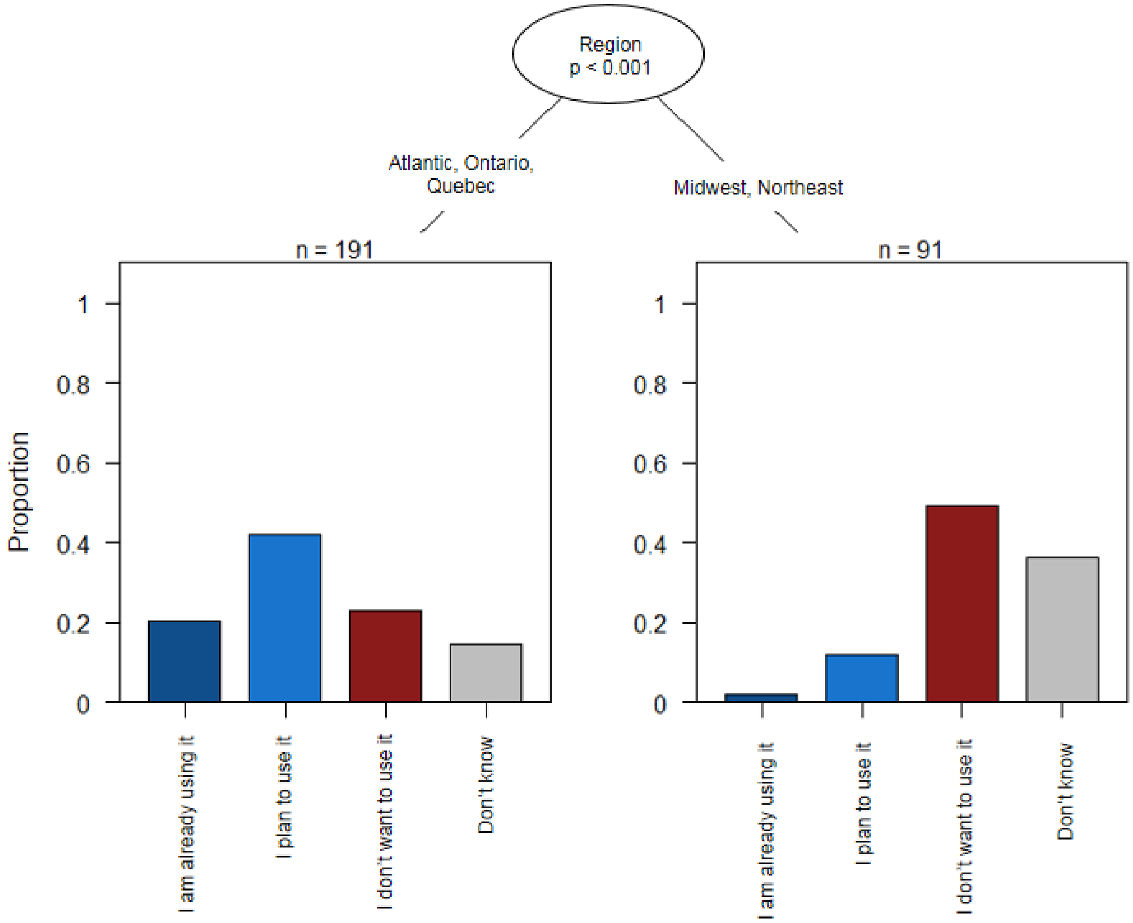

Fig. AO. CA = 37.1%.

Which of these adaptation measures would you like to use yourself?

Tapping red maples

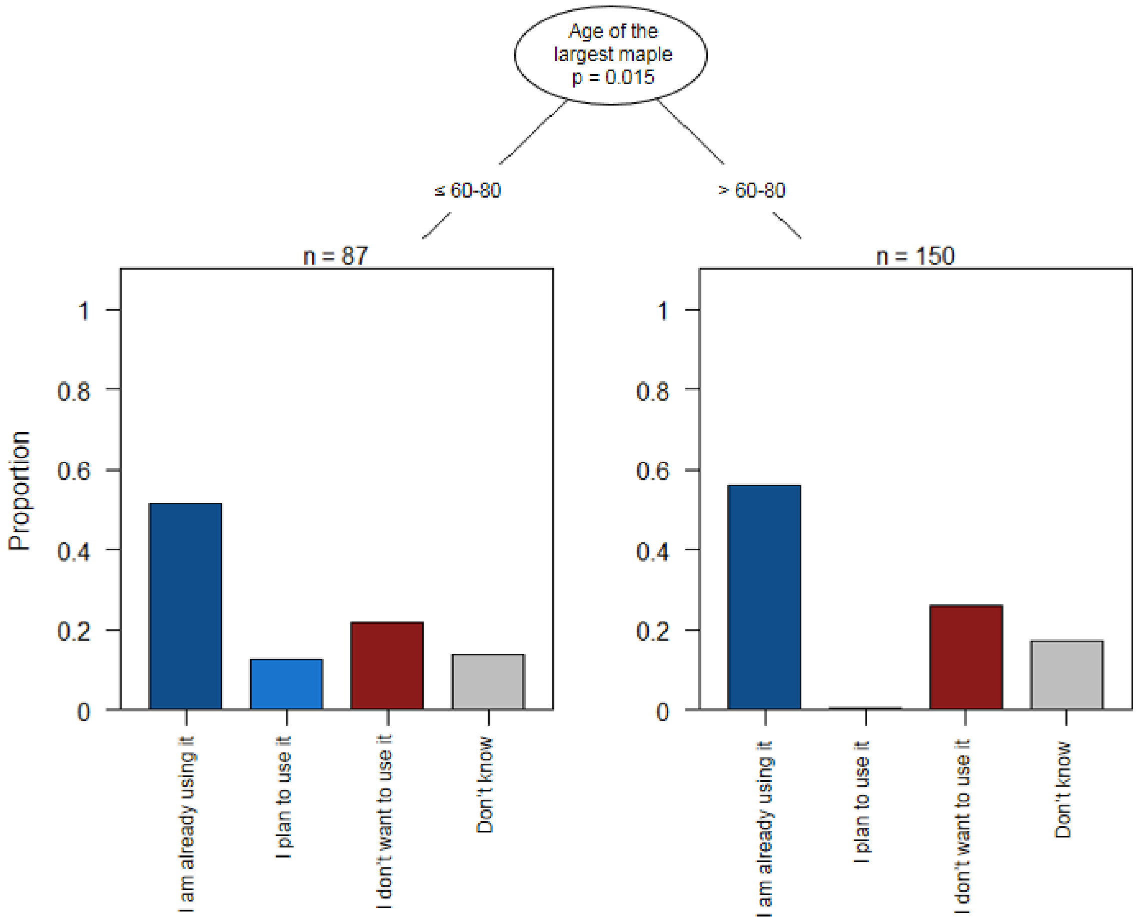

Fig. AP. CA = 57.1%.

Which of these adaptation measures would you like to use yourself?

Using maples adapted to future climate conditions

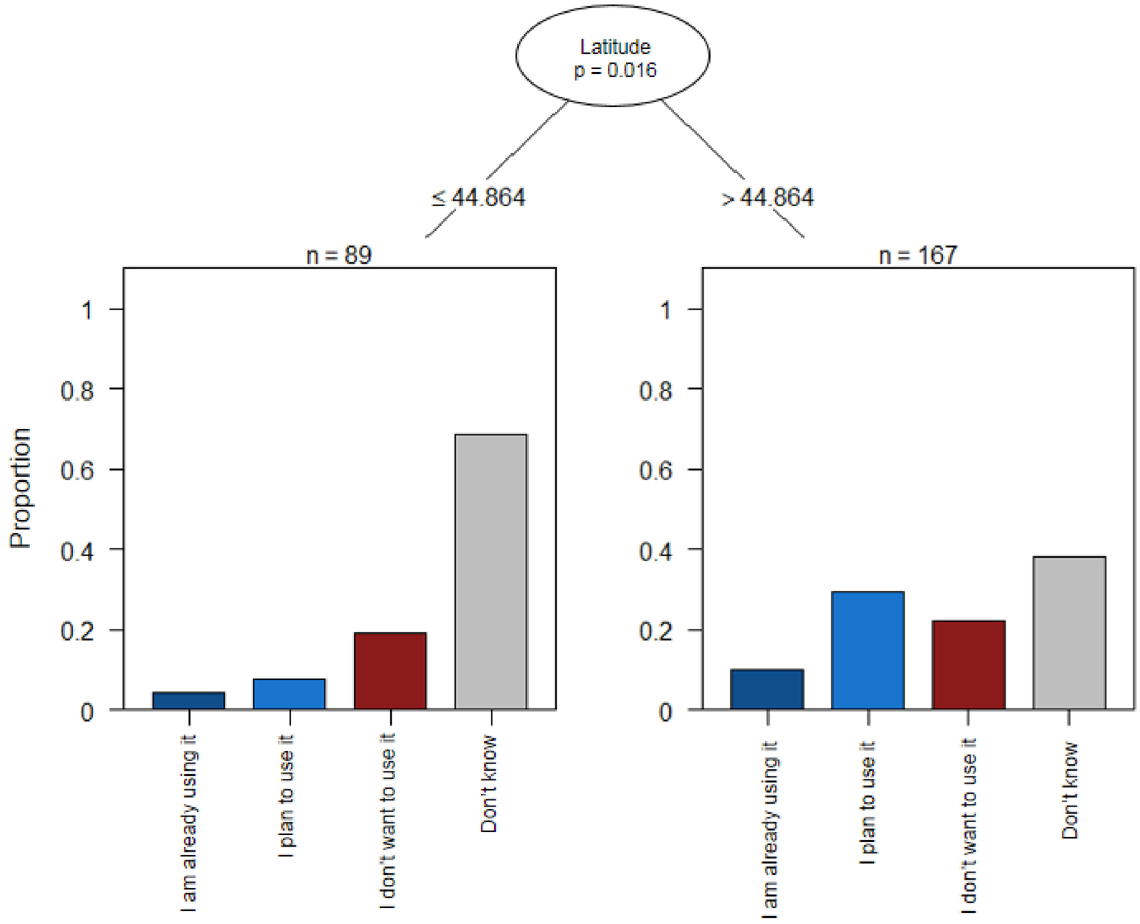

Fig. AQ. CA = 58.6%.

Which of these adaptation measures would you like to see used by the maple syrup industry?

Promoting the distinctive syrup harvested in the very late season

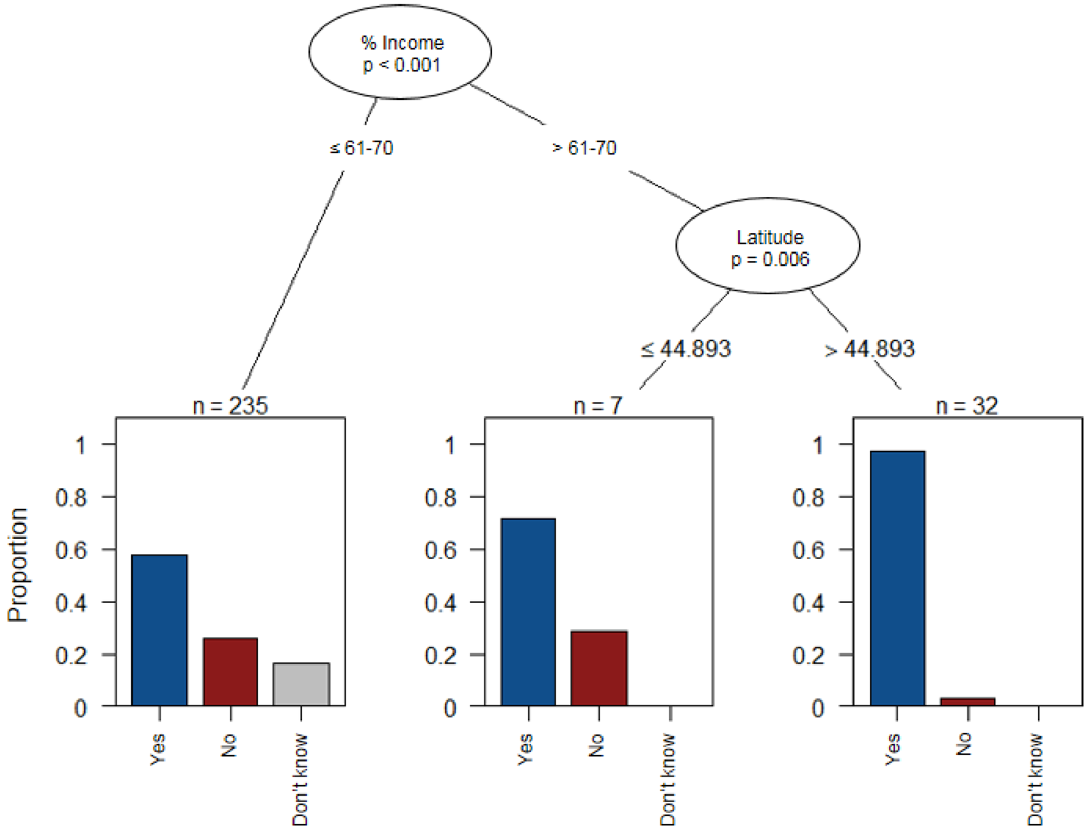

Fig. AR. CA = 57.1%.

To understand how easily your business would be able to adapt (if needed) to any potential impacts of climate change in the future, please indicate if you agree or disagree with each statement below.

If any changes in labor (number of workers, and/or hours worked) are needed due to climate change, my business could quickly get the help it needs to operate

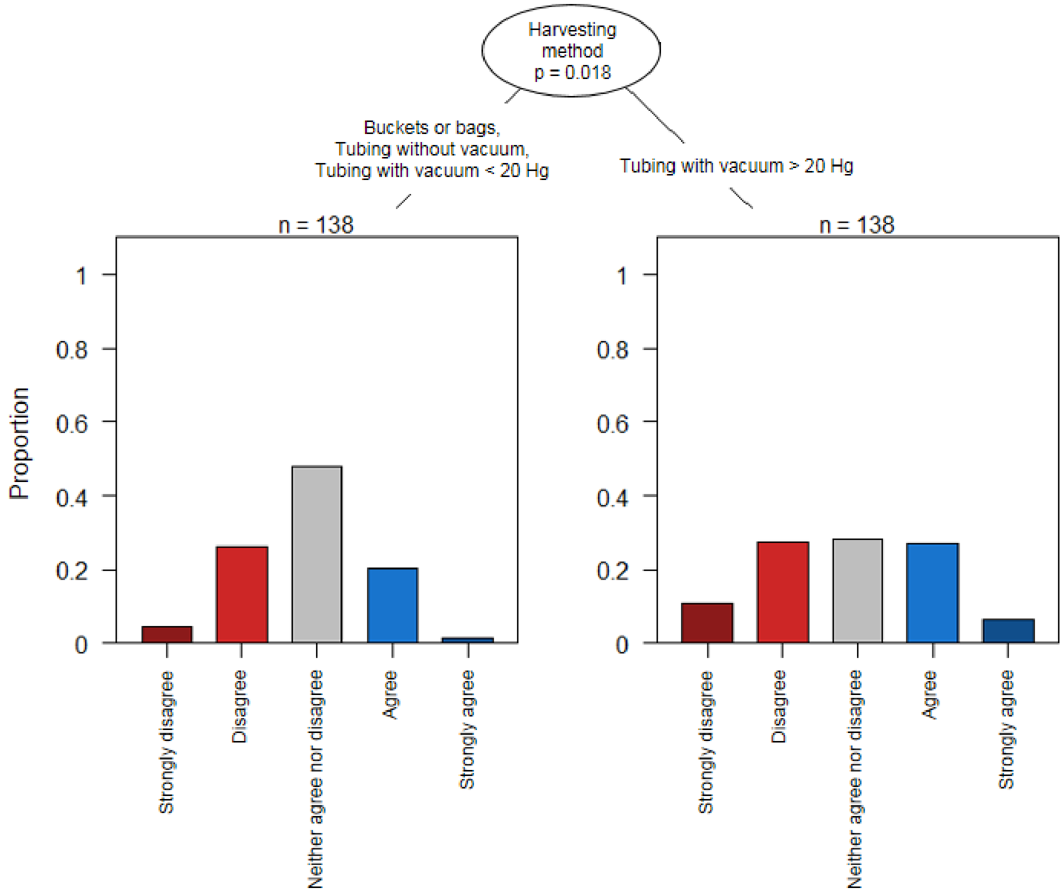

Fig. AS. CA = 34.3%.

To understand how easily your business would be able to adapt (if needed) to any potential impacts of climate change in the future, please indicate if you agree or disagree with each statement below.

If any severe damage to my sugar bush occurred due to climate change, my business could quickly change how it collects and/or obtains sap

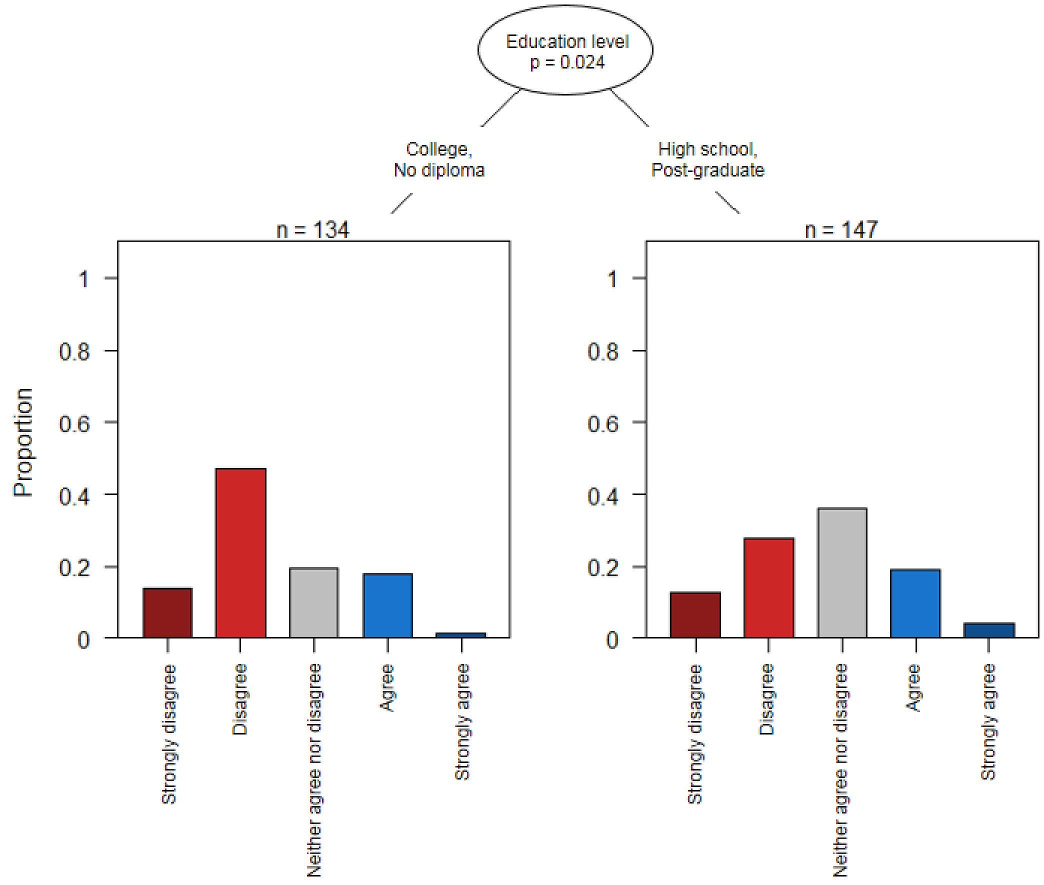

Fig. AT. CA = 22.9%.

Are the following constraints limiting your adoption of new technologies and strategies designed to reduce the impacts of climate change on your business?

Lack of information

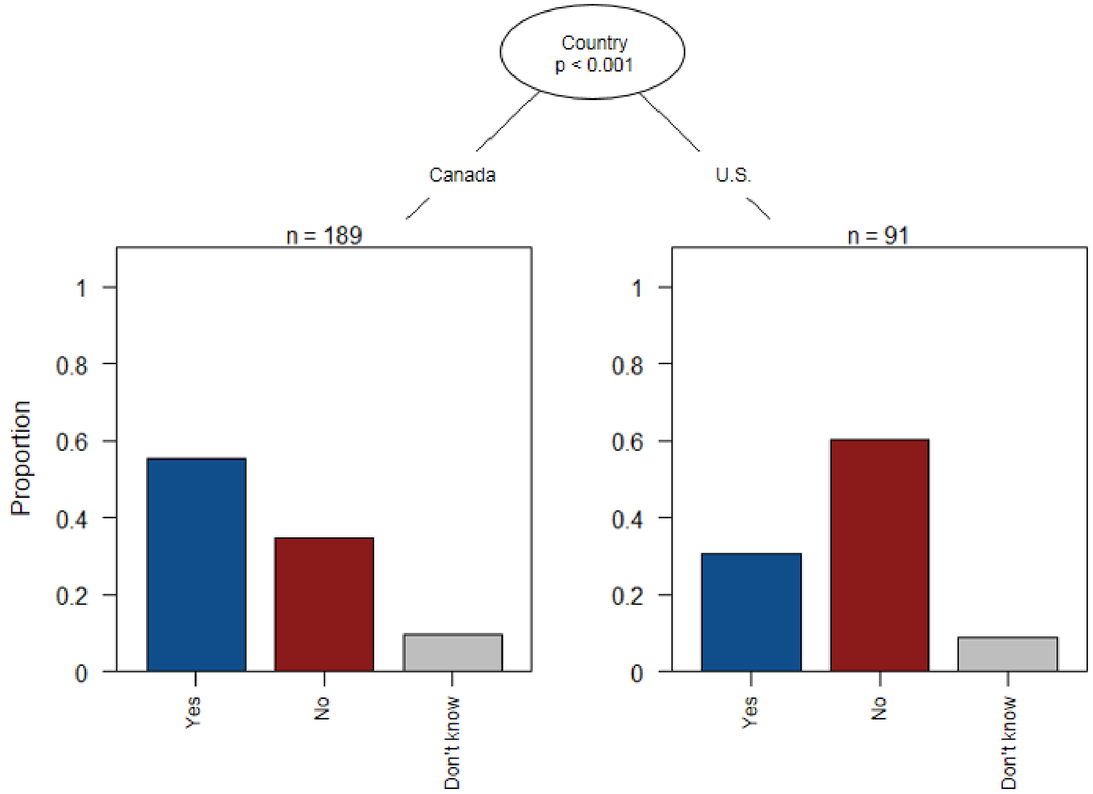

Fig. AU. CA = 55.7%.

Are the following constraints limiting your adoption of new technologies and strategies designed to reduce the impacts of climate change on your business?

Lack of financial means

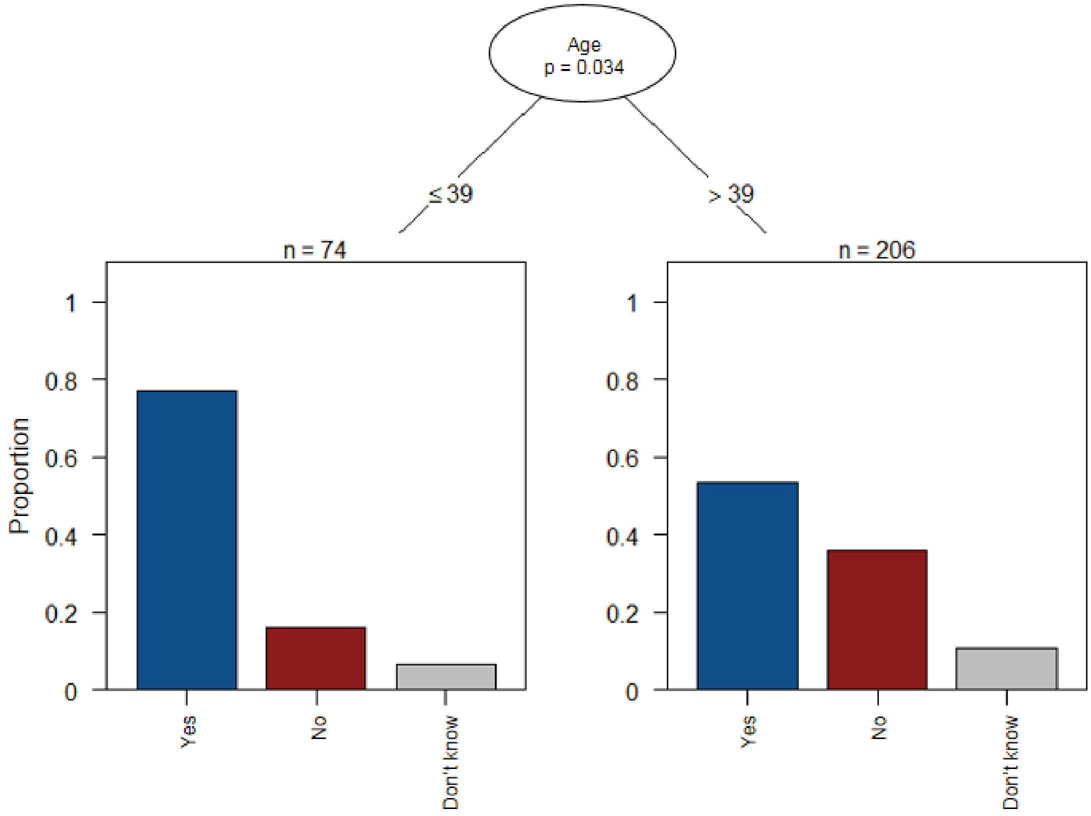

Fig. AV. CA = 58.6%.

Are the following constraints limiting your adoption of new technologies and strategies designed to reduce the impacts of climate change on your business?

Lack of technical support

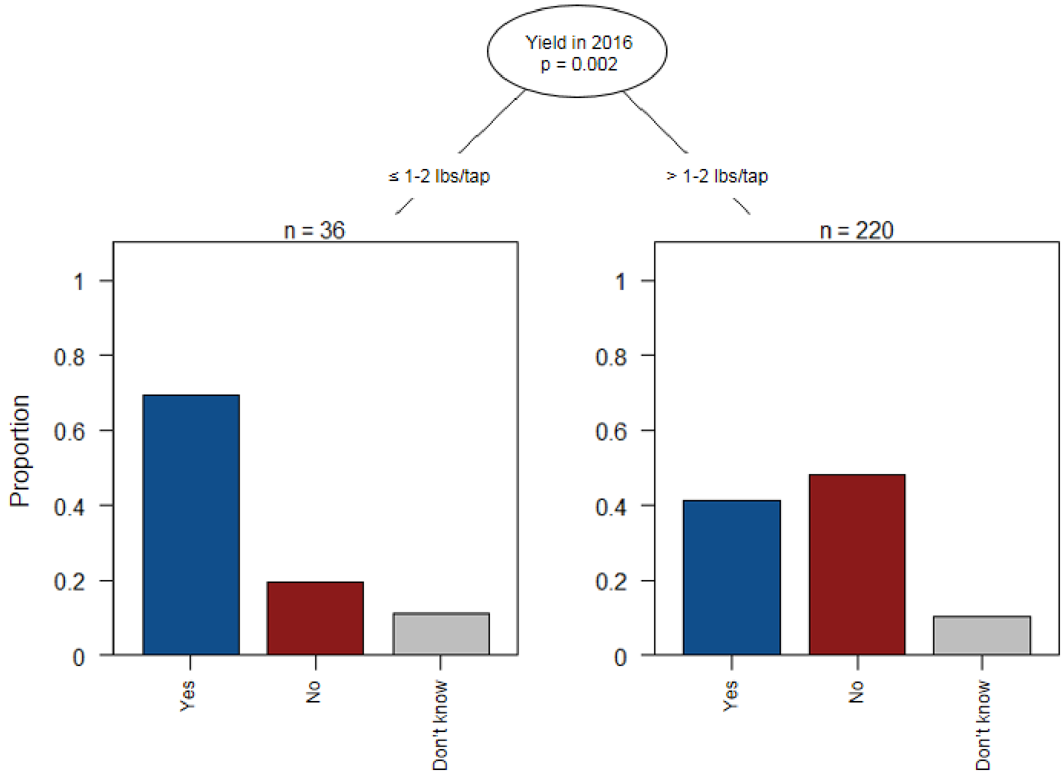

Fig. AW. CA = 38.6%.

Are the following constraints limiting your adoption of new technologies and strategies designed to reduce the impacts of climate change on your business?

I don’t believe climate change will have much impact on my syrup production

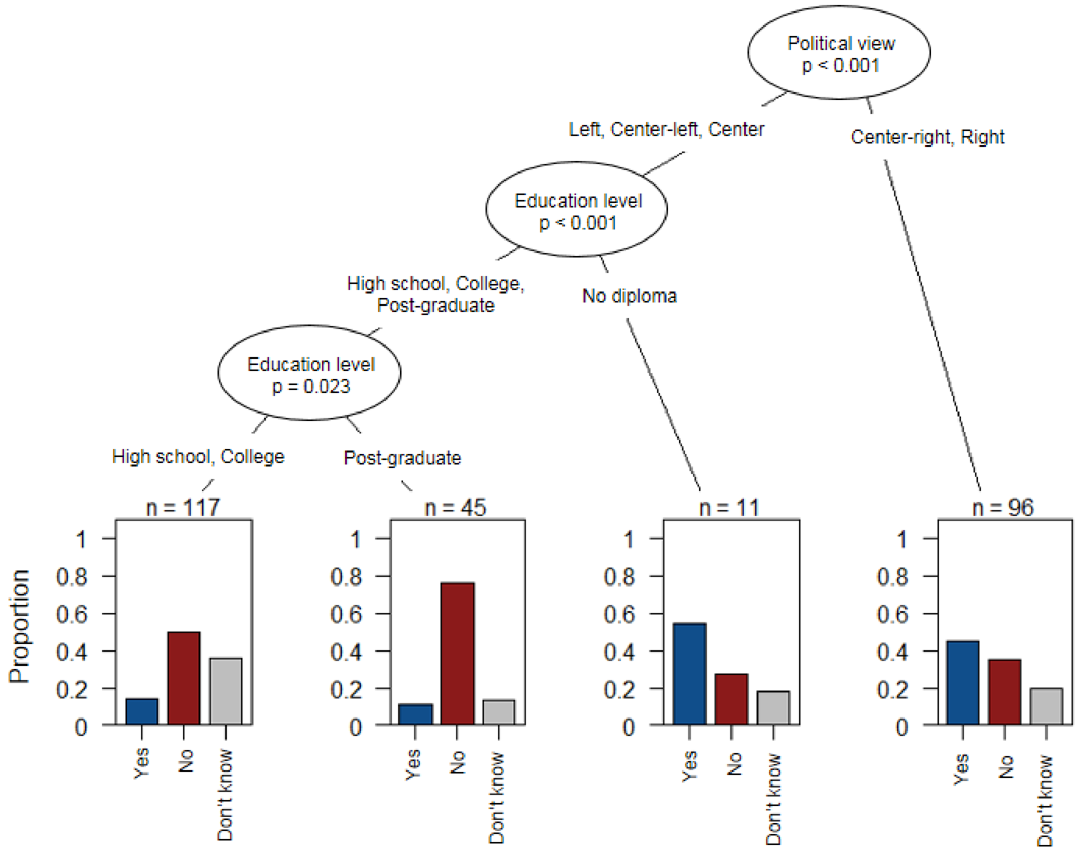

Fig. AX. CA = 31.4%.
